# P4HA2 promotes proliferation, invasion, and metastasis through regulation of the PI3K/AKT signaling pathway in oral squamous cell carcinoma

**DOI:** 10.1038/s41598-024-64264-5

**Published:** 2024-07-01

**Authors:** Zengpeng Chi, Qimin Wang, Xin Wang, Dagang Li, Lei Tong, Yu Shi, Fang Yang, Qingyuan Guo, Jiawei Zheng, Zhenggang Chen

**Affiliations:** 1 Department of Stomatology, Qingdao Huangdao District Central Hospital, Qingdao, 266555 China; 2 Department of Stomatology, Qingdao Hospital, University of Health and Rehabilitation Sciences(Qingdao Municipal Hospital), No.5 Donghai Middle Road, Qingdao, 266071 China; 3 Acupuncture and Tuina Department, Changchun University of Chinese Medicine, Changchun, Jilin, 130117 China; 4 Department of Stomatology, Shenzhen-Shanwei Central Hospital, Sun Yat-sen University, Shanwei, 516699 China; 5 Department of Oromaxillofacial Head and Neck Oncology, College of Stomatology, Shanghai Ninth People’s Hospital, Shanghai Jiao Tong University School of Medicine, No.639, Manufacturing Bureau Road, Huangpu District, Shanghai, 200011 China; 6 Institute of Stomatology, Binzhou Medical University, 256600 Binzhou, China; 7 The affiliated Yantai Stomatological Hospital, Binzhou Medical University, 264000 Binzhou, China

**Keywords:** P4HA2, P13K/AKT pathway, Migration, Invasion, Proliferation, Cancer, Cell biology, Molecular biology, Biomarkers

## Abstract

Proline 4-hydroxylase 2 (P4HA2) is known for its hydroxylase activity, primarily involved in hydroxylating collagen precursors and promoting collagen cross-linking under physiological conditions. Although its overexpression influences a wide variety of malignant tumors' occurrence and development, its specific effects and mechanisms in oral squamous cell carcinoma (OSCC) remain unclear. This study focused on investigating the expression patterns, carcinogenic functions, and underlying mechanisms of P4HA2 in OSCC cells. Various databases, including TCGA, TIMER, UALCAN, GEPIA, and K-M plotter, along with paraffin-embedded samples, were used to ascertain P4HA2 expression in cancer and its correlation with clinicopathological features. P4HA2 knockdown and overexpression cell models were developed to assess its oncogenic roles and mechanisms. The results indicated that P4HA2 was overexpressed in OSCC and inversely correlated with patient survival. Knockdown of P4HA2 suppressed invasion, migration, and proliferation of OSCC cells both in vitro and in vivo, whereas overexpression of P4HA2 had the opposite effects. Mechanistically, the phosphorylation levels of the PI3K/AKT pathway were reduced following P4HA2 silencing. The study reveals that P4HA2 acts as a promising biomarker for predicting prognosis in OSCC and significantly affects metastasis, invasion, and proliferation of OSCC cells through the regulation of the PI3K/AKT signaling pathway.

## Introduction

Oral squamous cell carcinoma (OSCC) is the predominant type of oral cancer, accounting for approximately 90% of all oral cancers. It is characterized by rapid progression, strong invasion, metastasis, and poor prognosis^1^. Due to the critical location of OSCC, patients frequently experience difficulties with articulation, chewing, and swallowing post-surgery, significantly reducing their quality of life^2,3^. Despite significant advancements in OSCC treatment methods such as radiotherapy, chemotherapy, and surgery in recent years, the high surgical risk, costly treatments, and severe side effects have not substantially improved the survival rates of patients with advanced OSCC after surgery^4,5^, which remains below 50% over five years^6^. Consequently, there is an urgent need to develop novel and effective strategies to improve patient outcomes. Given that uncontrolled proliferation and metastasis, particularly to the lungs, are primary causes of death in OSCC patients, it is crucial to explore the mechanisms of invasion, migration, and proliferation in OSCC and identify sensitive genes that could guide early diagnosis and treatment.

Proline 4-hydroxylase 2 (P4HA2), a critical enzyme in collagen formation, catalyzes the hydroxylation of proline residues in collagen to form hydroxyproline. This modification is crucial for establishing the correct three-dimensional structure of collagen^7^. Additionally, P4HA2 influences cancer cell behavior by enhancing collagen production^8^. It is closely associated with hypoxia, collagen deposition, glycolysis, and the migration and invasion of cancer cells^8,9^. Over recent years, P4HA2 has been aberrantly overexpressed in various tumors, accelerating their malignant progression, including in B-cell lymphoma, breast cancer, and prostate cancer^10–12^. In B-cell lymphoma, P4HA2 hydroxylates carabin protein, promoting its degradation. This degradation enhances the activation of the Ras/ERK signaling pathway, thereby increasing the proliferation of B-cell lymphoma cells^11^. Furthermore, studies have shown that P4HA2 promotes collagen deposition, which facilitates the proliferation, invasion, and metastasis of breast cancer cells^12^. However, the roles of P4HA2 in OSCC have been infrequently investigated, and its biological functions and specific molecular mechanisms in OSCC remain unclear.

In this study, we predicted the expression of P4HA2 and its prognostic implications using various databases. We then explored its biological functions and potential molecular mechanisms in OSCC using models of P4HA2 knockdown and overexpression. Our results suggest that P4HA2 overexpression is significantly associated with the progression of OSCC, whereas P4HA2 knockdown inhibits the proliferation, migration, and metastasis of OSCC cells in vitro and in vivo. Additionally, the PI3K/AKT signaling pathway appears to influence these processes. This study may provide a new therapeutic target for OSCC.

## Material and method

### Bioinformatics analysis

We analyzed P4HA2 expression in adjacent non-cancerous tissues and in OSCC samples using data from The Cancer Genome Atlas (TCGA; https://portal.gdc.cancer.gov/repository), the Tumor Immune Estimation Resource (TIMER; http://timer.comp-genomics.org/), and the University of Alabama at Birmingham Cancer Data Analysis Portal (UALCAN; http://ualcan.path.uab.edu/analysis.html). RNA-sequencing data (FPKM values) were analyzed using the EdgeR package in R (version 3.6.1)^13^. We also used GEPIA (http://gepia.cancer-pku.cn/) and Kaplan–Meier plotter (https://kmplot.com/analysis/) to examine the relationship between clinicopathological features of OSCC and P4HA2 expression.

### Patients and tissue samples

We collected 44 fresh frozen OSCC tissue samples from patients (mean age 63; 23 males and 21 females) who underwent surgery at Qingdao Municipal Hospital Affiliated to Qingdao University between 2015 and 2018. The clinicopathological features including lymph node metastasis, tumor grade, stage, gender, and age were recorded. All procedures were conducted with patient consent, and the study was approved by the Qingdao Municipal Hospital Affiliated to Qingdao University.

### Cell culture and reagents

SCC-9 and SCC-25 cells, provided by Professor Zheng from the Department of Oromaxillofacial Head and Neck Oncology at Shanghai Ninth People’s Hospital, China, were cultured in Dulbecco's Modified Eagle's Medium (DMEM, GIBCO, US) supplemented with 100 U/mL streptomycin, 100 U/mL penicillin, and 10% fetal bovine serum (FBS, Sciencell, US). Cells were maintained at 37 °C in a 5% CO_2_ environment. When cells reached 80%-90% confluence, they were subcultured at a ratio of 1:4 for further experiments. Additionally, the P4HA2 enzymatic agonist Isosaponarin was purchased from MedChemExpress (HY-N2589, New Jersey, US).

### Cell transfection and stable cell lines Construction

We transfected all cells with specific shRNA-P4HA2-Lentivirus to knockdown P4HA2 or scrambled shRNA-GFP-Lentivirus (Guangzhou Yuanjing Biotechnology Co., Ltd, Guangzhou, China) at a multiplicity of infection of 50 for 24 h in culture medium containing 5 mg/mL polybrene when the cells reached 30% confluence. Stable cell lines were selected using puromycin post-transfection. Additionally, Lipofectamine 3000 was diluted and mixed with plasmid vectors pcDNA3.1-p4HA2 or pcDNA3.1 (Miaolingbio.lnc, Wuhan, China) according to the manufacturer's instructions, and then added to the culture medium for transfection. This experiment included the sh-P4HA2 group, the sh-GFP group, the pcDNA3.1-p4HA2 group, and the pcDNA3.1 group. (Supplemental Table 1 lists the interference sequences).

### qRT‑PCR

Total RNA was extracted from a variety of cells using the TaKaRa MiniBEST Universal RNA Extraction Kit (TakaRa, Japan). Reverse transcription was performed using the PrimeScript RT Reagent Kit with gDNA Eraser (TakaRa, Japan). The expression level of P4HA2 was quantified by real-time PCR (qRT-PCR) using SYBR Premix EX Taq II (Takara, China) under the following thermal cycling conditions: 60 °C for 30 s, followed by 40 cycles of 95 °C for 5 s, and 95 °C for 30 s. Supplemental Table 2 lists the primer sequences^9^. mRNA levels were quantified using the Comparative threshold cycle (Ct) method and the 2^–∆∆Ct^ analysis method.

### Western blotting test

Cells were lysed using RIPA buffer containing 1% protease inhibitor and 1% phosphatase inhibitor (Beyotime, China), then centrifuged at 4 °C, 12,000× g for 10 min. The supernatant was collected as the total protein. Protein concentration was determined using the BCA Protein Assay Kit (Beyotime, China), following the method described by Tan et al.^14^. Equal amounts of protein samples were separated by 10% SDS-PAGE and transferred onto polyvinylidene difluoride (PVDF) membranes (Roche, US). Membranes were blocked with 5% bovine serum albumin for 1 h at room temperature. Overnight incubation at 4 °C with primary antibodies followed, including anti-phospho-protein kinase B (p-AKT, 1:2000, Cell Signaling Technology), anti-matrix metalloprotease-2 (MMP-2, 1:1000, Proteintech), anti-matrix metalloprotease-9 (MMP-9, 1:1000, Proteintech), anti-Survivin (1:1000, Proteintech), anti-GAPDH (1:1000, Beyotime), anti-Cyclin D1, anti-AKT, anti-PI3K, and anti-phospho-PI3K (p-PI3K, 1:1000, Cell Signaling Technology), and anti-P4HA2 (1:1000, Proteintech). The membranes were washed three times for 10 min each with TBST, then incubated with HRP-conjugated anti-rabbit IgG secondary antibodies (1:50,000, Cell Signaling Technology) in 5% BSA for 2 h at room temperature. Protein band intensity was analyzed using Image J software (version 1.51j8; National Institutes of Health; https://imagej.net/software/imagej/).

### Cell invasion test

Add 20 μL of Matrigel (Becton–Dickinson, the US) diluted 1:8 (Matrigel:DMEM) into the BioCoat Corning Matrigel Invasion Chamber (8 μm pore size; Becton–Dickinson, the US). We placed the Transwell chamber containing Matrigel on a 24-well plate. The transfected cells (2 × 10^5^ cells in 200 μL of medium) were placed in the upper chamber. In the lower chamber, we filled it with 500 μL of complete culture medium containing 10% FBS to serve as an attractant. After 48 h, we fixed the invaded cells with 4% paraformaldehyde and stained them with 0.1% crystal violet. The bottom membrane of the chamber was examined under a microscope at 40× and 200× magnification, and 5 random fields were selected for photography. Representative 40× images are shown in Figure 4B and 200× images in the Supplementary Figure S1. The number of cells that had passed through the bottom membrane was recorded using ImageJ software, and the average value was calculated. The experiment was repeated three times.

### Cell scratch test

We cultured all cells on a six-well plate and created scratches using a 20 μL pipette tip. PBS was used to wash away the floating cells three times. Cells were then cultured in DMEM medium without FCS. Furthermore, the distance between the two edges of the scratch was measured under the microscope 48 h later. We observed the scratch wound areas immediately and 48 h after the scratch under the microscope. Cell migration was quantified as the percentage of wound healing relative to the initial wound area.

### Cell viability test

Seed all experimental cells at a density of 2 × 10^5^ cells per well and culture the cells in medium supplemented with 10% FBS for 0, 24, and 48 h. Then, add 10 μL of CCK-8 to each well and incubate at 37 °C for 2 h. Afterwards, measure the optical density (OD) of each well using a microplate reader at 450 nm. After the measurement, return the 96-well plate to the incubator until the next test point. Each group had five replicates at each time point.

#### Cell cycle analysis and cell apoptosis test

All cells were seeded in a 6-well culture plate, with each well reaching a density of 2 × 10^5^ cells and a total volume of 2 mL. The cells were then fixed using 70% cold ethanol at − 20 °C overnight. The fixed cells were washed twice with precooled PBS and stained with a solution containing propidium iodide (PI, 50 μg/mL; Sigma Aldrich, St. Louis, the US) and RNase A (100 μg/mL; Sigma Aldrich, St. Louis, the US) at 4 °C for 30 min. A flow cytometer (Beckman Coulter, the US) was used to quantify the DNA content of the cells. Data were obtained using FlowJo software (version V10; https://www.flowjo.com/solutions/flowjo/downloads). For the apoptosis test, cells were treated with 0.25% trypsin (without EDTA), centrifuged at 500 g for 5 min, and then subjected to staining with Annexin V-PE and/or PI for 15 min at room temperature. A FACSCalibur flow cytometer was used for analysis.

#### IHC staining

We fixed OSCC and adjacent tissues, followed by an embedding process as per standard protocols. The embedded tissues were sectioned into 4 μm slices and subjected to staining using a conventional immunohistochemistry procedure. To block endogenous peroxidase, sections were treated with hydrogen peroxide. The sections were then incubated overnight at 4 °C with P4HA2 antibody (1:200, Proteintech). Following this, sections were incubated with an anti-rabbit secondary antibody for 30 min at room temperature. Color development was achieved using 3-diaminobenzidine tetrahydrochloride (DAB reagent kit, ZLI-9017, ZSGB-BIO, China) and counterstained with hematoxylin. Staining intensity was evaluated under a microscope, identifying cells with brown cytoplasm as positive. Two pathologists blindly assessed the staining intensity of the slides without prior knowledge of the patients' basic information or clinical features. During high-power microscopic examination, five fields were selected, and the number of positive cells was counted to obtain an average.

#### Xenograft tumor model test

BALB/c nude mice (female, aged 4–6 weeks) were provided by Vital River Laboratory Animal Technology Co., Ltd. (Beijing, China). Mice were maintained under standard pathogen-free conditions and randomly assigned to two groups, each consisting of six mice. Each mouse was injected subcutaneously on the left side with 5 × 10^6^ sh-P4HA2 or sh-GFP cells. Body weight and tumor size were monitored post-injection. Tumor size was measured every five days using calipers, and volume was calculated using the formula: V = 1/2 × A × B^2^, where A and B represent the tumor's largest and smallest diameters, respectively^15^. After 26 days, the mice were dissected, and tumors were stained with hematoxylin–eosin (H&E). For the pulmonary metastasis model, two groups of BALB/c nude mice were injected via the tail vein with 1 × 10^6^ sh-P4HA2 or sh-GFP cells. Four weeks later, lung tissues were harvested, fixed in formalin, and the lymph nodes were counted.

Animal husbandry and experimental procedures were approved by the Ethics and Humanities Committee of Qingdao Municipal Hospital (Shandong, China) and were performed in full compliance with relevant ethical guidelines (Approval ID: 2,023,108).

#### H&E staining

Lung specimens and subcutaneous tumors were fixed in 4% paraformaldehyde, dehydrated with graded ethanol, cleared with xylene, infiltrated with wax, embedded in paraffin, sectioned, and routinely stained with H&E for microscopic observation (Olympus, BX61, Japan).

#### Statistical analysis

Data analysis was performed using SPSS (version 16.0; https://www.ibm.com/spss). Results are expressed as means ± standard deviation (SD). Multiple comparisons were conducted using one-way ANOVA followed by LSD as a post-hoc test. Differences between two groups were analyzed using the Student's t-test. Normal distribution was assessed using the Kolmogorov–Smirnov and Shapiro–Wilk tests. For non-normally distributed data, comparisons between two groups were made using the Mann–Whitney test, and among multiple groups using the Kruskal–Wallis test. Overall survival (OS) of patients with different P4HA2 levels (using median as cutoff) was compared using the Kaplan–Meier method and log-rank test. Non-significant differences were denoted as 'ns'. Statistical significance was considered at *P < 0.05; **P < 0.01; ***P < 0.001;****P < 0.0001. Each experiment was conducted three times.

## Results

### P4HA2 achieves high expression in OSCC tissues in contrast to normal tissues

**Figure 1:**
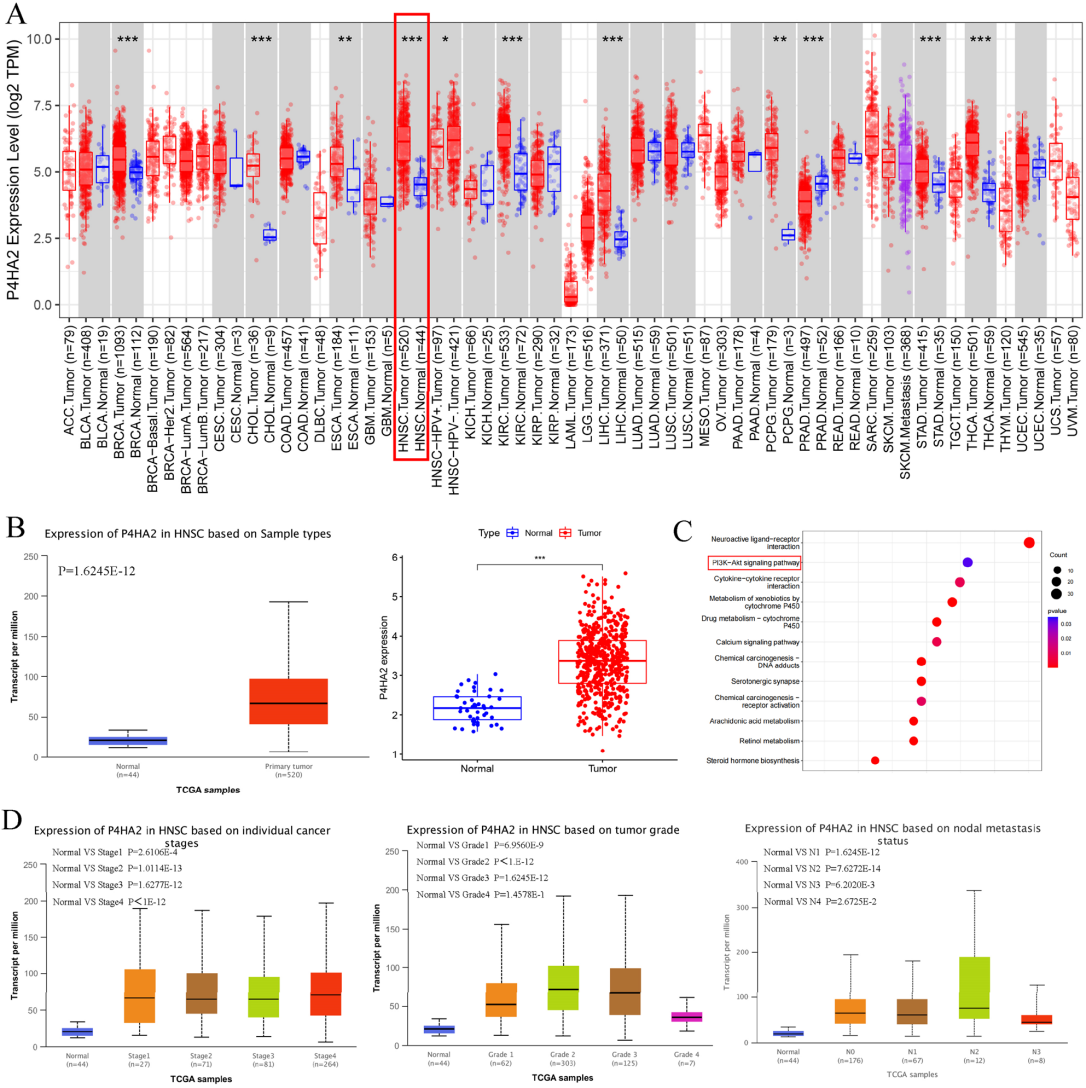
Investigation based on database in terms of OSCC’s P4HA2 expression and the correlation of the expression with pathological feature, and pathway enrichment of P4HA2. (**A**, **B**) Investigation using UALCAN, TIMER, and TCGA databases regarding the expression levels of P4HA2 in OSCC and normal tissues, analyzed separately. In contrast to the results in normal tissues, OSCC tissues exhibited higher P4HA2 expression. (**C**) We analyzed the potential functions of P4HA2 through the GO database. The data indicated that P4HA2 was notably enriched in the PI3K/AKT signaling pathway. (**D**) Investigation based on the UALCAN database of P4HA2 levels in relation to clinical stage, pathological grades, and lymph node metastasis. The data indicated that P4HA2 expression correlated closely with clinical stage, pathological grades, and lymph node metastasis. *:*p* less than 0.05, **:*p* less than 0.01, ***:*p* less than 0.001, ****:*p* less than 0.0001.

We utilized the UALCAN, TIMER, and TCGA databases to investigate P4HA2 expression in normal and OSCC tissues (Fig. 1A,B). The data suggest that, in contrast to normal tissues, P4HA2 expression is significantly higher in OSCC tissues. Additionally, the UALCAN database indicates a significant correlation between P4HA2 expression and clinical stage, pathological grades, and lymph node metastasis (Fig. 1C). We also conducted a function enrichment analysis using the Gene Ontology database (http://geneontology.org/) to explore the potential functions of P4HA2 in depth. The results show that P4HA2 is notably enriched in several pathways, including the Calcium signaling pathway, cytokine-cytokine receptor interaction, and PI3K/AKT signaling pathway (Fig. 1D).

### P4HA2 might be a prognostic biomarker for OSCC

**Figure 2:**
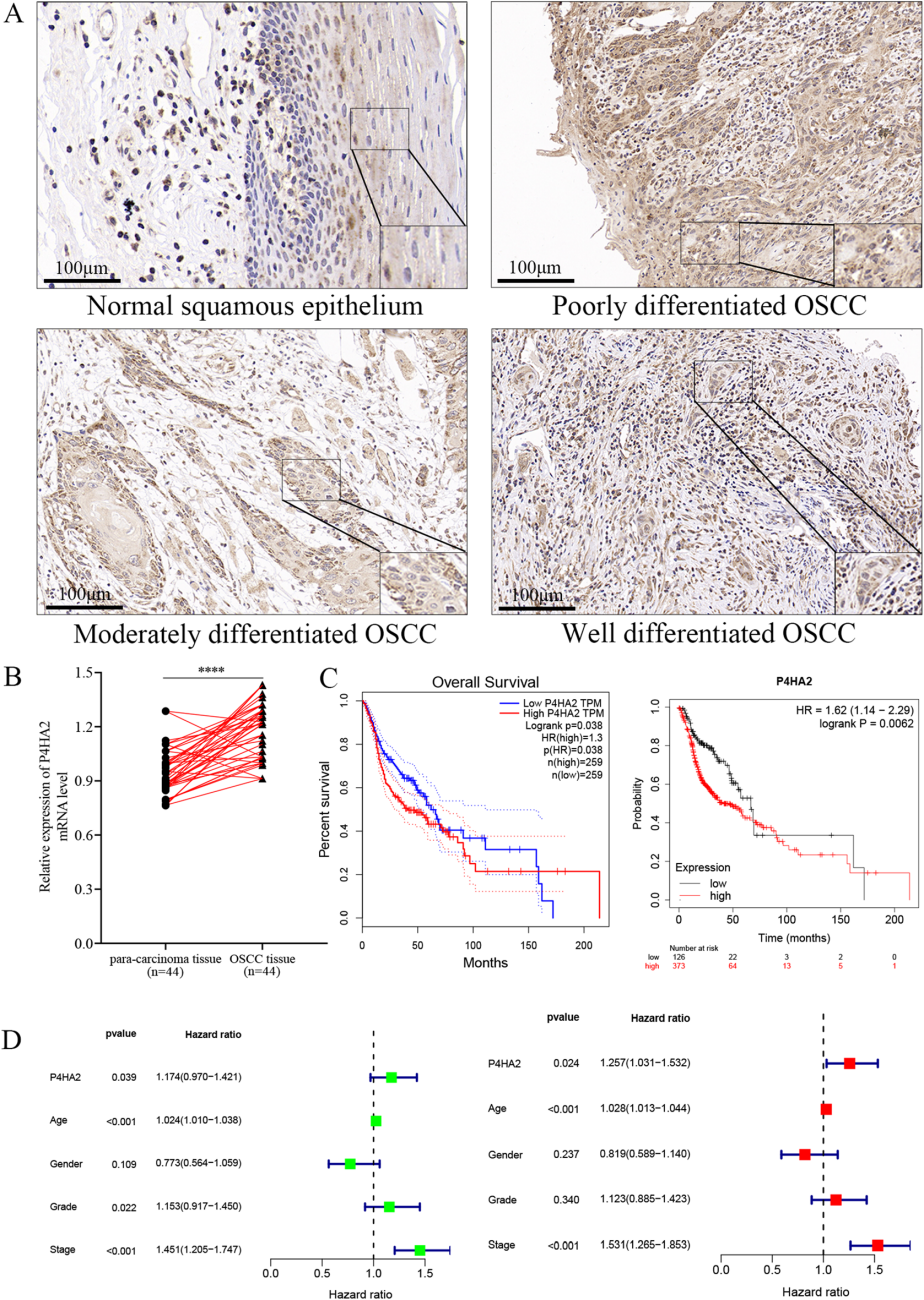
P4HA2 is overexpressed and correlated with poor prognosis in OSCC. (**A**, **B**) qRT-PCR and IHC analysis were employed to analyze P4HA2 expression in OSCC tissues and adjacent normal tissues. The data indicated that P4HA2 was significantly overexpressed in OSCC tissues compared to adjacent normal tissues, and its expression level was significantly correlated with the degree of differentiation in OSCC tissues. (**C**) The K-M plotter and GEPIA database were used to analyze the relationship between P4HA2 expression and the survival of OSCC patients. The data showed that patients with P4HA2 overexpression had poorer overall prognosis compared to those with low expression. (**D**) COX regression analysis was conducted to evaluate clinical features in relation to OS in HNSC. *:*p* less than 0.05, **:*p* less than 0.01, ***:*p* less than 0.001, ****:*p* less than 0.0001.

To validate our findings, we analyzed P4HA2 expression in 44 OSCC tissue specimens using qRT-PCR and IHC analysis. The IHC results show that positive P4HA2 protein expression is characterized by cytoplasmic staining with a brownish-yellow color. P4HA2 expression was negative in well-differentiated OSCC tissues, weakly positive in moderately differentiated tissues, and notably positive in poorly differentiated tissues, indicating a significant correlation with the degree of tissue differentiation (Fig. 2A). Furthermore, The relative expression of P4HA2 mRNA was found to be higher in OSCC tissues compared to adjacent normal tissues (Fig. 2B). Additionally, semi-quantitative scoring analysis revealed a significant correlation between P4HA2 expression and clinical stage, pathological grades, and lymph node metastasis. However, there was little association between P4HA2 expression levels in cancer tissues and patients' gender, age, or smoking history (Supplemental Table 3). Overall, our findings are consistent with the data obtained from the UALCAN database (Fig. 1C). For a more detailed examination of P4HA2's role in OSCC, we used the K-M plotter and GEPIA database to evaluate the prognosis of all OSCC patients. The data indicate that patients with P4HA2 overexpression have a poorer overall prognosis compared to those with low expression (Fig. 2C). Furthermore, high P4HA2 expression, along with OSCC grade and stage, significantly impacts patient survival, as evidenced by univariate analysis results. Multivariate analysis also shows that age, tumor stage, and high P4HA2 expression are correlated with overall survival (Fig. 2D and Supplemental Table 4).

### P4HA2 silencing and overexpression models were constructed

**Figure 3:**
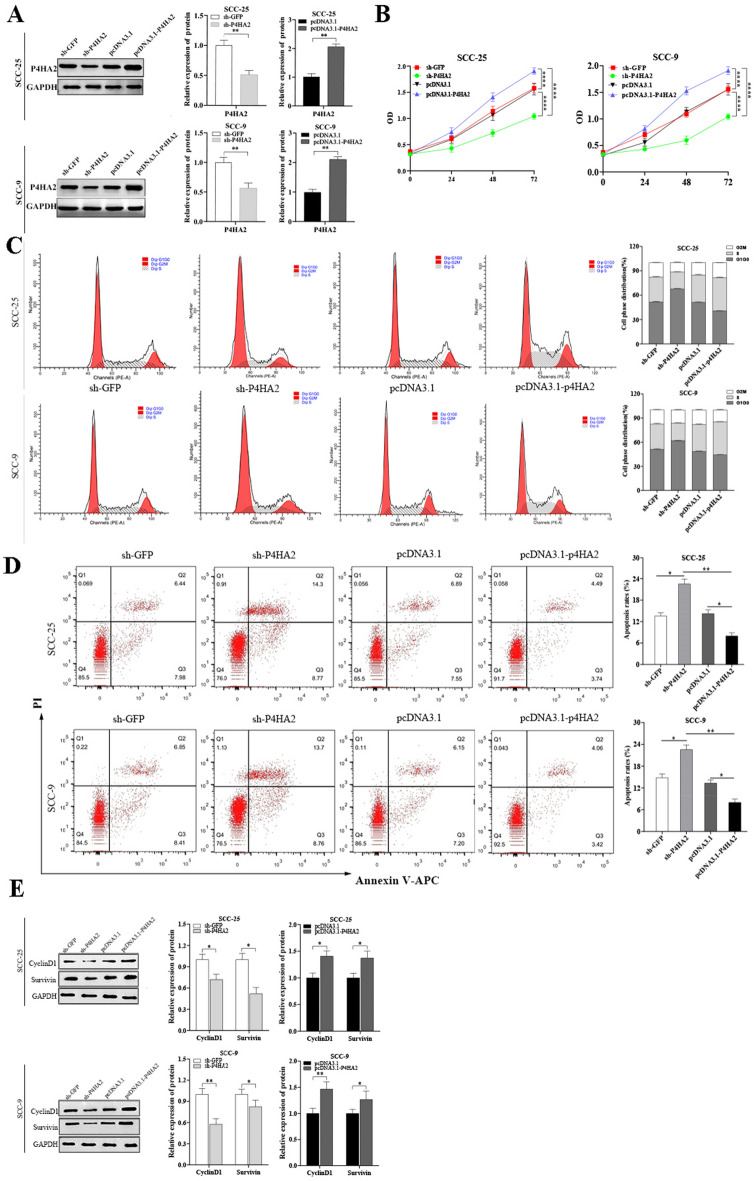
Detection of transfection efficiency of cells and the influence of P4HA2 expression on the OSCC cells’ apoptosis and proliferation. (**A**) qRT-PCR and WB tests were used to measure the transfection efficiency of OSCC cells. (**B**) The CCK-8 test was used to measure the cell growth rates in the sh-P4HA2, sh-GFP, pcDNA3.1-p4HA2, and pcDNA3.1 groups of SCC-25 and SCC-9. The data indicated that P4HA2 could facilitate the proliferation of SCC-25 and SCC-9. (**C**) The cell cycle profile in sh-P4HA2, sh-GFP, pcDNA3.1-p4HA2, and pcDNA3.1 groups of SCC-25 and SCC-9 was investigated by flow cytometry. The data indicated that P4HA2 could affect the cell cycle by regulating the G1G0/S phase. (**D**) Apoptosis in the sh-P4HA2, sh-GFP, pcDNA3.1-p4HA2, and pcDNA3.1 groups of SCC-25 and SCC-9 was investigated through flow cytometry. The data indicated that inhibition of P4HA2 expression could accelerate cell apoptosis. (**E**) WB test was used to measure the protein expression of Cyclin D1 and survivin in the sh-P4HA2, sh-GFP, pcDNA3.1-p4HA2, and pcDNA3.1 groups of SCC-25 and SCC-9. The data indicated that P4HA2 knockdown suppressed the expression of Cyclin D1 and survivin proteins. Conversely, we observed opposite results when P4HA2 was overexpressed. N = 3. *:*p* less than 0.05, **:*p* less than 0.01, ***:*p* less than 0.001, ****:*p* less than 0.0001.

We constructed models of P4HA2 silencing (sh-P4HA2 and sh-GFP) and overexpression (pcDNA3.1-p4HA2 and pcDNA3.1) by transfecting these constructs into OSCC cells. Subsequent qRT-PCR and Western blot tests confirmed the successful construction of these models in SCC-25 and SCC-9 cell lines (Fig. 3A).

### P4HA2 adjusts the OSCC cells’ proliferation

To assess the impact of P4HA2 on the function of SCC-25 and SCC-9 cells, we performed both CCK-8 and apoptosis tests. The CCK-8 test results demonstrate that P4HA2 facilitates cell proliferation (Fig. 3B). Additionally, this study suggests that P4HA2 affects the cell cycle, specifically regulating the transition from G1/G0 to S phase, as shown by flow cytometry (Fig. 3C). Flow cytometry also indicates that inhibition of P4HA2 expression can accelerate cell apoptosis (Fig. 3D). Based on the results of the cell proliferation and apoptosis experiments, we further explored the related molecular mechanisms. The protein levels of Cyclin D1, a cell cycle regulator associated with the G1/G0 to S transition, and survivin, a protein associated with cell apoptosis, were analyzed through Western blot tests. The results indicate that P4HA2 knockdown suppresses the expression of Cyclin D1 and survivin, whereas P4HA2 overexpression leads to increased expression of these proteins (Fig. 3E). Thus, P4HA2 can influence cell apoptosis and proliferation through its regulation of the cell cycle via Cyclin D1 and survivin in OSCC cells.

### P4HA2 adjusts OSCC cells’ metastasis and invasion

**Figure 4:**
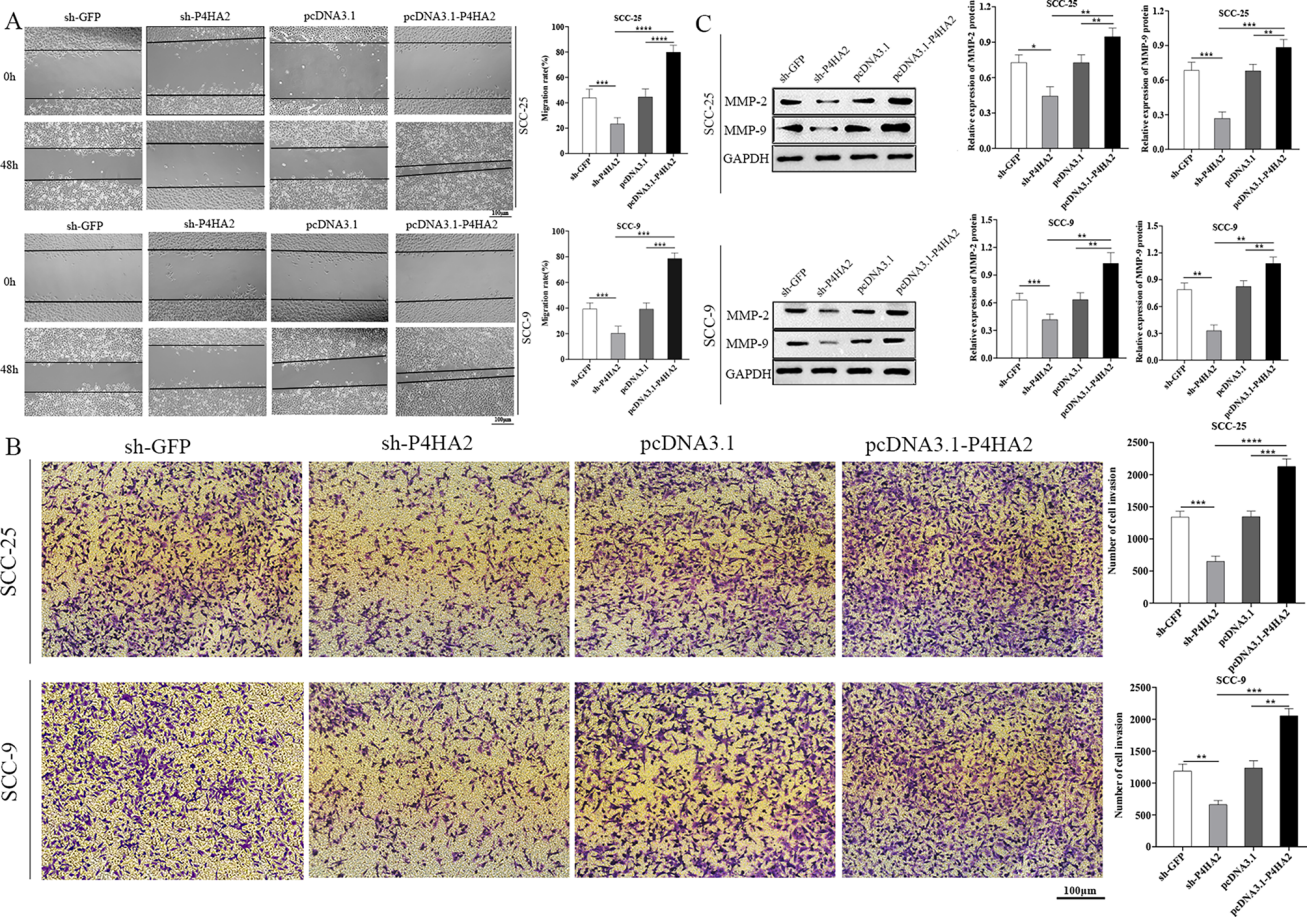
Influence of P4HA2 expression on OSCC cells’ metastasis and invasion. (**A**, **B**) Migration and invasion tests examined the cell migration ability in the sh-P4HA2, sh-GFP, pcDNA3.1-p4HA2, and pcDNA3.1 groups of SCC-25 and SCC-9, analyzed separately. The data indicated that P4HA2 could positively regulate the OSCC cells' ability to migrate and invade. (**C**) A WB test was used to measure MMP-9 and MMP-2 expression in the sh-P4HA2, sh-GFP, pcDNA3.1-p4HA2, and pcDNA3.1 groups of SCC-25 and SCC-9. The data indicated that P4HA2 knockdown suppressed MMP-9 and MMP-2 expression. Furthermore, we found opposite results when P4HA2 was overexpressed. N = 3. *:*p* less than 0.05, **:*p* less than 0.01, ***:*p* less than 0.001, ****:*p* less than 0.0001.

We performed tests in vitro to analyze how P4HA2 affects the biological behavior of OSCC. We performed the transwell invasion test and the scratch test to analyze the influence of P4HA2 on OSCC cells’ migrating and invading processes. The transwell invasion test results indicated that the scratch wound healing time was prolonged after silencing P4HA2 expression. After P4HA2 knockdown, transmembrane cells’ number declined. However, P4HA2 overexpression reversed the results (Fig. 4A,B and and Supplementary Figure S1). The above-mentioned result suggested that P4HA2 can positively regulate OSCC cells’ capability of migrating and invading. Likewise, we used WB tests to analyze the molecular mechanism involved in depth. Western blotting test was used to measure MMP-2 and MMP-9 expression, and OSCC cell molecular mechanism (i.e., migration and invasion) was investigated. The results indicated that silencing P4HA2 down-regulated MMP-9 and MMP-2 expression, while P4HA2 overexpression promoted MMP-9 and MMP-2 expression (Fig. 4C). In conclusion, P4HA2 could affect OSCC cells’ capability of migrating and invading based on the regulation of MMP-9 and MMP-2 expression.

### P4HA2 regulates the PI3K/AKT pathway of OSCC cells in a collagen-dependent manner

**Figure 5:**
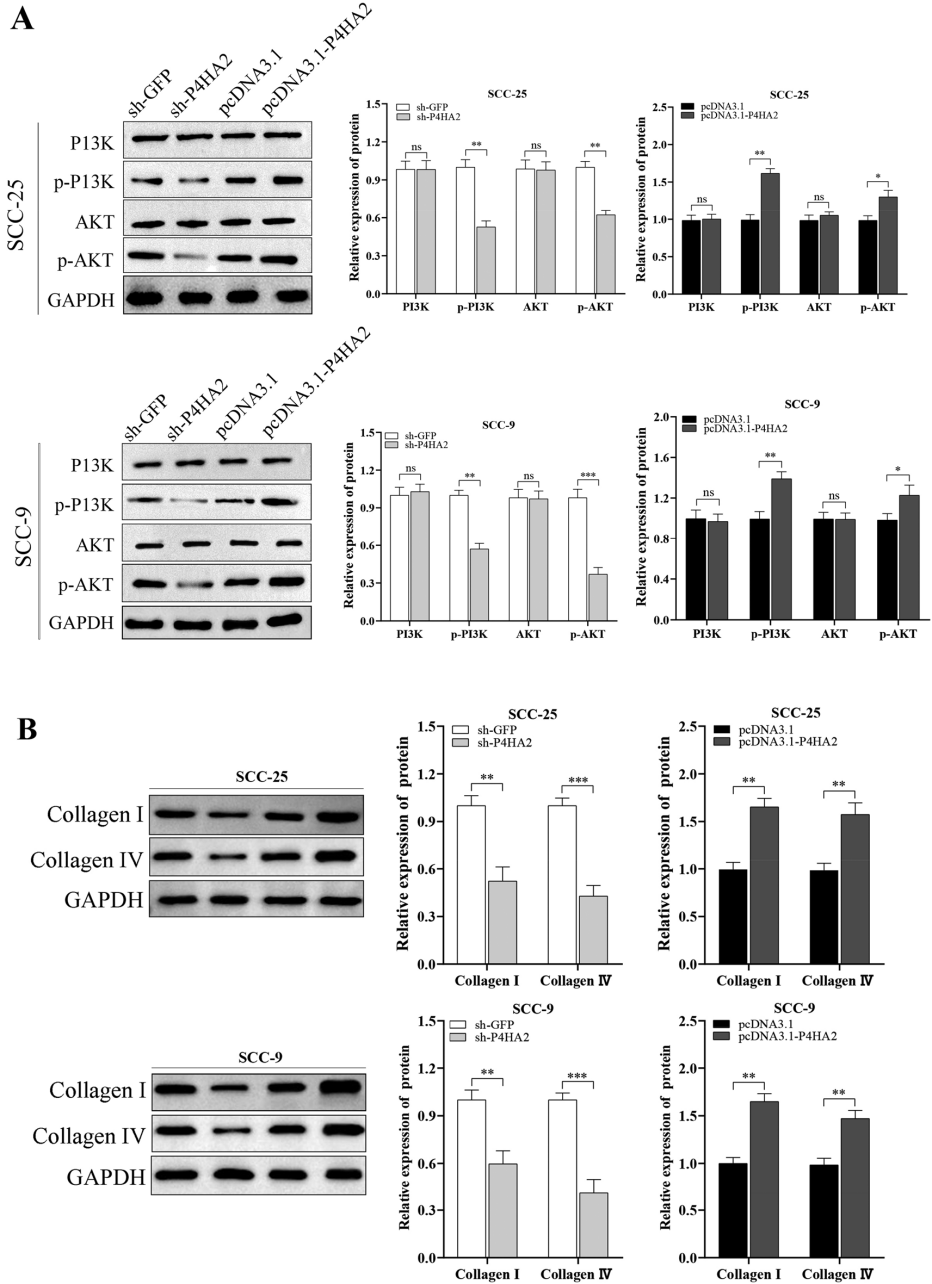

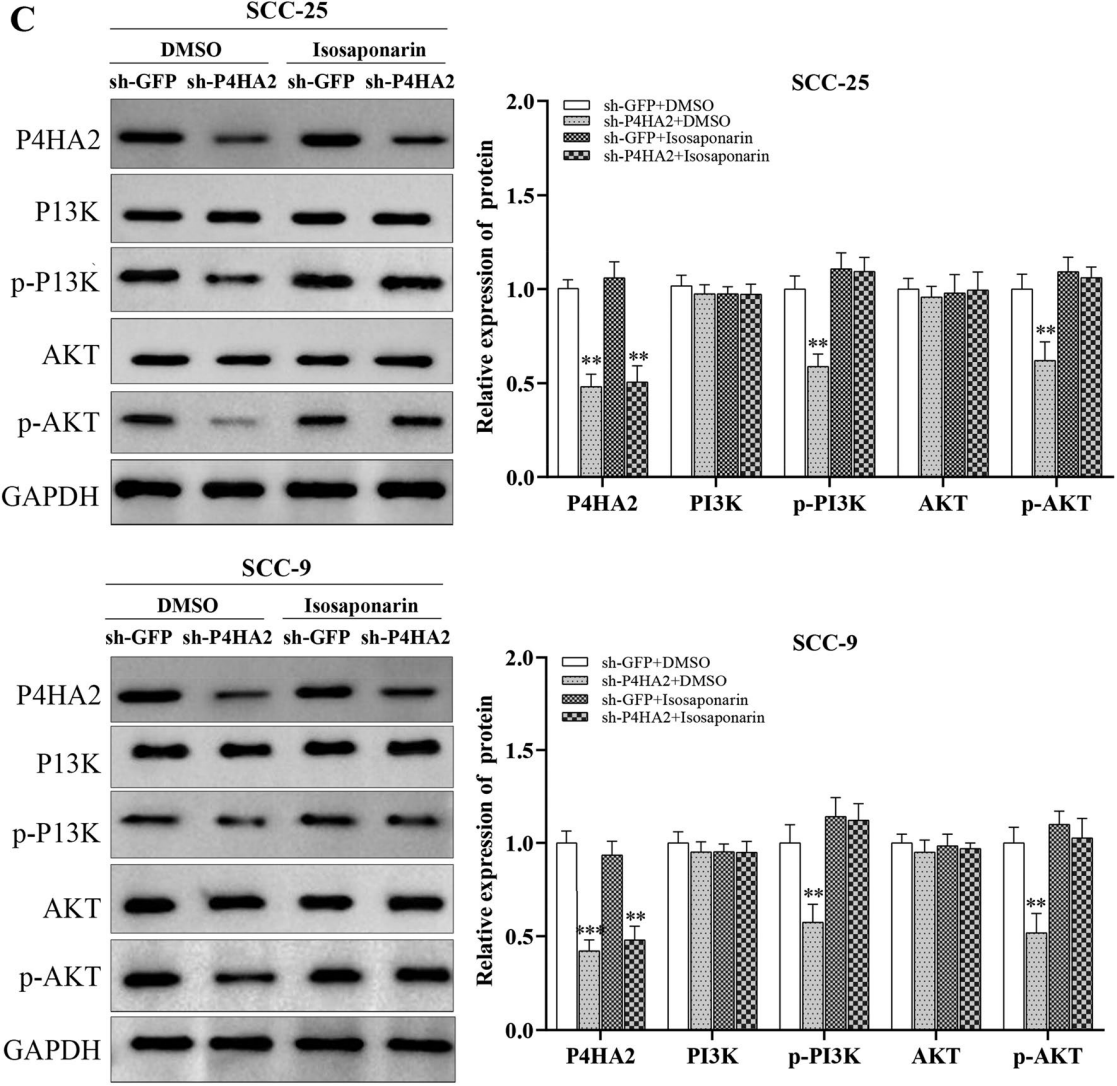
P4HA2 Regulates the PI3K/AKT Pathway of OSCC Cells in a Collagen-Dependent Manner. (**A**, **B**) A WB test was used to measure the protein expression of collagen I, collagen IV, PI3K, p-PI3K, AKT, and p-AKT in the sh-GFP, sh-P4HA2, pcDNA3.1-p4HA2, and pcDNA3.1 groups of SCC-25 and SCC-9. The data indicated that the expression levels of collagen I, collagen IV, p-PI3K, and p-AKT in OSCC cells were notably reduced in the sh-P4HA2 group, while those in the pcDNA3.1-P4HA2 group were notably higher than those in the sh-P4HA2 group. The expression levels of PI3K and AKT did not change notably between the sh-P4HA2 group and the pcDNA3.1-P4HA2 group. (**C**) The effect of a P4HA2 enzyme agonist on the PI3K/AKT signaling pathway was determined by a WB assay in OSCC cell lines. N = 3. *:*p* less than 0.05, **:*p* less than 0.01, ***:*p* less than 0.001, ****:*p* less than 0.0001.

The PI3K/AKT signaling pathway is well-known in tumor proliferation, invasion, and metastasis. To verify whether P4HA2 could regulate the PI3K/AKT pathway to play biological roles in OSCC cells, we evaluated collagen I, collagen IV, P13K, p-P13K, AKT and p-AKT expression in OSCC cells. And the results of WB tests indicated that collagen I, collagen IV, p-P13K and p-AKT expression in OSCC cells were notably reduced in the sh-p4HA2 group, while those in the pcDNA3.1-P4HA2 group were notably higher than those in the sh-p4HA2 group. Interestingly, PI3K and AKT expression did not change notably between the sh-p4HA2 group and the pcDNA3.1-P4HA2 group (Fig. 5A,B). In general, P4HA2 expedites OSCC cells’ proliferating, invading, and migrating processes based on the PI3K/AKT pathway. Moreover, to further test whether it is collagen that regulates the PI3K/AKT pathway, we utilized a P4HA2 enzyme agonist, Isosaponarin, to promote the synthesis of collagen in OSCC cells. Similar results were observed: both phosphorylated PI3K and AKT expressions, not the total protein expressions, were significantly increased by upregulating the biological function of the hydroxylase, implying that P4HA2 regulates PI3K/AKT signaling in a collagen-dependent manner (Fig. 5C).

### P4HA2 adjusts OSCC tumors growth in xenograft model

**Figure 6:**
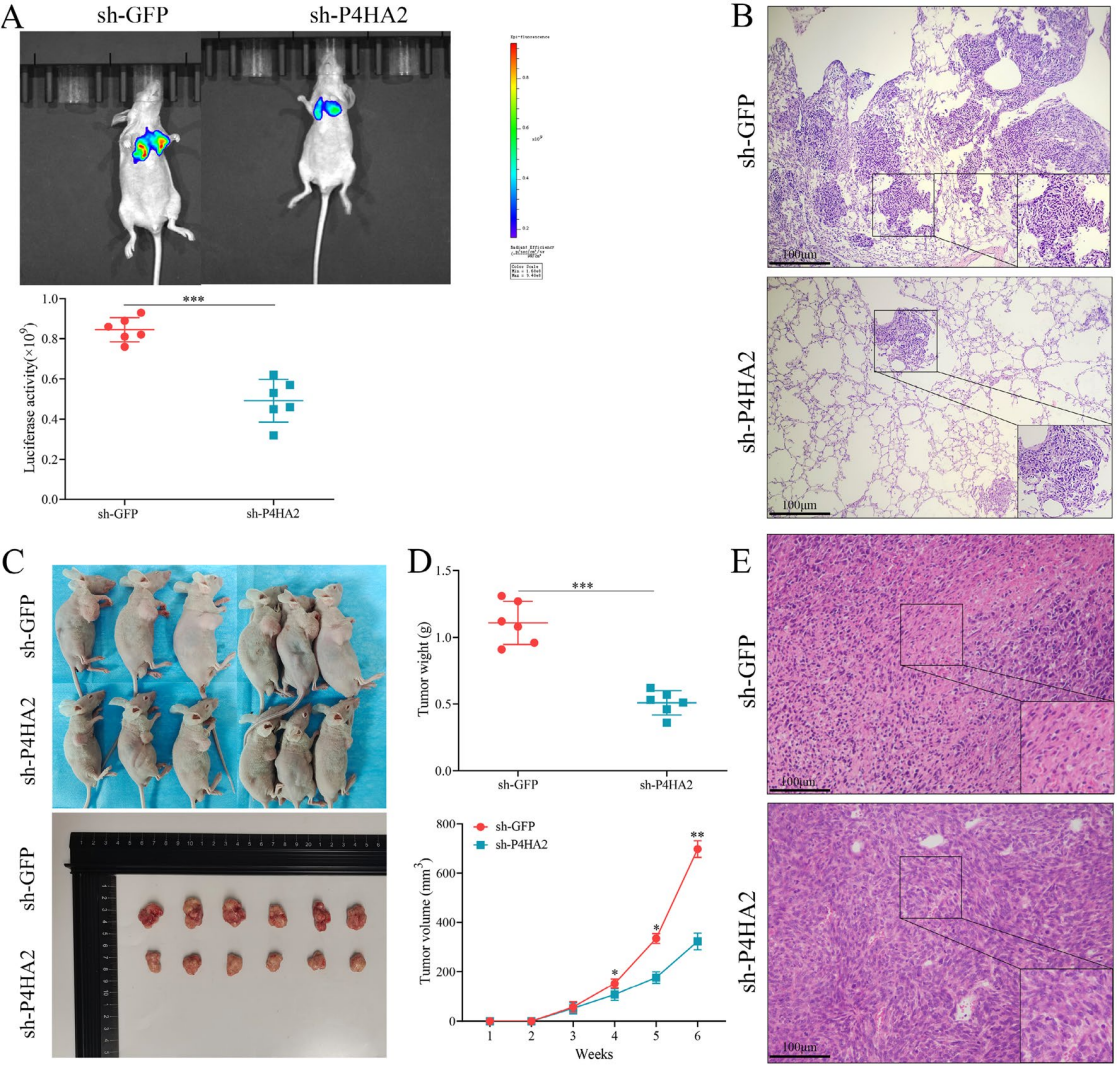
Down-regulated P4HA2 expression reduced OSCC cell proliferation and migration in vivo. (**A**) Based on the in vivo fluorescence imaging system, the Pulmonary metastasis model was identified for the evaluation of the migration capacity; the fluorescence intensity was analyzed. Next, the lungs of mice were isolated. (**B**) H&E staining was applied for the analysis of mice lungs' pulmonary metastasis cells (×100). (**C**) Effects of lentivirus infection on tumorigenicity of cells in the respective group. (**D**) The respective group material of body weight and tumor volume of mice. (**E**) H&E staining was used to indicate the pulmonary metastasis cells of mice lungs and the nucleus atypia of tumors. N = 6. *:*p* less than 0.05, **:*p* less than 0.01, ***:*p* less than 0.001, ****:*p* less than 0.0001.

We injected sh-P4HA2 and sh-GFP cells into the BALB/c nude mice for subcutaneous tumor formation to further explore the role of P4HA2 in OSCC growth. At the same time, the same two groups of cells were injected into other nude mice through the tail vein. The nude mice were sacrificed 6 weeks later, and the subcutaneous tumors and lung tissues were removed. The experiment indicated that the average body weight of the sh-P4HA2 group mice was lighter than that of the sh-GFP group. The tumor volume of the sh-P4HA2 group was notably smaller than that of the sh-GFP group. There were fewer lymph nodes in the lungs of sh-P4HA2 mice than in sh-GFP mice. Furthermore, the data of H&E staining indicated that the sh-P4HA2 group had a lower nucleus atypia degree. The above-described results suggested that P4HA2 can facilitate the occurrence and development of OSCC in vivo (Fig. 6).

## Discussion

OSCC is the most common type of oral cancer. Its rapid growth, strong invasiveness, and high rate of neck lymph node metastasis are significant reasons for the high mortality rate among patients^16^. Therefore, it is urgent to explore the molecular mechanisms affecting the proliferation, invasion, and metastasis of OSCC, and to identify biomarkers that can accurately predict prognosis and prevent recurrence, progression, and metastasis.

P4HA2 has been reported as a crucial enzyme that plays a significant role in cells' collagen synthesis and mature collagen formation^17^. The extracellular matrix (ECM) provides the physical framework for cells and initiates key cellular events such as survival, differentiation, proliferation, migration, and adhesion^18^. Mature collagen serves as a scaffold for the ECM and is the main component of it^19^. Additionally, it provides support for cells in spatial structures and acts as an anchor point for cell mechanical sensing^20^. Thus, P4HA2 is significantly correlated with cell migration, proliferation, differentiation, and other biological behaviors, playing a vital role in these processes. Furthermore, numerous signaling pathways within its structure regulate cell behavior^21^. Existing research has suggested that P4HA2 plays a role in many biological processes, including collagen maturation, epithelial-mesenchymal transition, and the deposition of amyloid β in Alzheimer's disease, and is vital in promoting tumor progression^22–24^. In this study, the results indicated that P4HA2 expression in OSCC tissues was significantly higher than in adjacent tissues, and P4HA2 expression in cancer tissues showed a negative correlation with the degree of differentiation in OSCC tissues. Moreover, high P4HA2 expression is positively correlated with high stage, high grade, and high lymph node metastasis rate in OSCC. The survival time of patients with high P4HA2 expression was also shorter than that of those with low P4HA2 expression. Multivariate analysis indicated that high P4HA2 expression is an independent poor prognostic factor for OS. Additionally, expression levels of both type I and type IV collagen were significantly altered—decreased and increased, respectively—after P4HA2 was knocked down and overexpressed, and both collagen types were positively correlated with P4HA2 expression. These results suggest that P4HA2 is critical to the occurrence and development of OSCC and shows a close correlation with the survival and prognosis of OSCC patients, possibly relating to the biological function of collagen.

Matrix metalloproteinase (MMP) is a highly homologous Zn^2+^ endopeptidase secreted by tumor and stromal cells. MMP-2 and MMP-9, members of the matrix metalloproteinase family, play a vital role in various tumors and are considered key regulators of cancer progression^25^. They can affect cancer migration and invasion by regulating the proteolysis of the vascular basement membrane and opening the metastasis pathway for endothelial cells, which is critical to the invasion and metastasis of breast cancer, pancreatic cancer, and oral squamous cell carcinoma^26–29^. In this study, the data indicated that cell migration and invasion were suppressed after the downregulation of P4HA2 expression. However, overexpression of P4HA2 had the opposite effect. This phenomenon was also observed at the expression levels of MMP-2 and MMP-9. These experimental results suggest that P4HA2 affects the migration and invasion of OSCC cells through the regulation of MMP-9 and MMP-2 expression. However, it is necessary to further clarify how P4HA2 affects MMP-2 and MMP-9 and to explore the specific molecular mechanisms by which P4HA2 regulates these enzymes.

In this study, we utilized the Gene Ontology (GO) database to analyze the potential mechanisms of P4HA2. The results indicated that P4HA2 was not only significantly enriched in ECM-receptor interactions but also in the PI3K/AKT signaling pathway in head and neck squamous cell cancer. PI3K is a catalytically active intracellular signaling protein that can adjust the phosphorylation level of downstream AKT, thus constituting the PI3K/AKT signaling pathway. This pathway plays a crucial role in controlling cell proliferation, growth, and survival and is activated in various cancers^30–33^. Phosphorylated PI3K and AKT may contribute to the biological behavior of cancer cells, such as proliferation, migration, and invasion. Studies have shown that the PI3K/AKT signaling pathway is abnormally activated in liver cancer, gastric cancer, breast cancer, oral squamous cell carcinoma, and other cancers, and participates in the development and progression of tumors. Additionally, it has been shown that changes in the conformation of ECM could lead to changes in the ambient pressure of cells, which activate transmembrane proteins such as integrins, phosphorylate intracellular focal adhesion kinase 2 (FAK2), activate proto-oncogene sarcoma receptor coactivator (Src), and eventually activate the PI3K/AKT signaling pathway to regulate the behavior of tumor cells^34^. The expression of PI3K/AKT signaling pathway-associated proteins was down-regulated after P4HA2 expression was inhibited, while P4HA2 pathway-associated protein expression was up-regulated after the promotion of P4HA2 expression. These results suggest that P4HA2 could regulate the PI3K/AKT signaling pathway in OSCC cells. Furthermore, the potential link between P4HA2 and the PI3K/AKT signaling pathway was explored by a rescue assay using a collagen synthesis agonist. These results suggest that P4HA2 can regulate PI3K/AKT expression at the protein level, but not at the mRNA level. Additional results showed that PI3K/AKT could be upregulated by promoting the collagen hydroxylase activity of P4HA2, supporting our proposal that PI3K/AKT expression activation is via a P4HA2 enzyme-mediated protein–protein interaction rather than direct transcriptional activation. Therefore, the above results indicated that MMPs are the critical downstream effectors of the P4HA2-collagen-PI3K/AKT axis. The underlying mechanism may be regulating the collagen-dependent PI3K/AKT signaling pathway.

Abnormal tumor proliferation arising from cell cycle disorder is a key factor in tumorigenesis. As a key cell cycle regulatory protein, Cyclin D1 is located on chromosome 11q13, which can drive cells from the G1/G0 phase to the S phase^35^. Existing research has suggested that Cyclin D1 is frequently dysregulated in cancer and could be a vital biomarker for cancer phenotype and disease progression^36^. Numerous signaling pathways (e.g., Ras/MEK/ERK, PI3K/AKT, and Ral) are capable of regulating Cyclin D1 expression. The protein degradation of Cyclin D1 is regulated by glycogen synthase kinase 3β (GSK-3β). After PI3K activates AKT, AKT phosphorylates GSK-3β to inactivate it, which can up-regulate Cyclin D1 protein expression^37^. Survivin, the smallest member of the inhibitor of apoptosis protein family, is a 16.5 kDa protein encoded by the BIRC5 gene on chromosome 17^38^. Additionally, it is critical in regulating cell division and blocking caspase activation to inhibit apoptosis. Compared with normal tissues, survivin is overexpressed in most human cancers, and its abnormal expression is correlated with tumor proliferation, apoptosis, angiogenesis, treatment resistance, and poor prognosis^39^. It has been reported that the survivin gene is not expressed in normal human oral keratinocytes but is highly expressed in four OSCC cell lines. Moreover, existing research has suggested that the abnormal expression of survivin in tumor cells is regulated by a variety of factors, including microRNA (miRNA), receptor tyrosine kinase (RTK), and their downstream signaling pathways (e.g., MAPK, PI3K/AKT, signal transducer, TAT3, and other pathways)^40^. In the experiments of this study, we found that Cyclin D1 and survivin protein expression was down-regulated after silencing P4HA2. Conversely, P4HA2 overexpression led to up-regulated Cyclin D1 and survivin expression. The above-mentioned results suggest that P4HA2 can play a certain role in the apoptosis and proliferation of OSCC cells through the regulation of Cyclin D1 and survivin via the PI3K/AKT signaling pathway.

## Conclusion

In summary, this study indicates that P4HA2 is highly expressed in OSCC tissues, and its expression level correlates with tumor stage, grade, degree of differentiation, lymph node metastasis, and survival time in OSCC patients. It may serve as a biomarker to determine the prognosis of OSCC patients and assist doctors in identifying high-risk patients, facilitating early, active, and personalized treatment post-surgery. Additionally, the underlying molecular mechanism appears to influence OSCC cells' invasion, migration, and proliferation by positively regulating the collagen-dependent PI3K/AKT signaling pathway. In subsequent studies, we will further investigate the specific molecular interactions between P4HA2 and the PI3K/AKT signaling pathway in OSCC cells and analyze P4HA2's role and mechanisms in OSCC in greater depth to propose novel ideas and methods for treating OSCC.

### Ethics approval and consent to participate

1. The research methods using in the experiment about human tissue samples were accordance with the guidelines of the Declaration of Helsinki. And the experiment was authorized by the Ethics and Humanities Committee of Qingdao Municipal Hospital (Shandong, China). All subjects and/or their legal guardians gave their informed consent.

2. All animal experiments were approved by the Ethics and Humanities Committee of Qingdao Municipal Hospital (Shandong, China) and performed according to the animal care and use guidelines of the National Institutes of Health, USA. Finally, the animal study has been reported in the manuscript in accordance with ARRIVE guidelines.

## Supplementary Information

Supplementary Information 1.

Supplementary Information 2.

Supplementary Figure 1.

## Data Availability

The datasets used and/or analyzed during the current study are available fromthe corresponding author on reasonable request.
