# Author Correction: P4HA2 promotes proliferation, invasion, and metastasis through regulation of the PI3K/AKT signaling pathway in oral squamous cell carcinoma

**DOI:** 10.1038/s41598-024-76811-1

**Published:** 2024-10-28

**Authors:** Zengpeng Chi, Qimin Wang, Xin Wang, Dagang Li, Lei Tong, Yu Shi, Fang Yang, Qingyuan Guo, Jiawei Zheng, Zhenggang Chen

**Affiliations:** 1 Department of Stomatology, Qingdao Huangdao District Central Hospital, Qingdao, 266555 China; 2 Department of Stomatology, Qingdao Hospital, University of Health and Rehabilitation Sciences(Qingdao Municipal Hospital), No.5 Donghai Middle Road, Qingdao, 266071 China; 3 Acupuncture and Tuina Department, Changchun University of Chinese Medicine, Changchun, 130117 Jilin China; 4 Department of Stomatology, Shenzhen‑Shanwei Central Hospital, Sun Yat-Sen University, Shanwei, 516699 China; 5 Department of Oromaxillofacial Head and Neck Oncology, College of Stomatology, Shanghai Ninth People’s Hospital, Shanghai Jiao Tong University School of Medicine, No.639, Manufacturing Bureau Road, Huangpu District, Shanghai, 200011 China; 6 Institute of Stomatology, Binzhou Medical University, Binzhou, 256600 China; 7 The Affiliated Yantai Stomatological Hospital, Binzhou Medical University, Binzhou, 264000 China

Correction to: *Scientific Reports* 10.1038/s41598-024-64264-5, published online 01 July 2024

The original version of this Article contained errors.

In the Materials and methods section, ‘Cell invasion test’.

“The bottom membrane of the chamber was examined under a microscope at 200× magnification, and 5 random fields were selected for photography.”

now reads:

“The bottom membrane of the chamber was examined under a microscope at 40× and 200× magnification, and 5 random fields were selected for photography. Representative 40× images are shown in Figure 4B and 200× images in the Supplementary Figure S1.”

Additionally, Supplementary Figure 1 showing representative 200 × images from the cell invasion test has now been included in the new “Supplementary Figure 1”.

Furthermore, in the Results section, ‘P4HA2 adjusts OSCC cells’ metastasis and invasion’.

“However, P4HA2 overexpression reversed the results (Fig. 4A, B).”

now reads:

“However, P4HA2 overexpression reversed the results (Fig. 4A, B and Supplementary Figure S1).”

Finally, the representative image corresponding to ‘SCC-9 sh-GFP’ in the bottom panel of Figure 4B was incorrect. The original Figure 4 and the accompanying legend appear below.

**Fig. 4:**
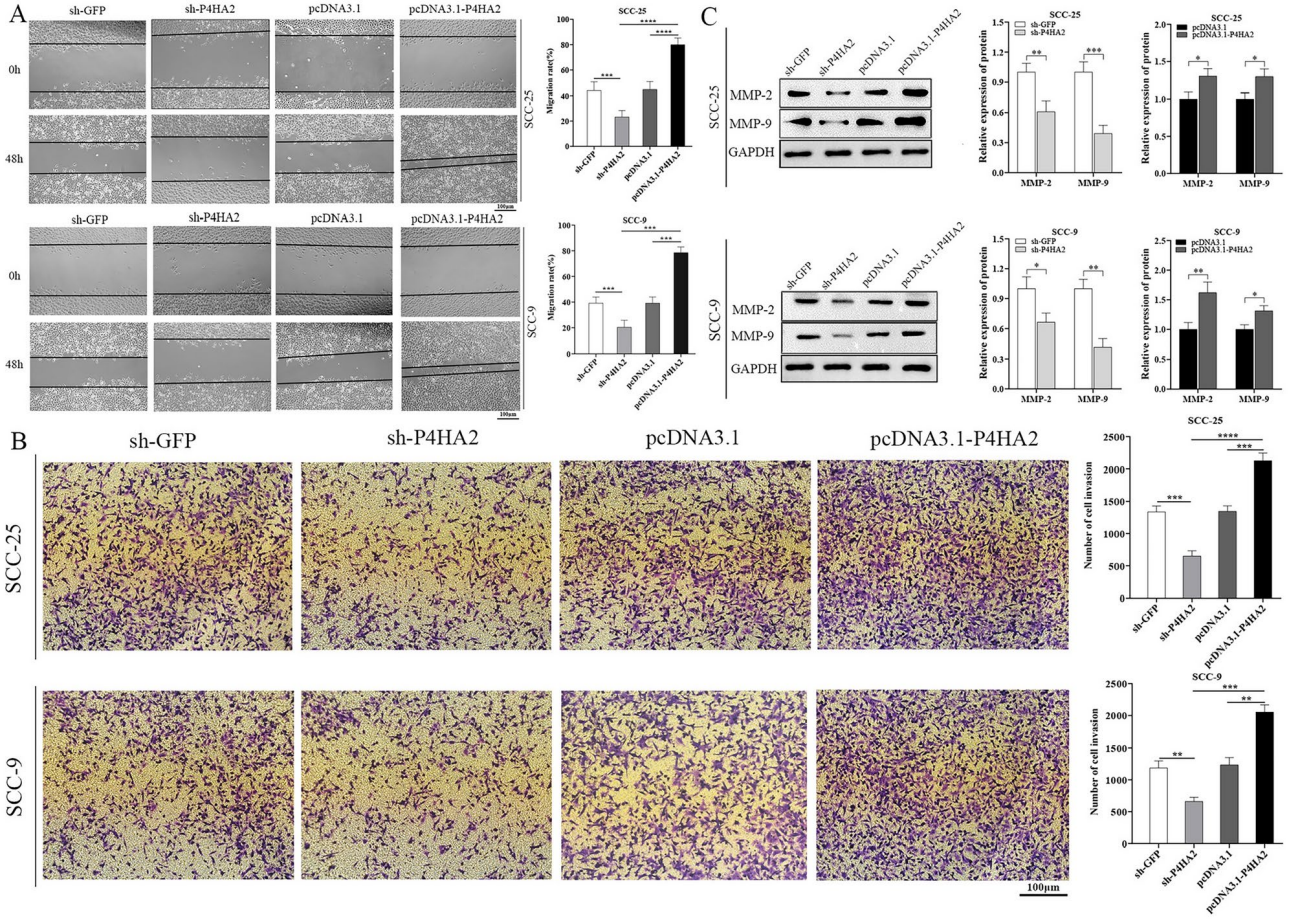
Influence of P4HA2 expression on OSCC cells’ metastasis and invasion. (**A**,**B**) Migration and invasion tests examined the cell migration ability in the sh-P4HA2, sh-GFP, pcDNA3.1-p4HA2, and pcDNA3.1 groups of SCC-25 and SCC-9, analyzed separately. The data indicated that P4HA2 could positively regulate the OSCC cells’ ability to migrate and invade. (**C**) A WB test was used to measure MMP-9 and MMP-2 expression in the sh-P4HA2, sh-GFP, pcDNA3.1-p4HA2, and pcDNA3.1 groups of SCC-25 and SCC-9. The data indicated that P4HA2 knockdown suppressed MMP-9 and MMP-2 expression. Furthermore, we found opposite results when P4HA2 was overexpressed. N = 3. *:*p* less than 0.05, **:*p* less than 0.01, ***:*p* less than 0.001, ****:*p* less than 0.0001..

The original Article has been corrected.

