# Supplementary material for: P4HA2 promotes proliferation, invasion, and metastasis through regulation of the PI3K/AKT signaling pathway in oral squamous cell carcinoma
Source: Sci Rep. 2024 Jul 1;14:15023. doi: 10.1038/s41598-024-64264-5 (PMC11217378; doi:10.1038/s41598-024-64264-5)
Supplement: Supplementary file 1 — Supplementary Information 1. [file 41598_2024_64264_MOESM1_ESM.docx]

**Fig.3A:**


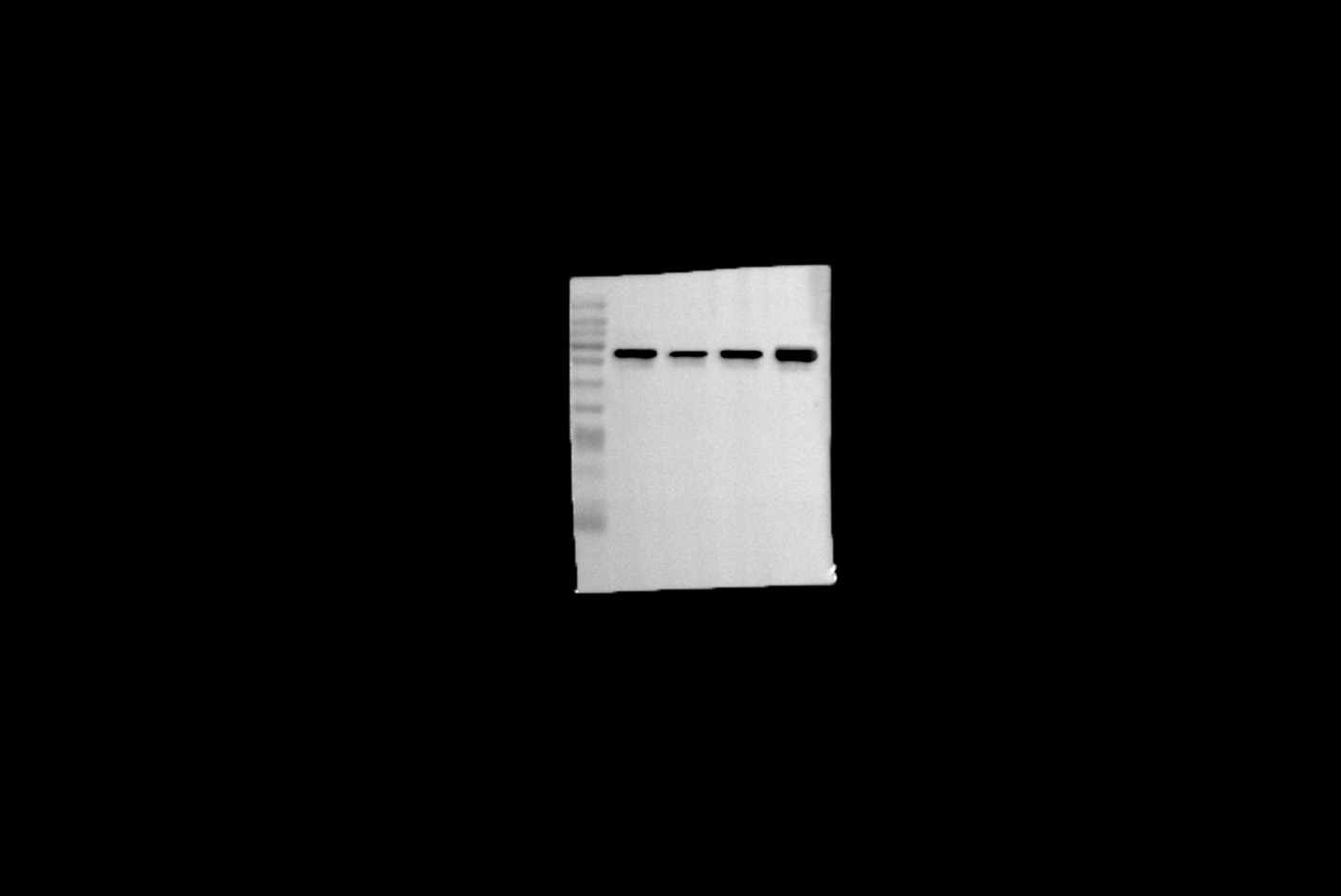

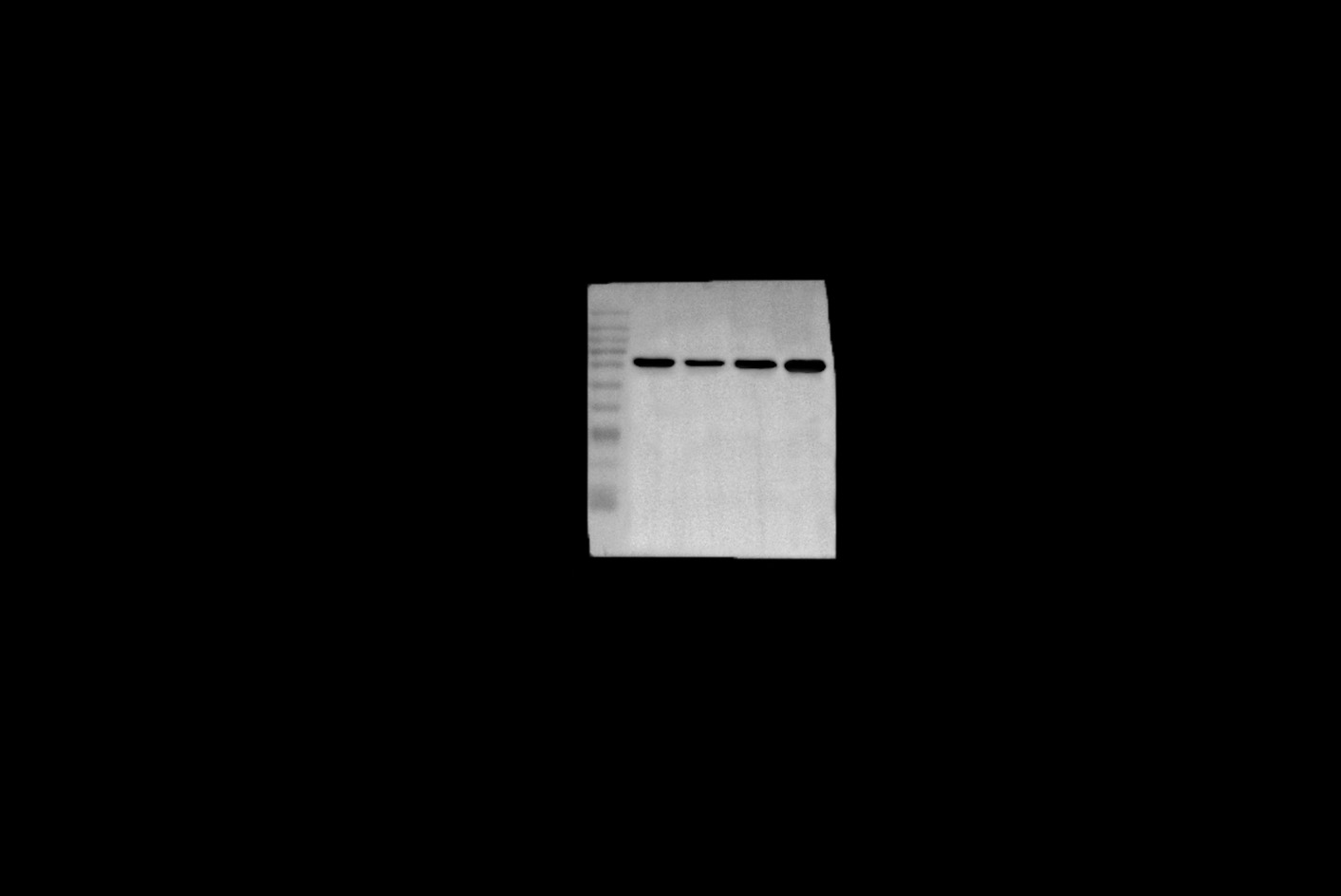


SCC-25 P4HA2 SCC-9 P4HA2


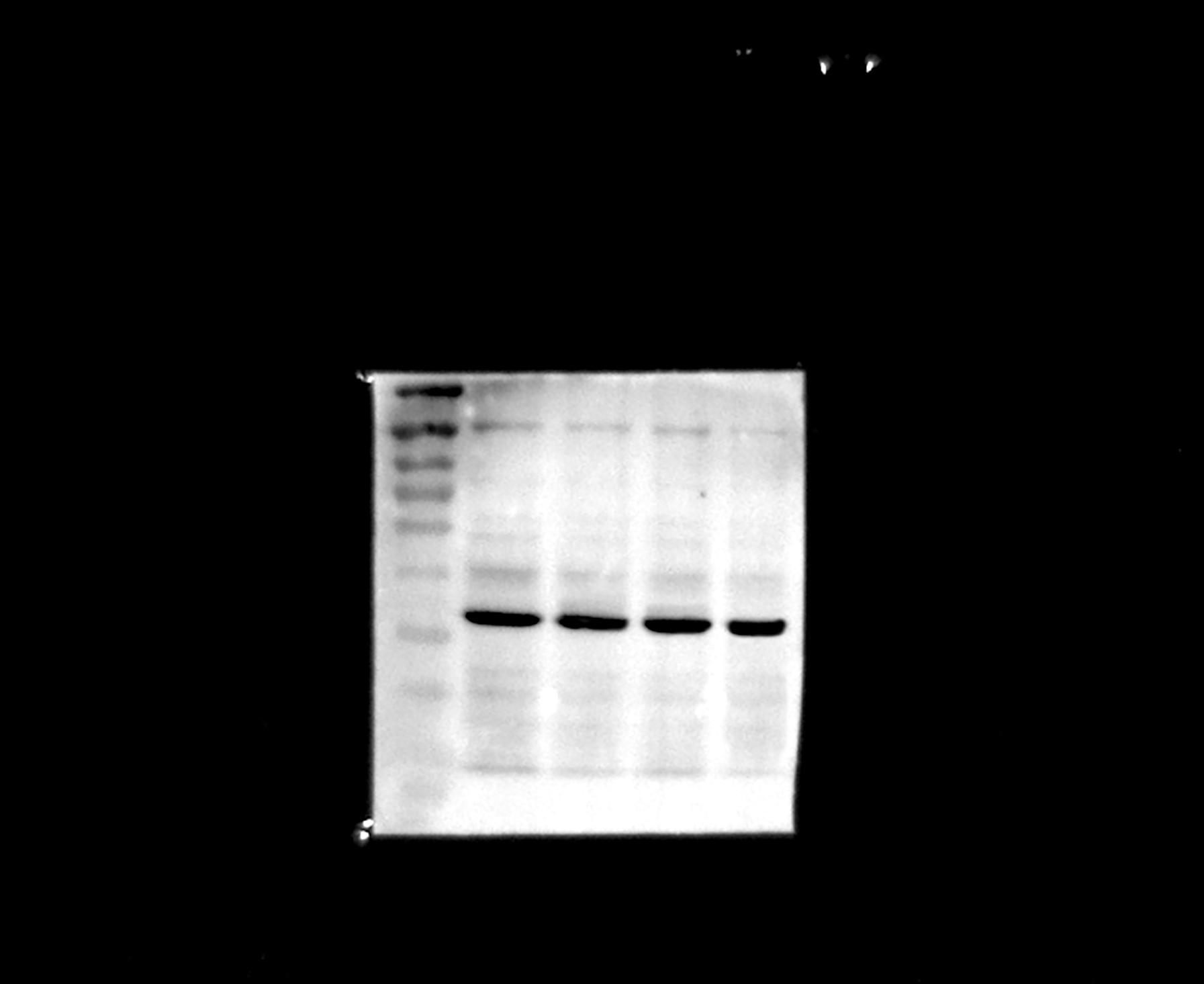

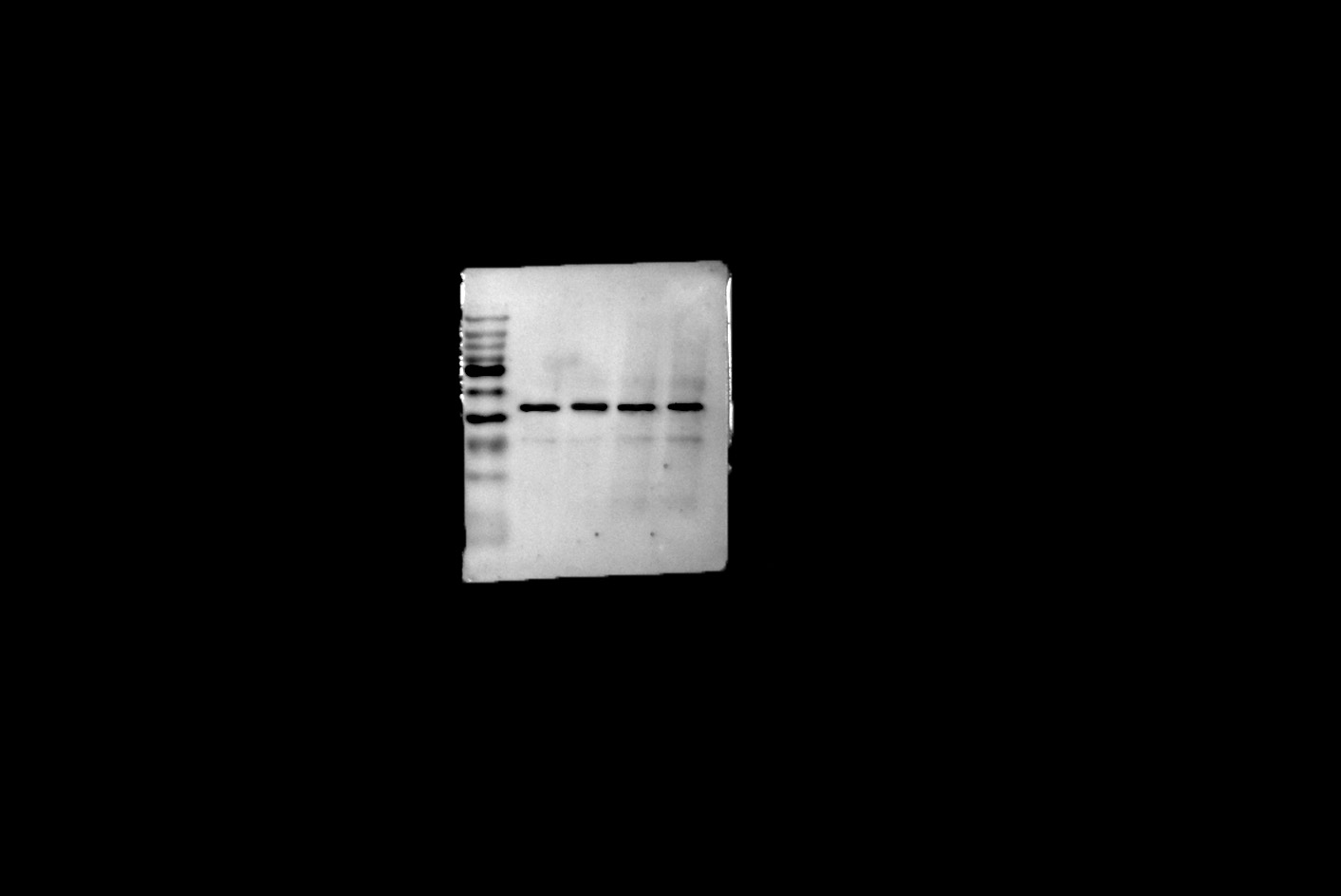


SCC-25 GAPDH SCC-9 GAPDH

**Fig.3E:**


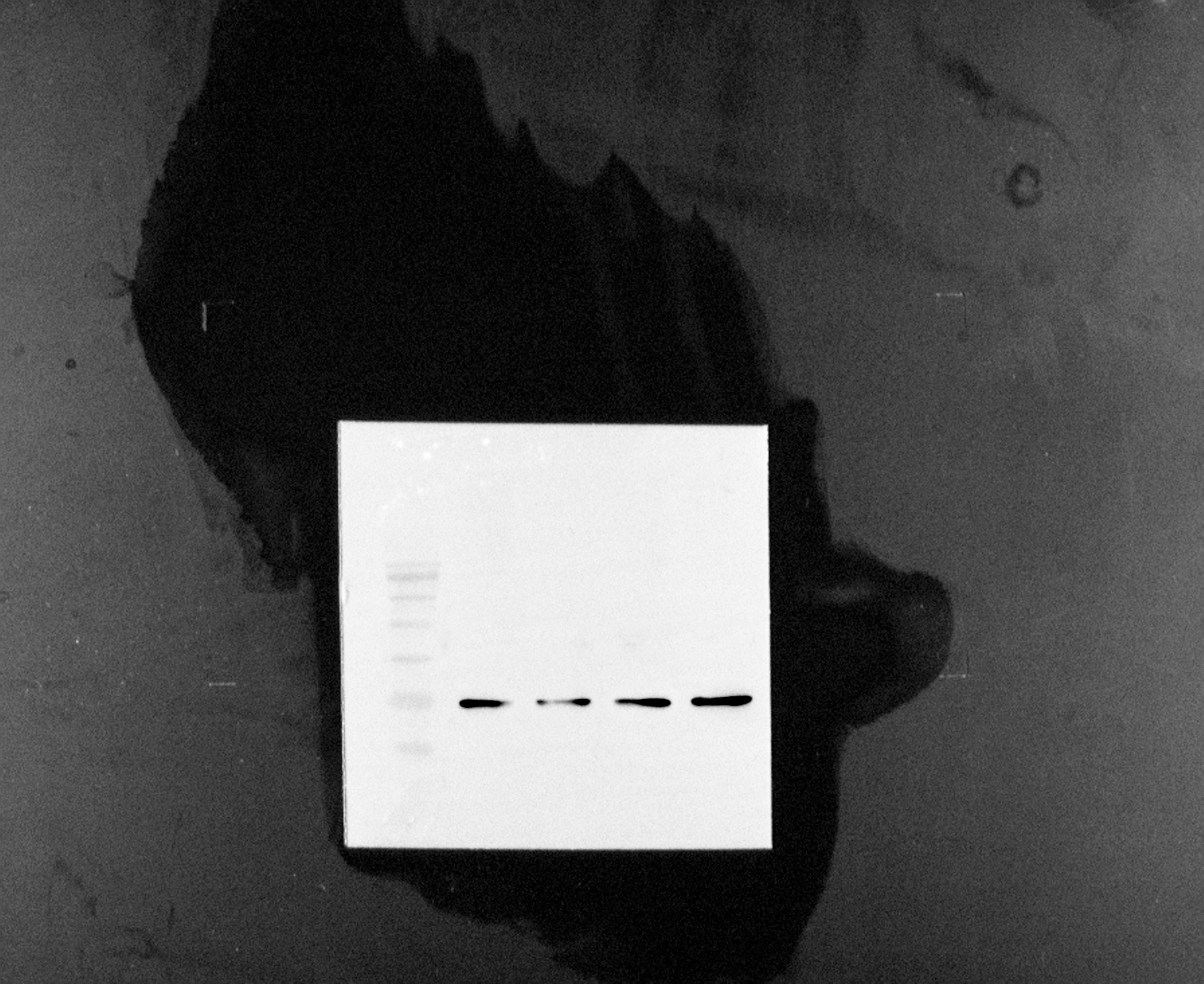

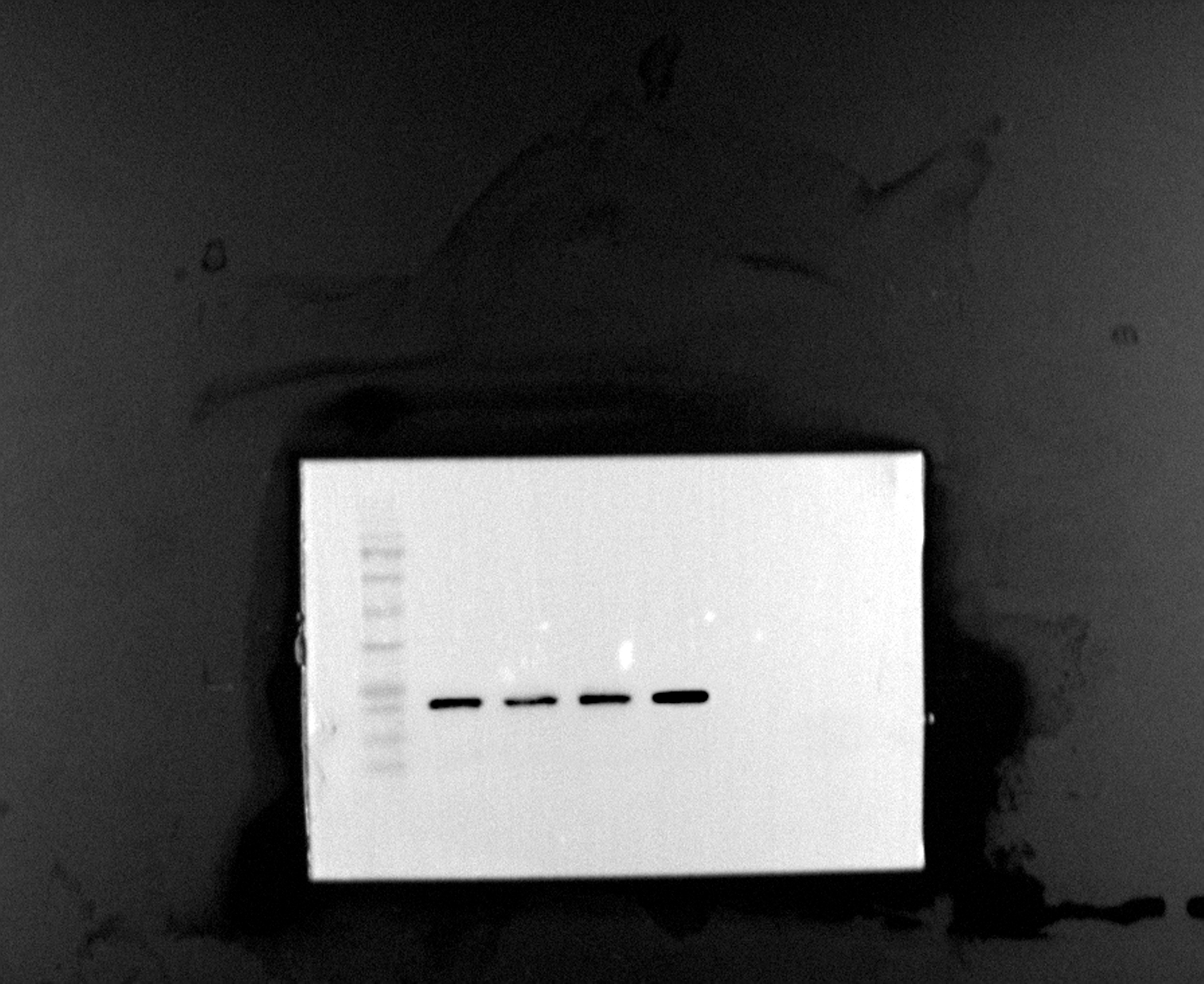


SCC-25 CyclinD1 SCC-9 CyclinD1


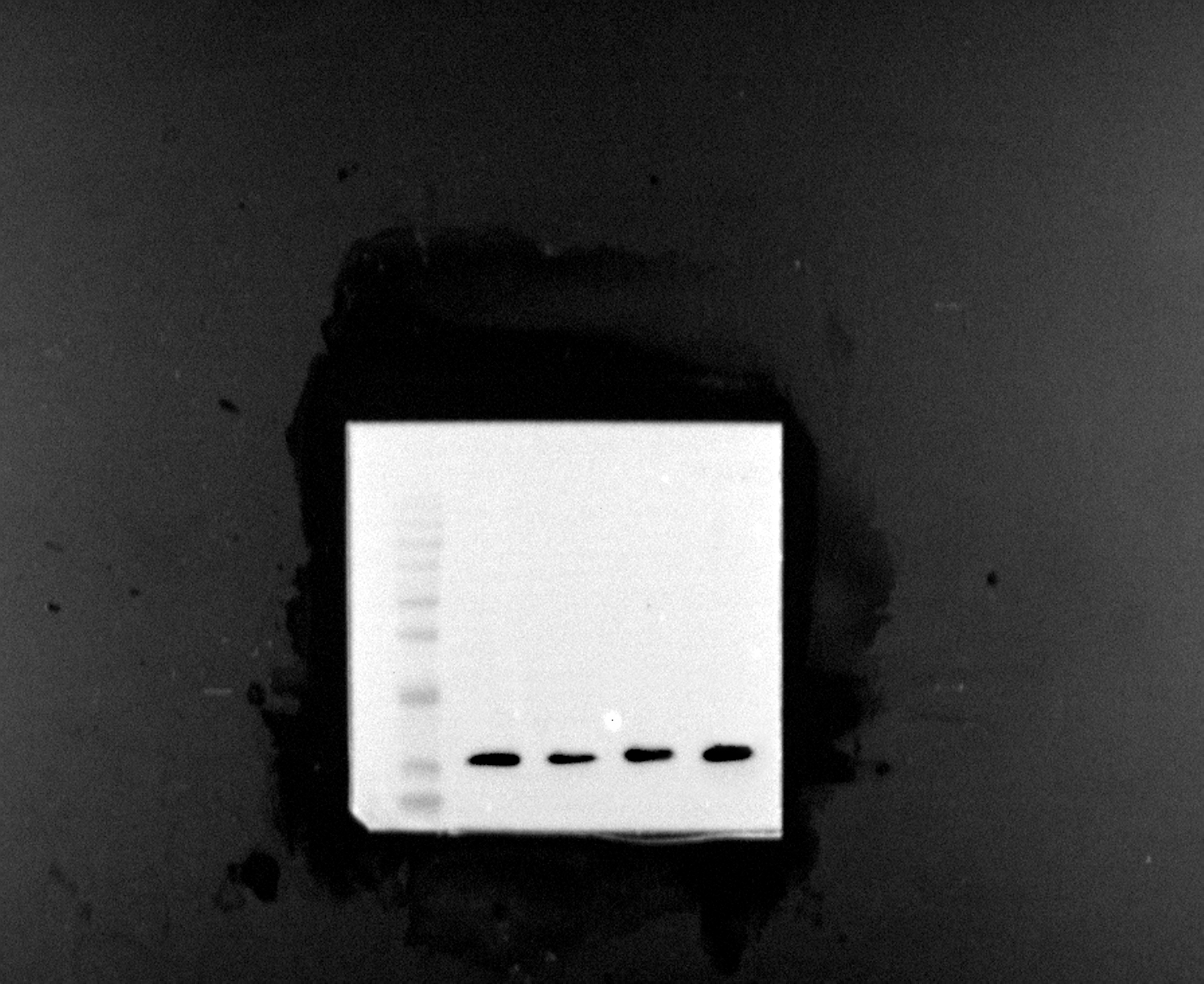

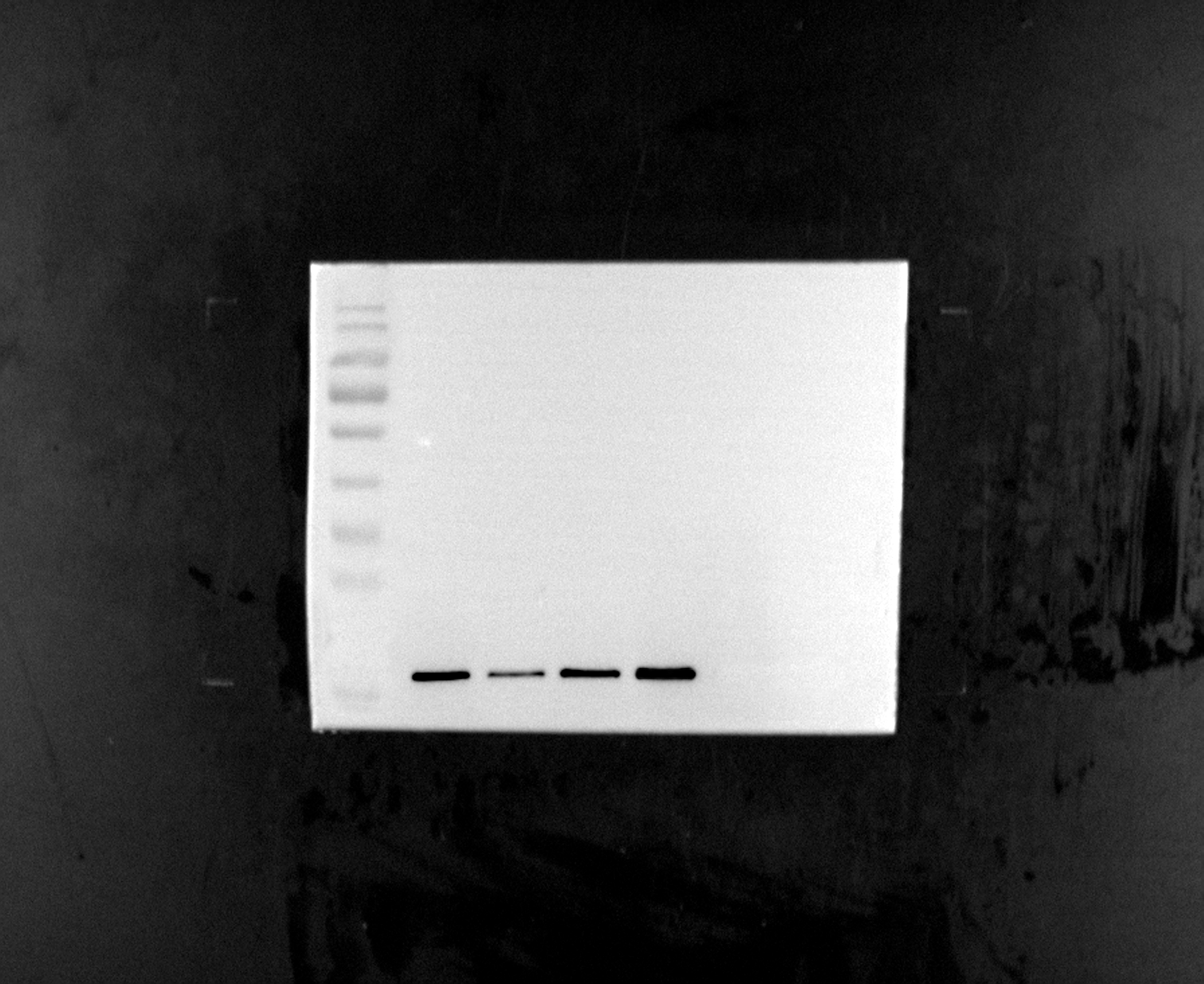


SCC-25 Survivin SCC-9 Survivin


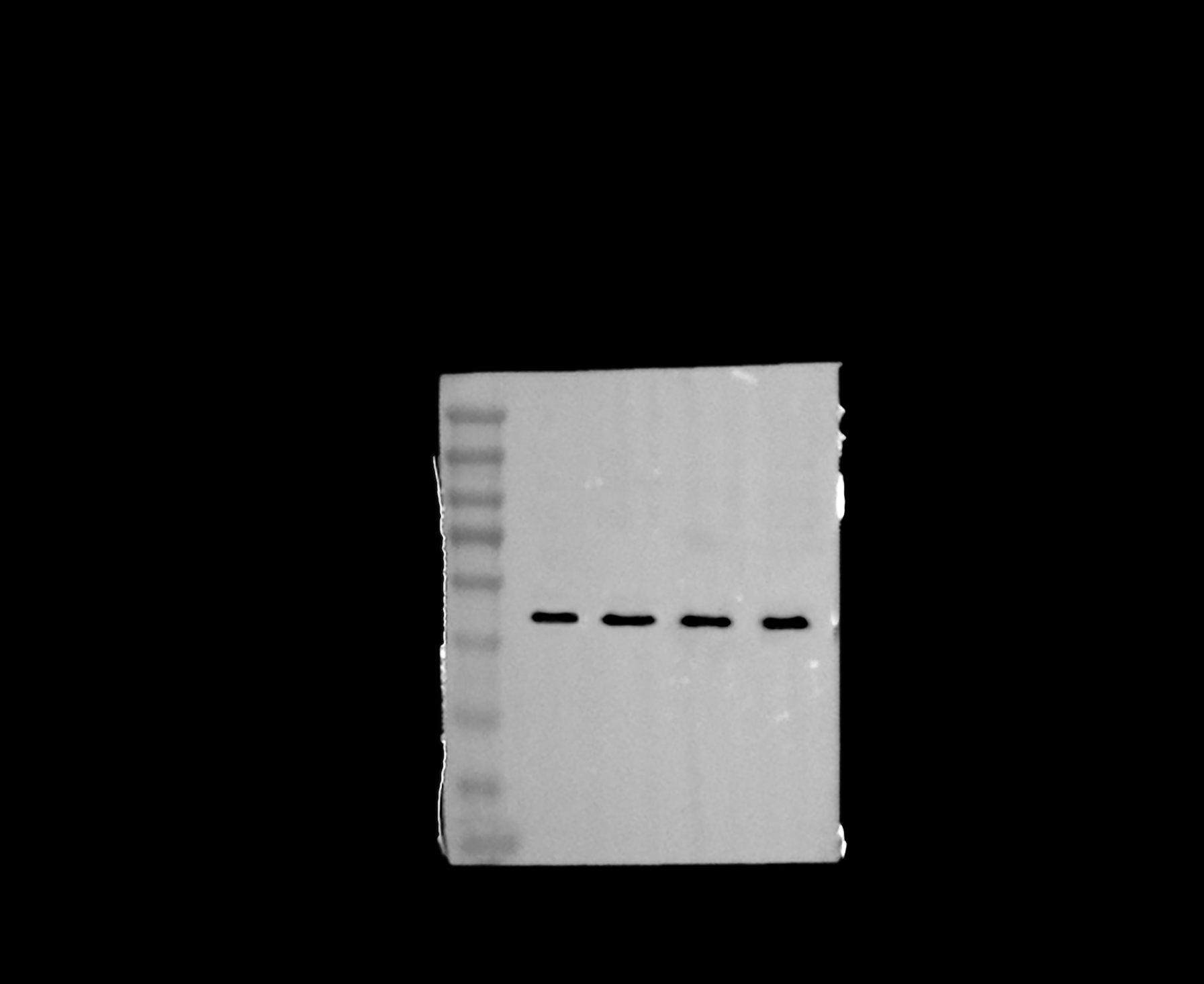

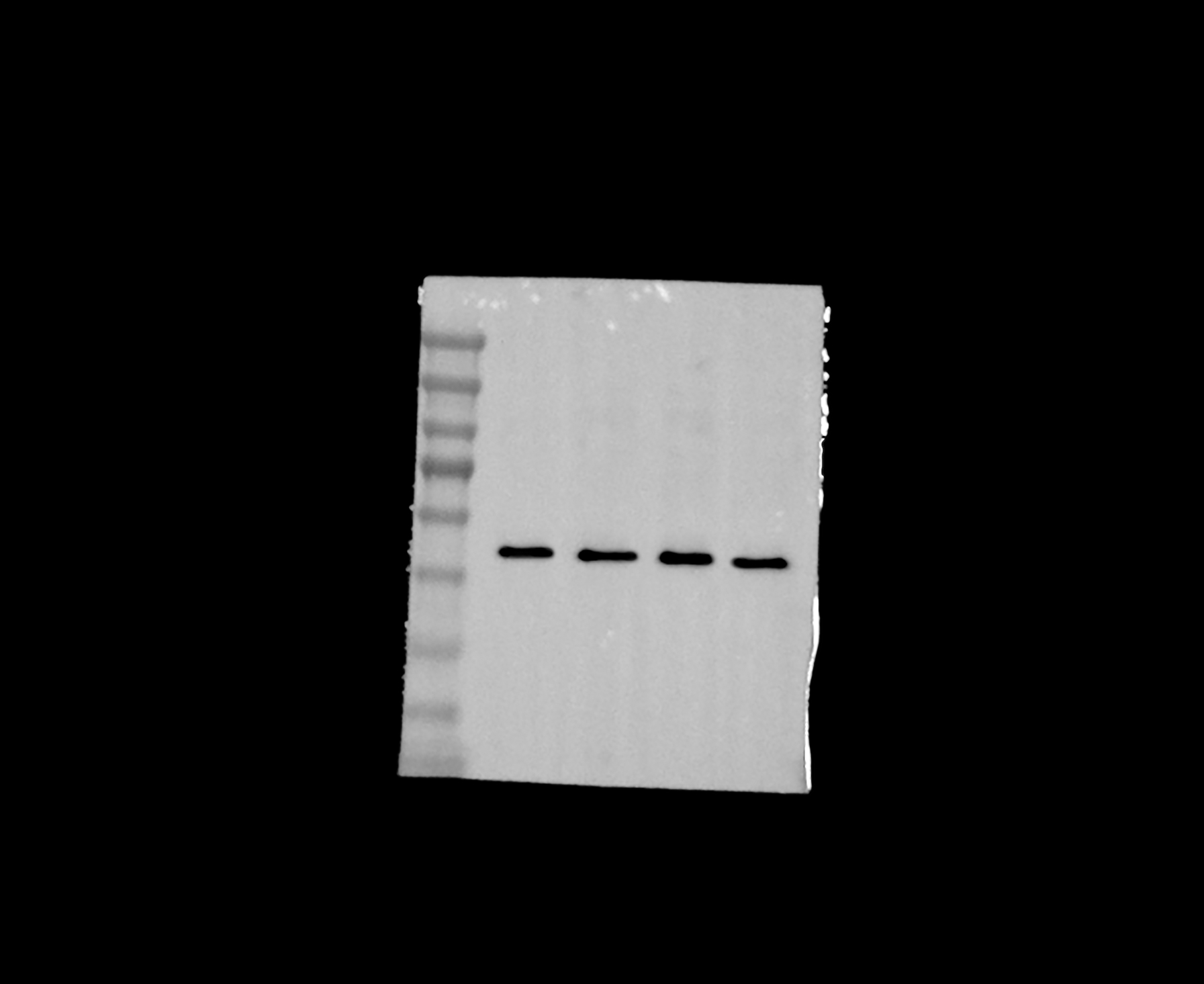


SCC-25 GAPDH SCC-9 GAPDH

**Fig.4C:**


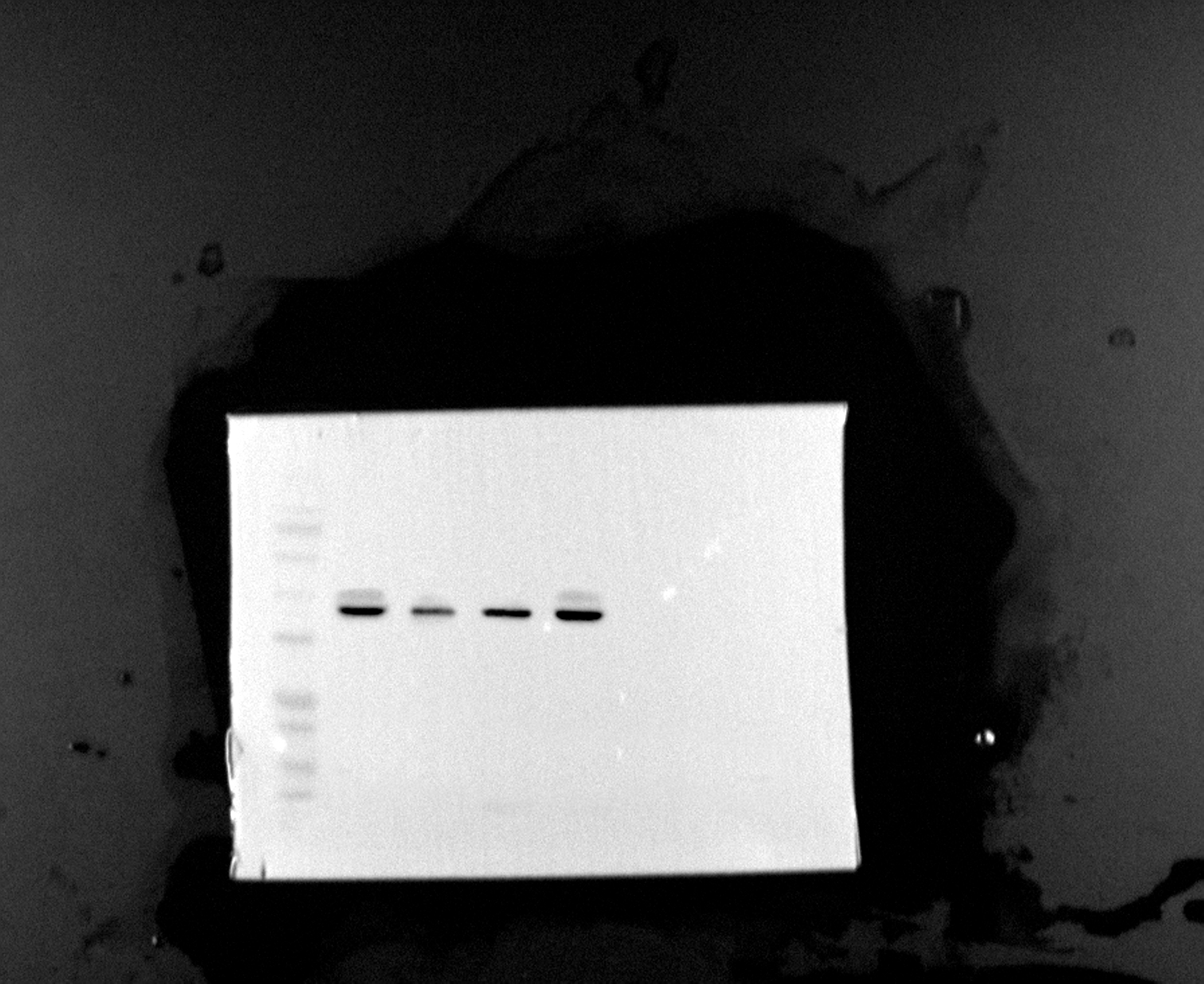

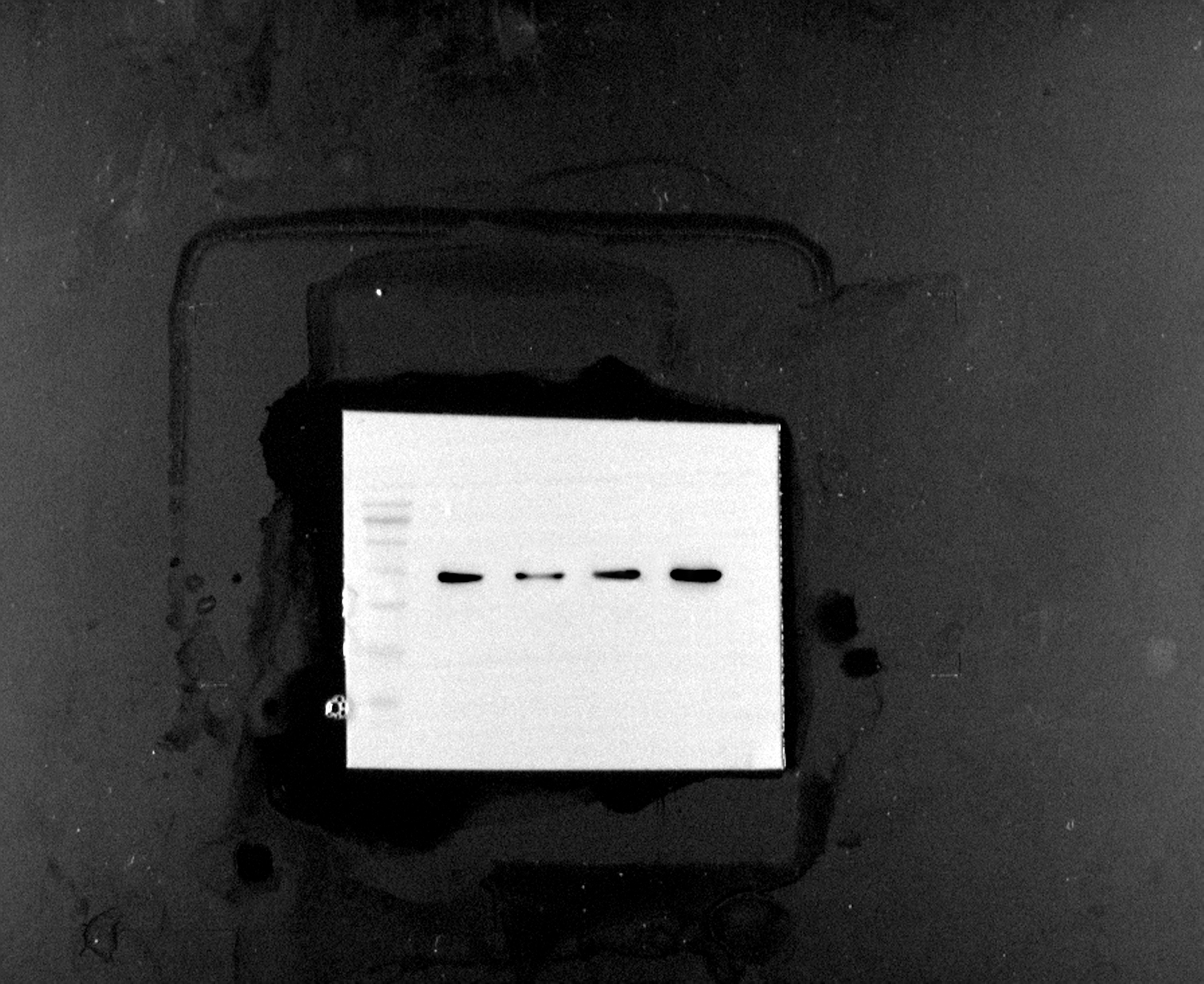


SCC-25 MMP-2 SCC-9 MMP-2


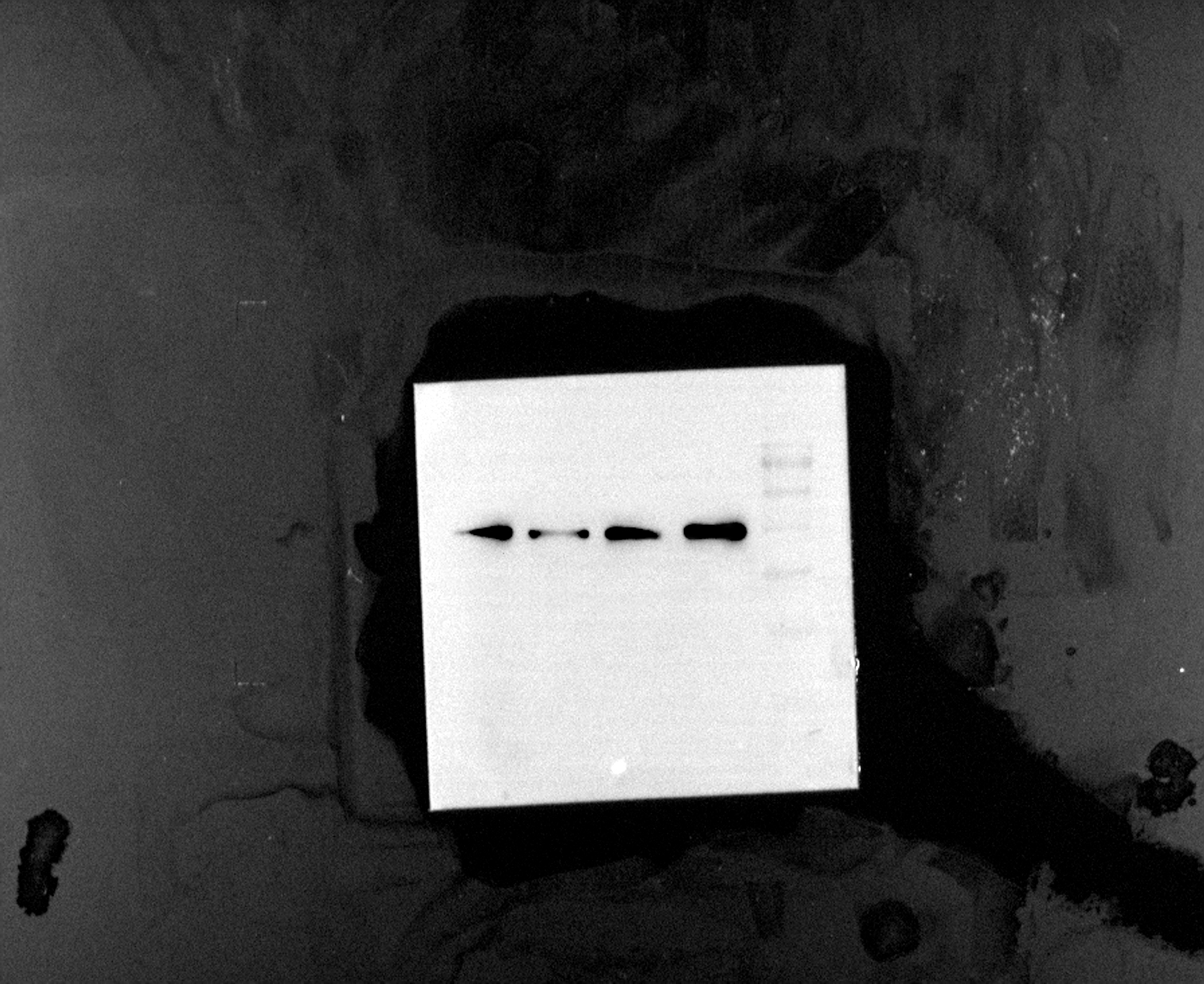

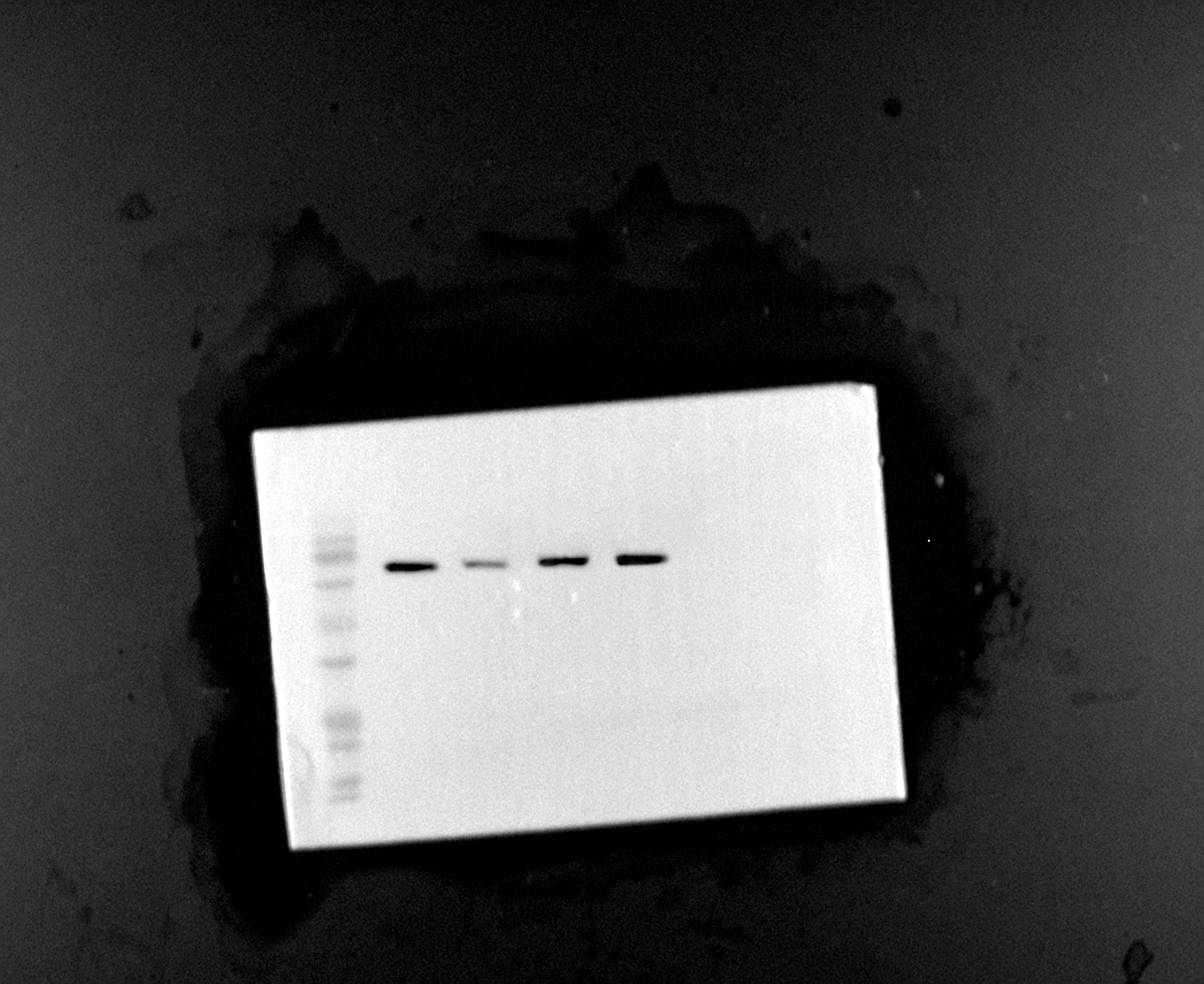


SCC-25 MMP-9 SCC-9 MMP-9


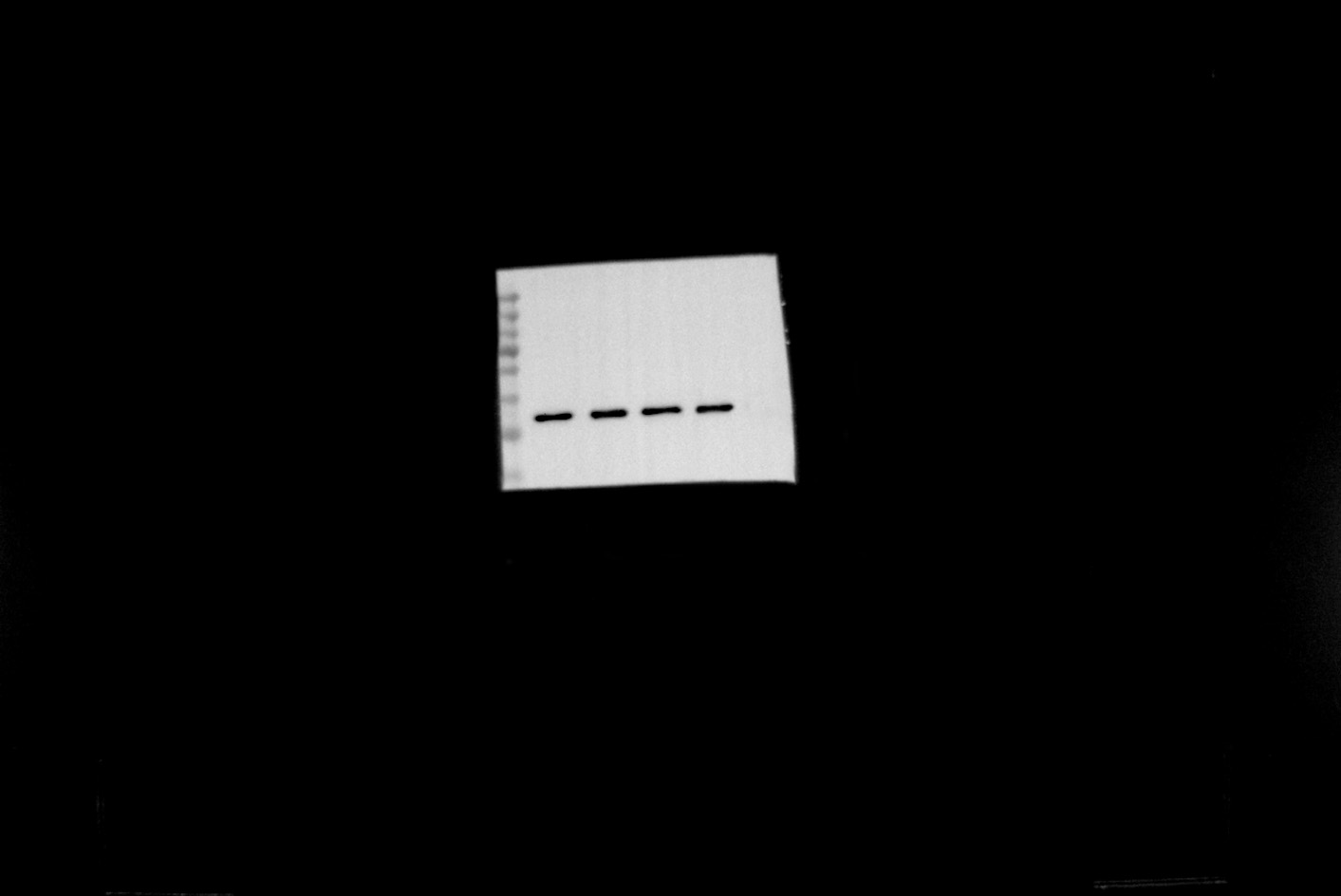

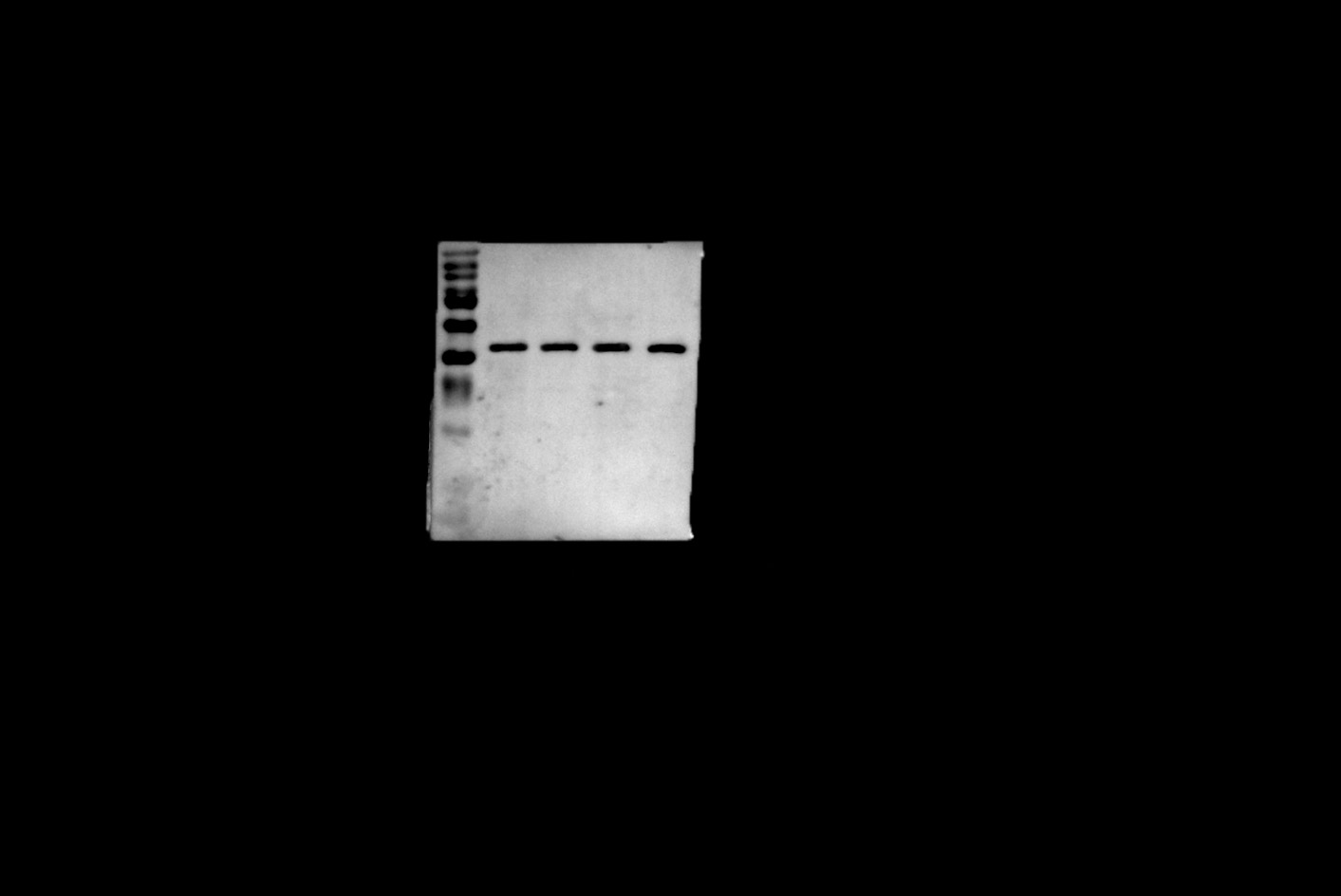


SCC-25 GAPDH SCC-9 GAPDH

**Fig.5A:**


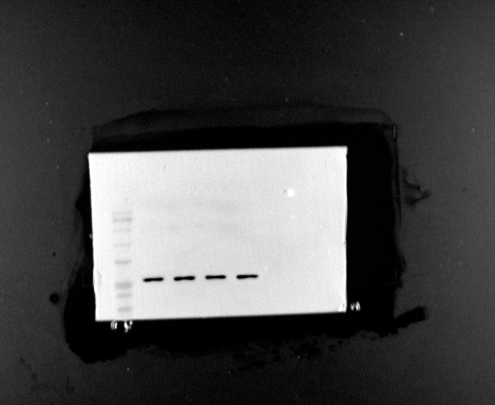


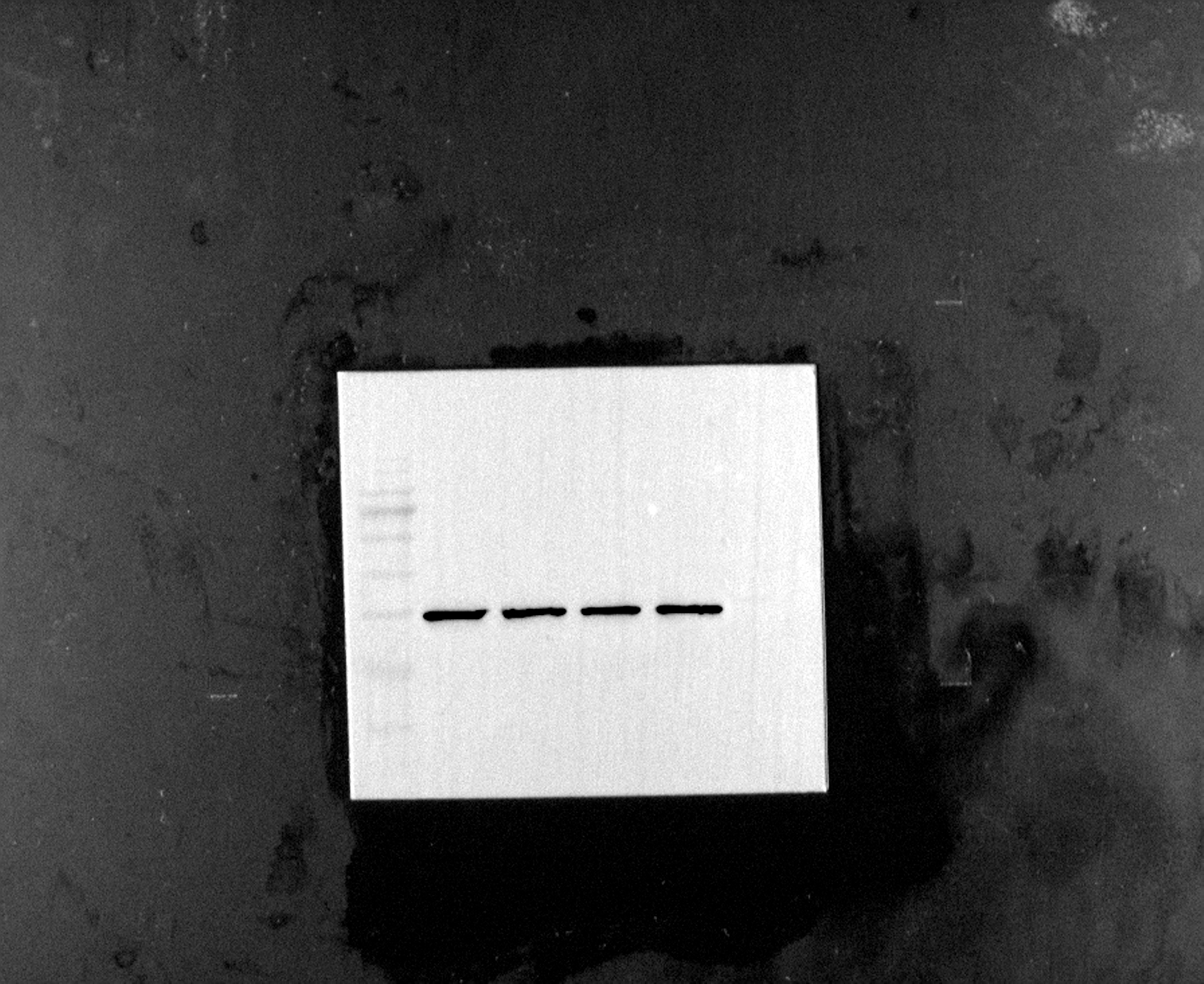


SCC-25 PI3K SCC-9 PI3K


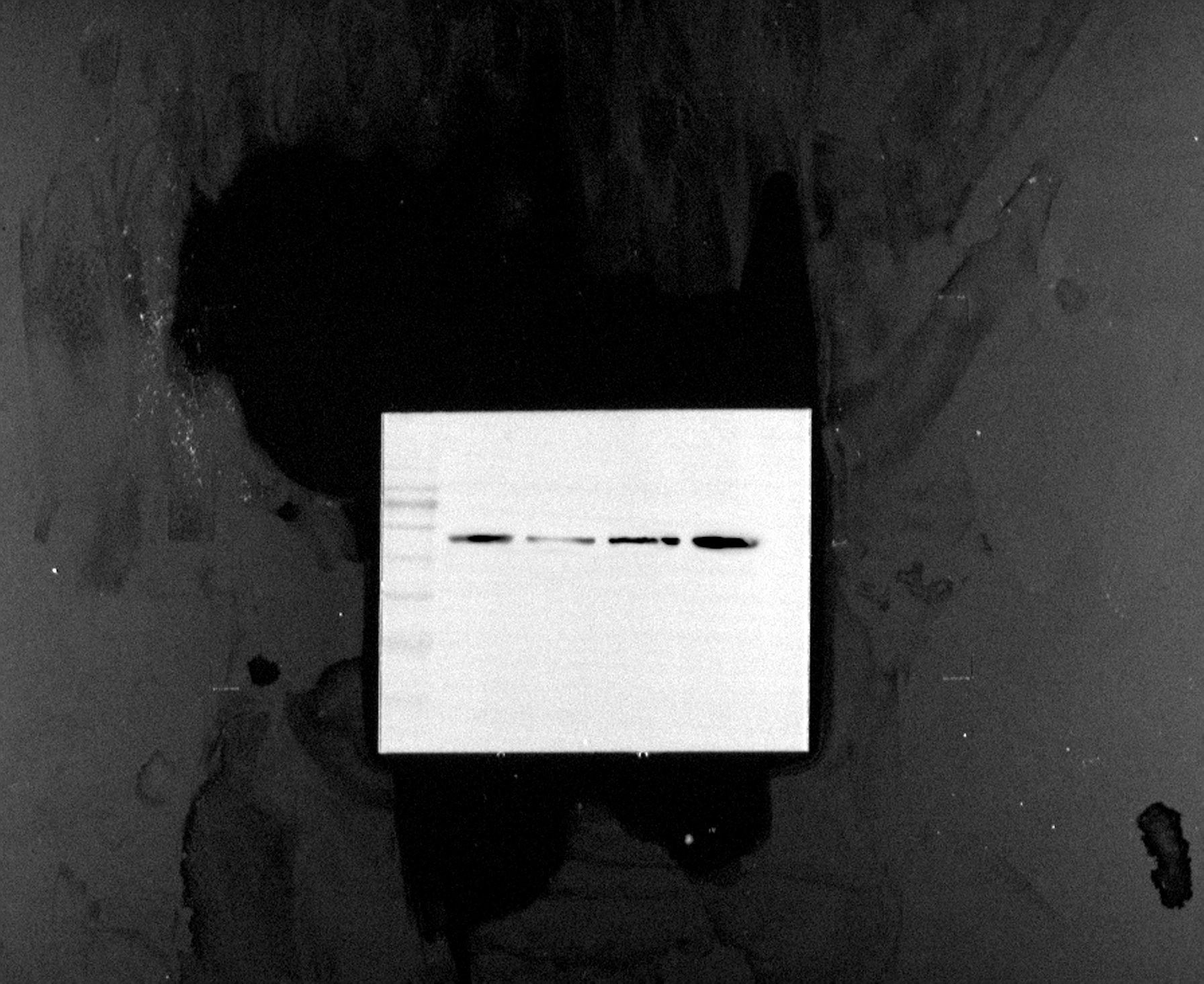

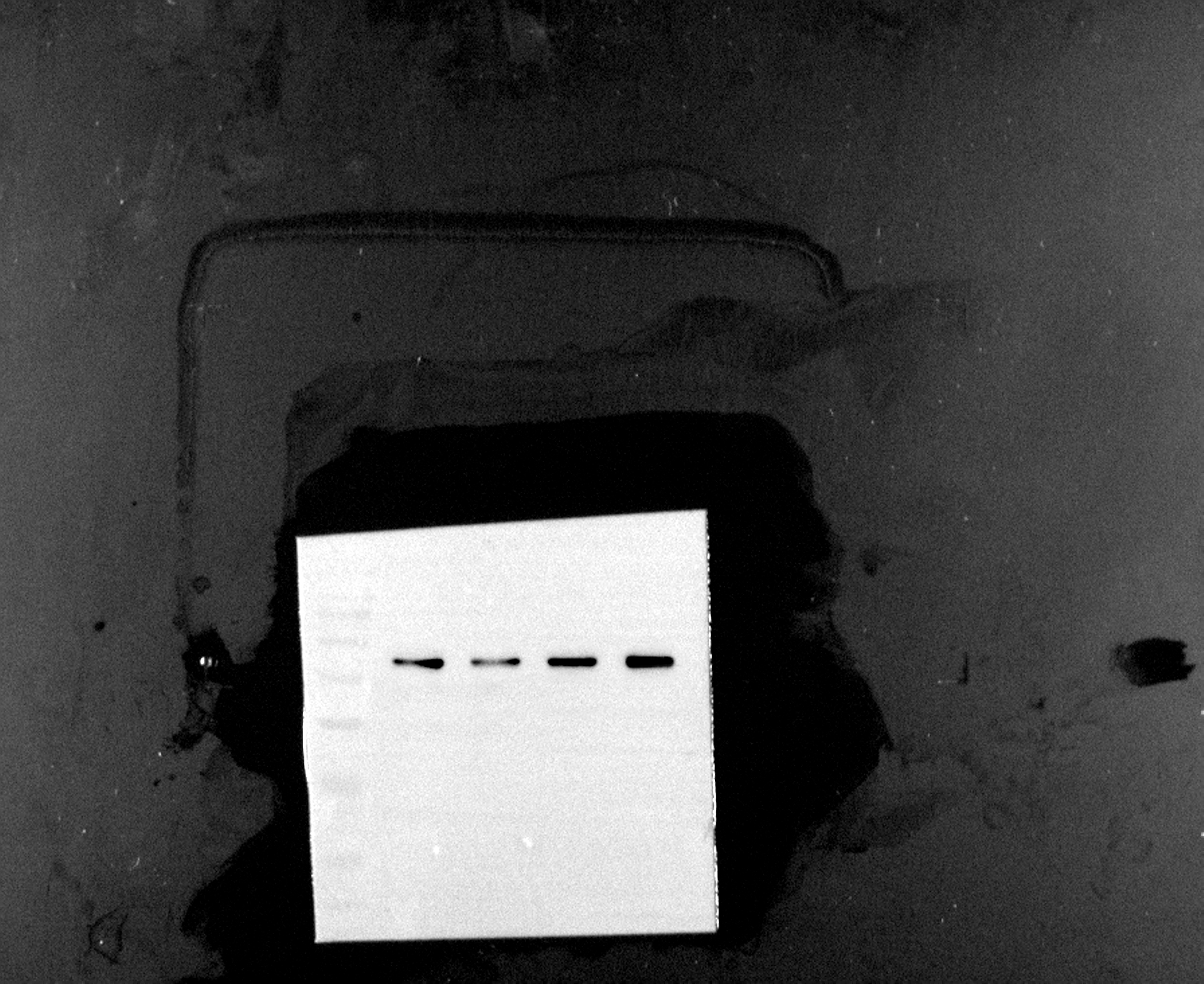


SCC-25 p-PI3K SCC-9 p-PI3K


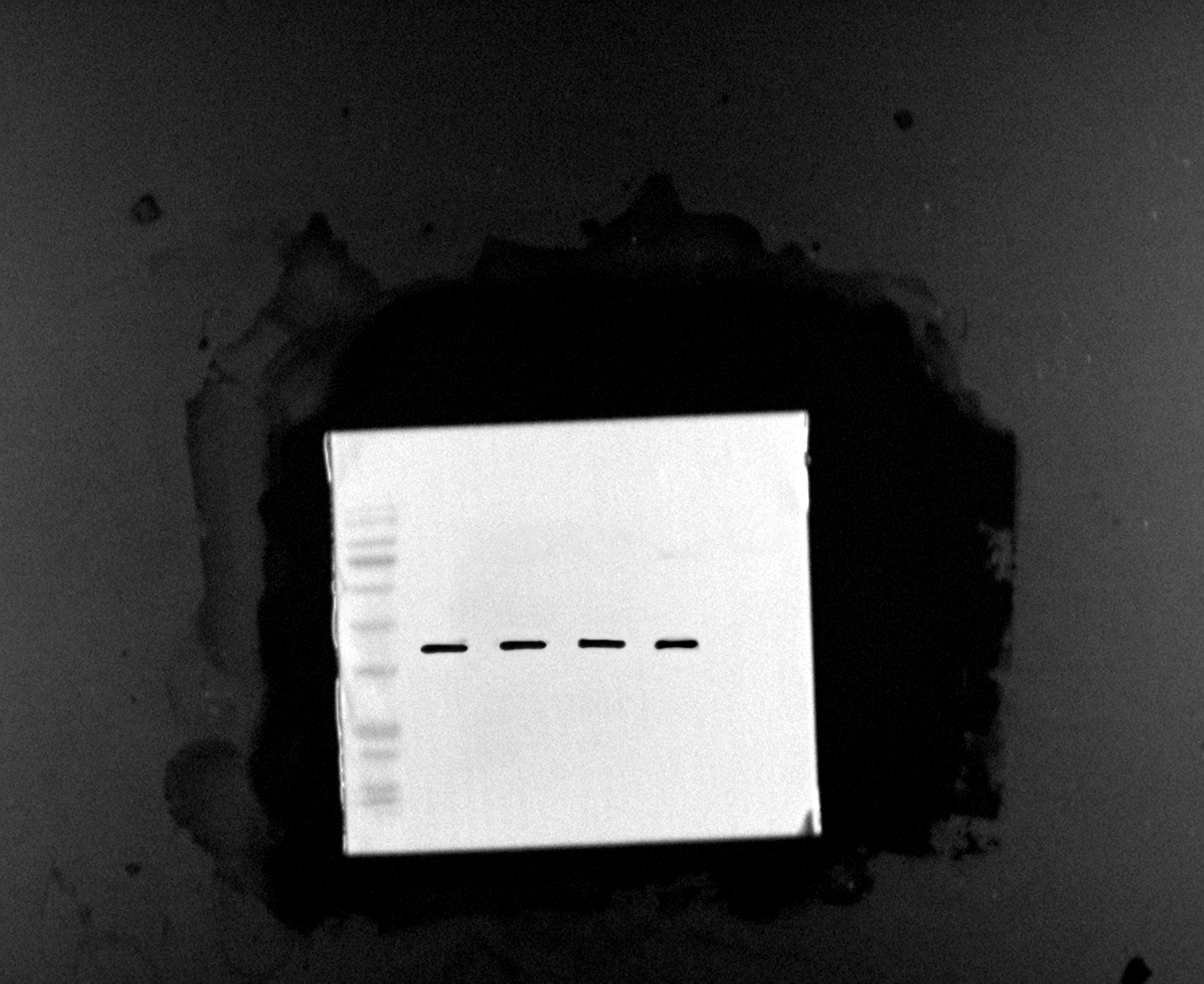

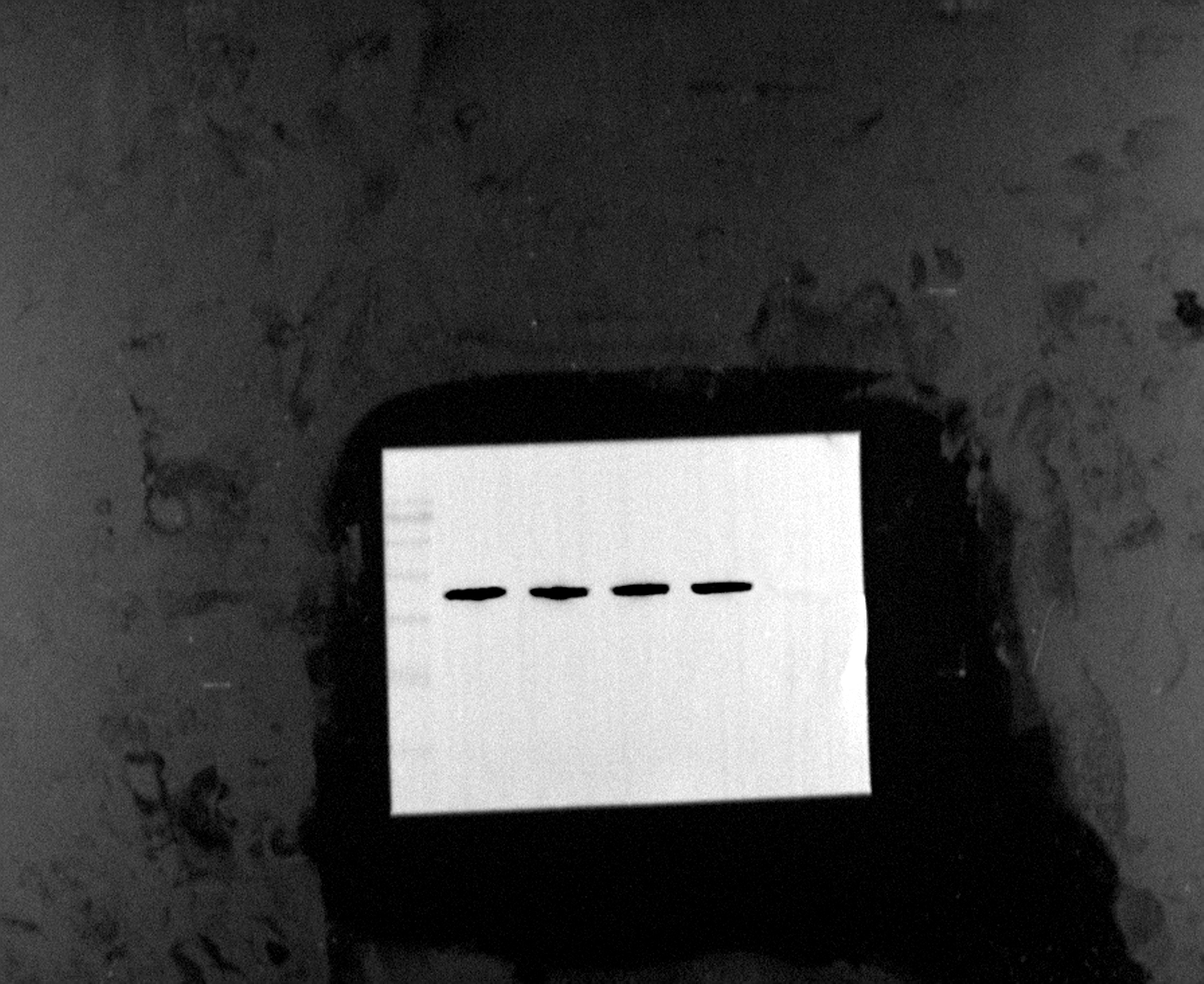


SCC-25 AKT SCC-9 AKT


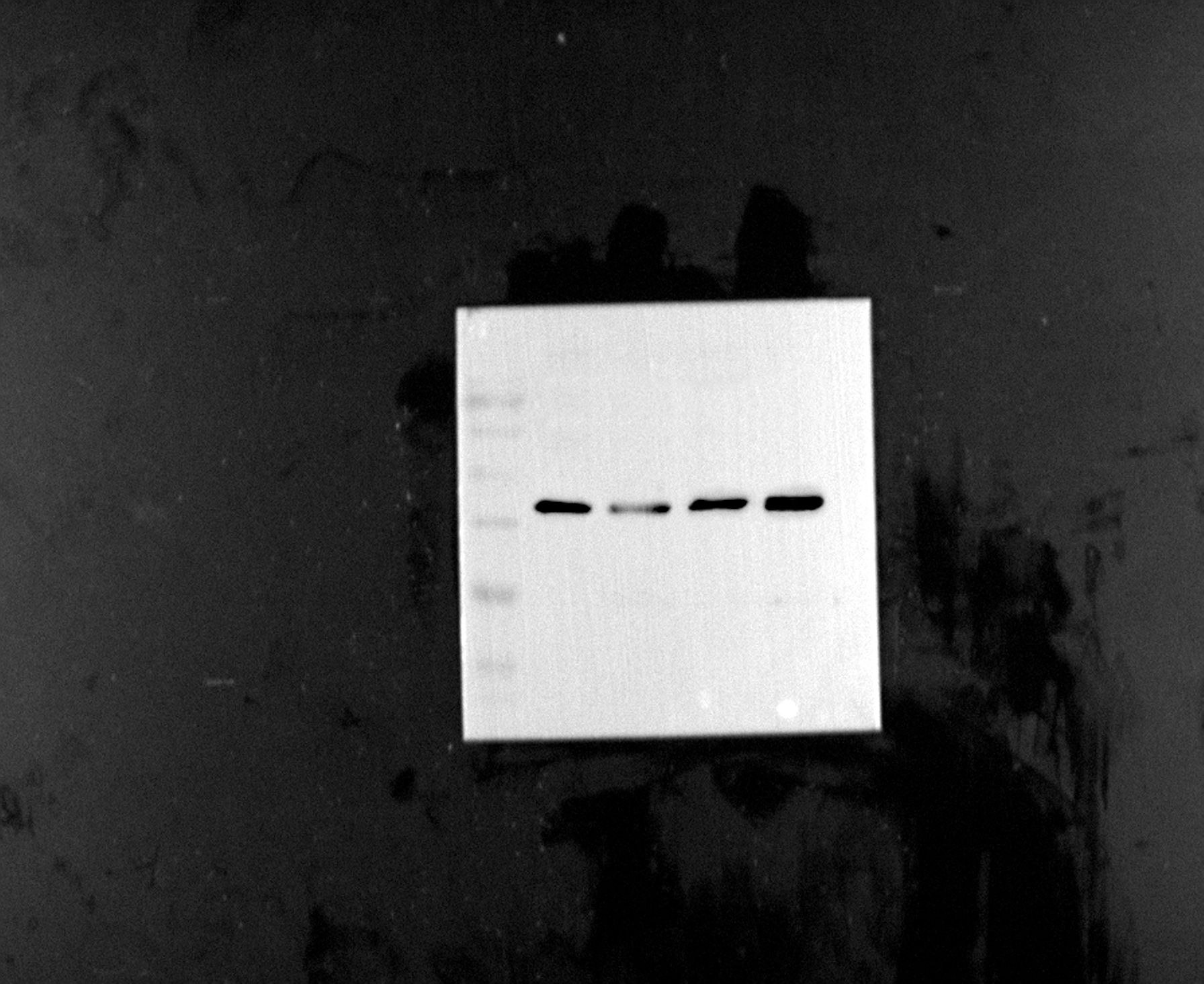

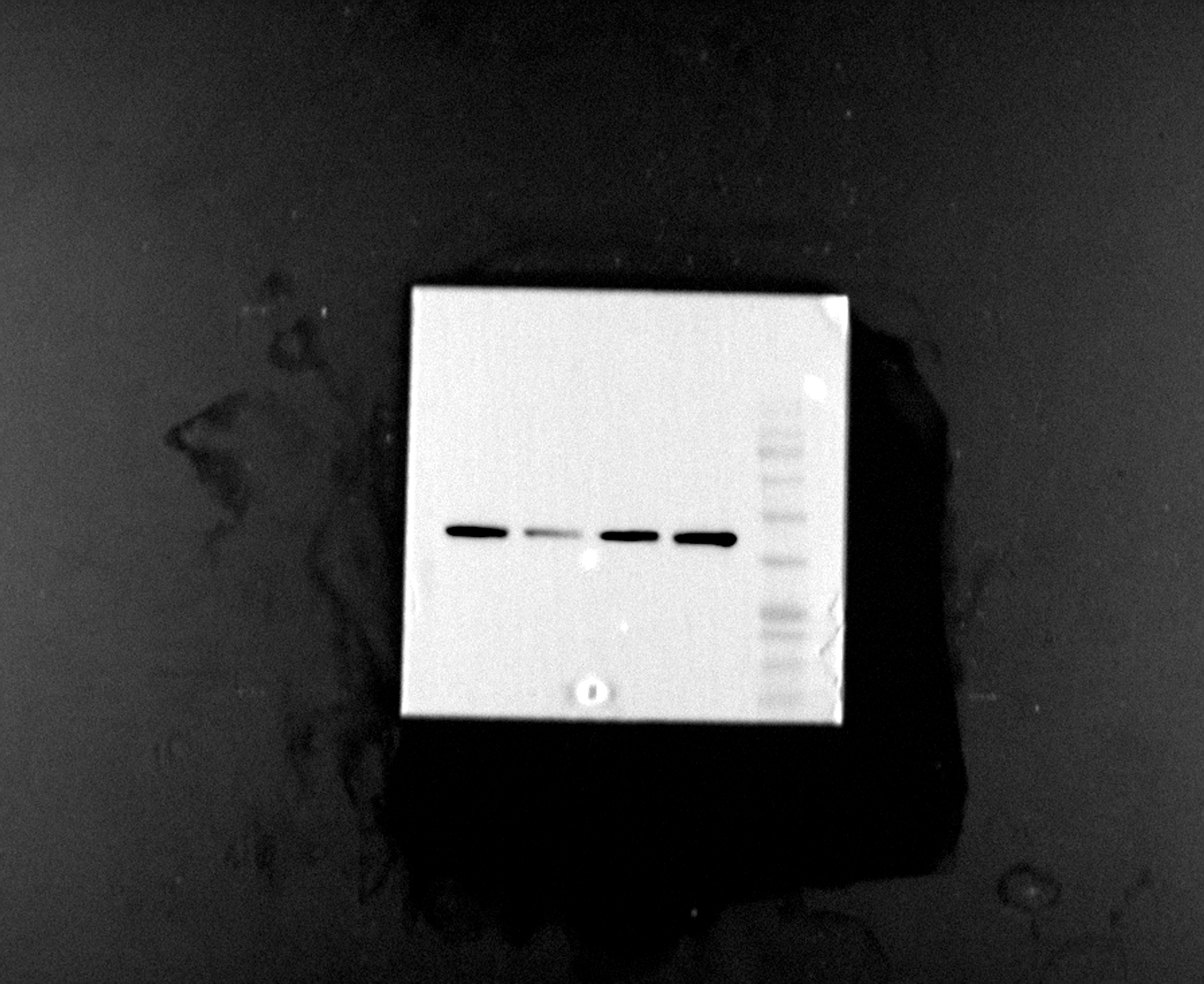


SCC-25 p-AKT SCC-9 p-AKT

**Fig.5B:**


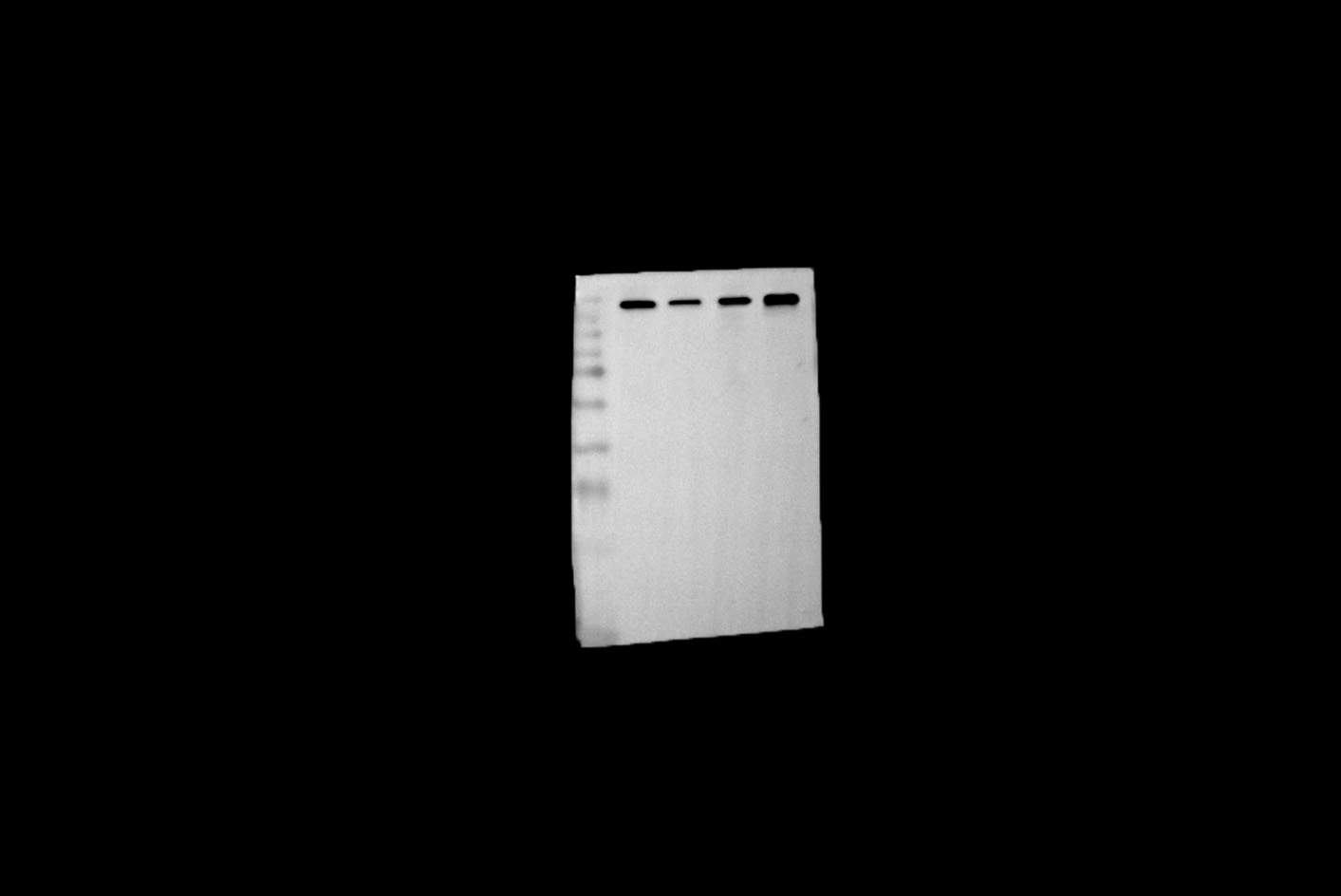

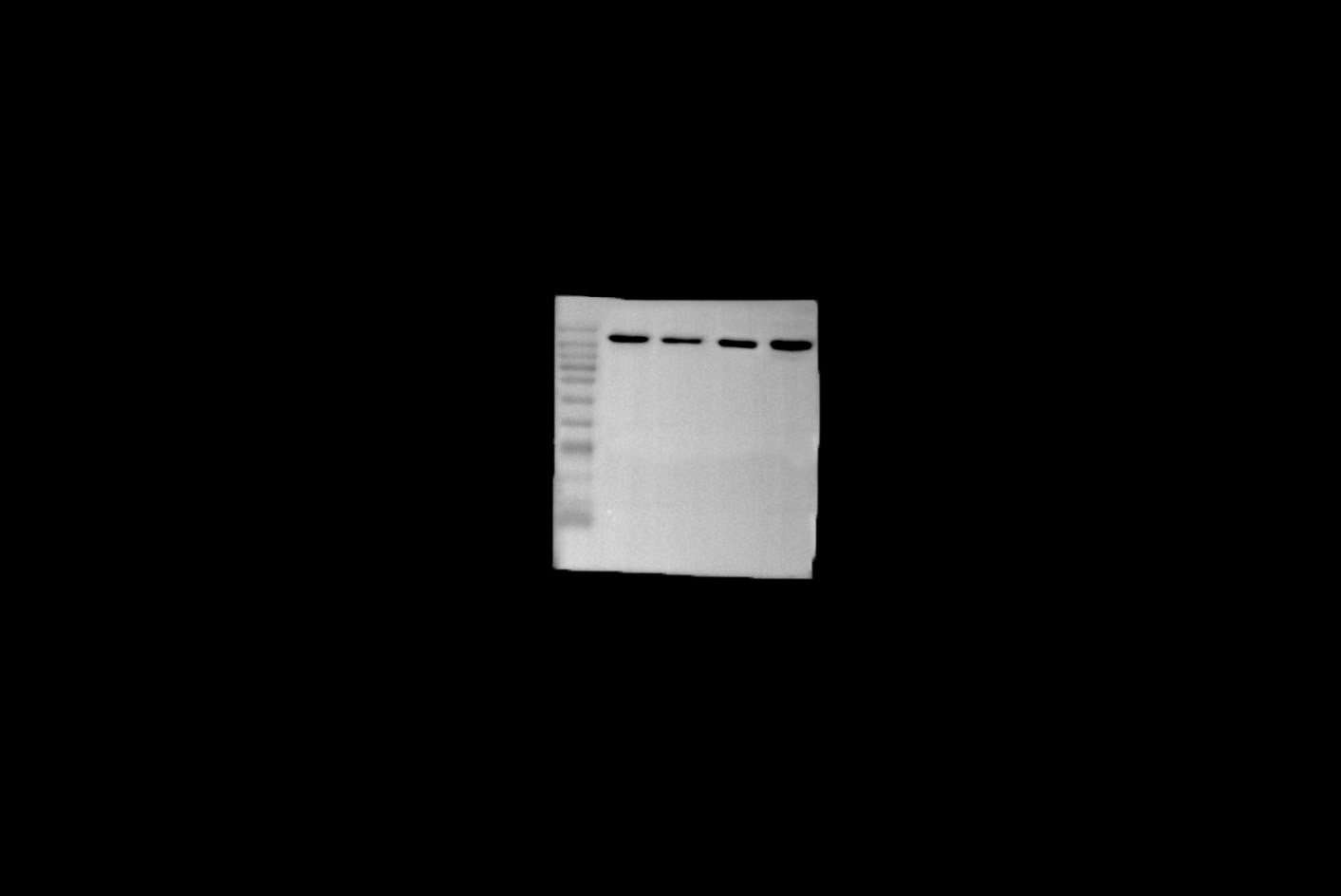


SCC-25 collagen Ι SCC-9 collagen Ι


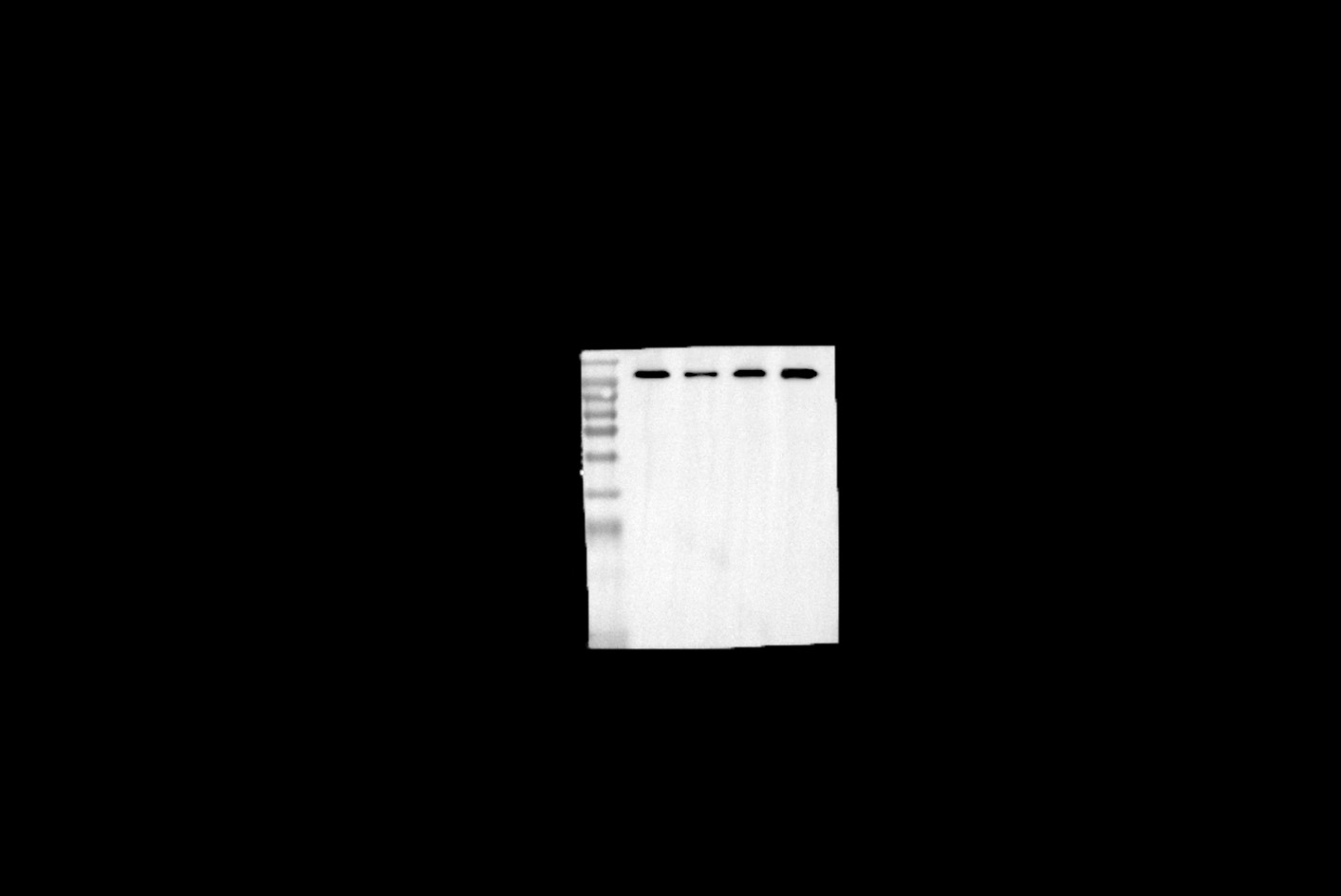

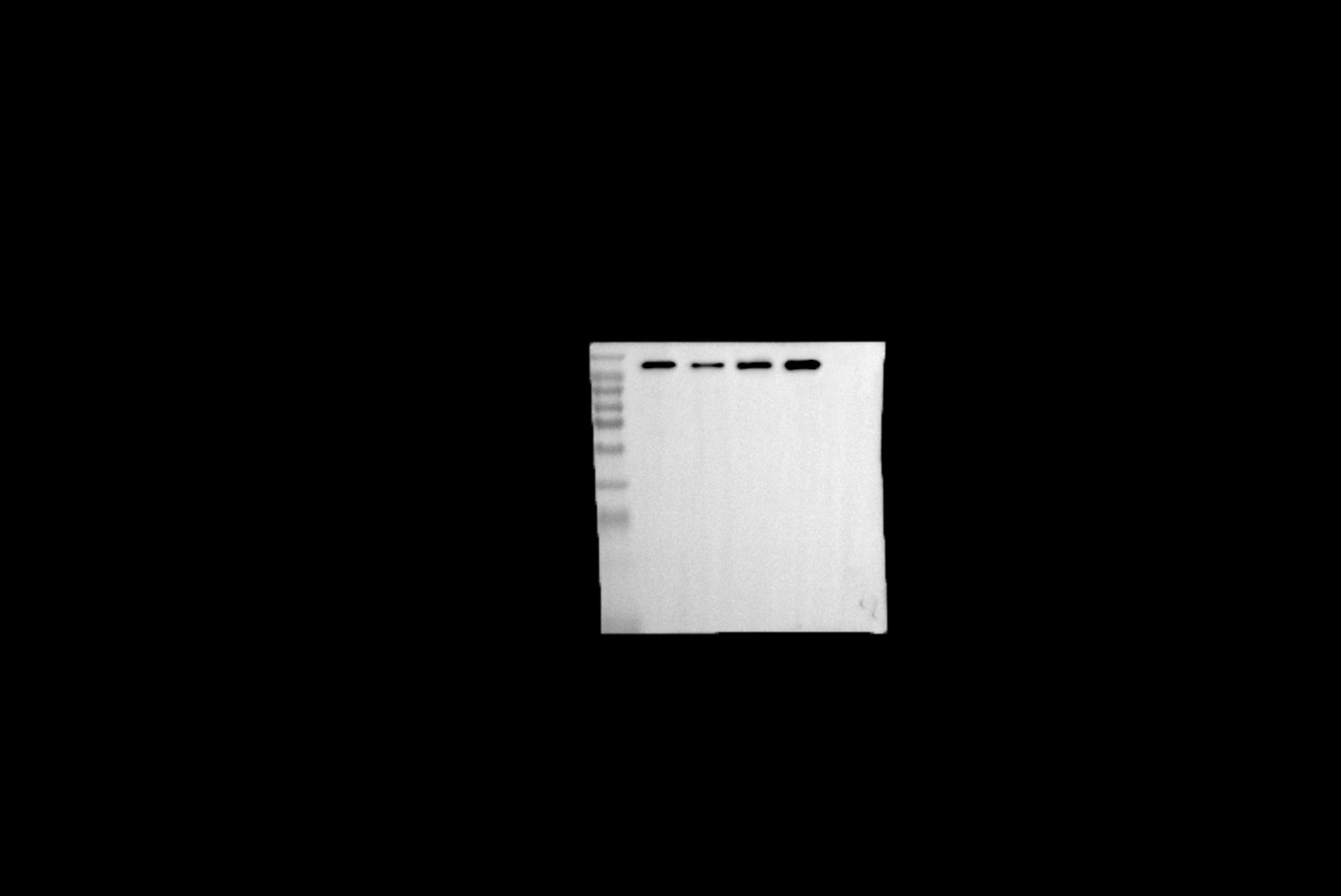


SCC-25 collagen Ⅳ SCC-9 collagen Ⅳ


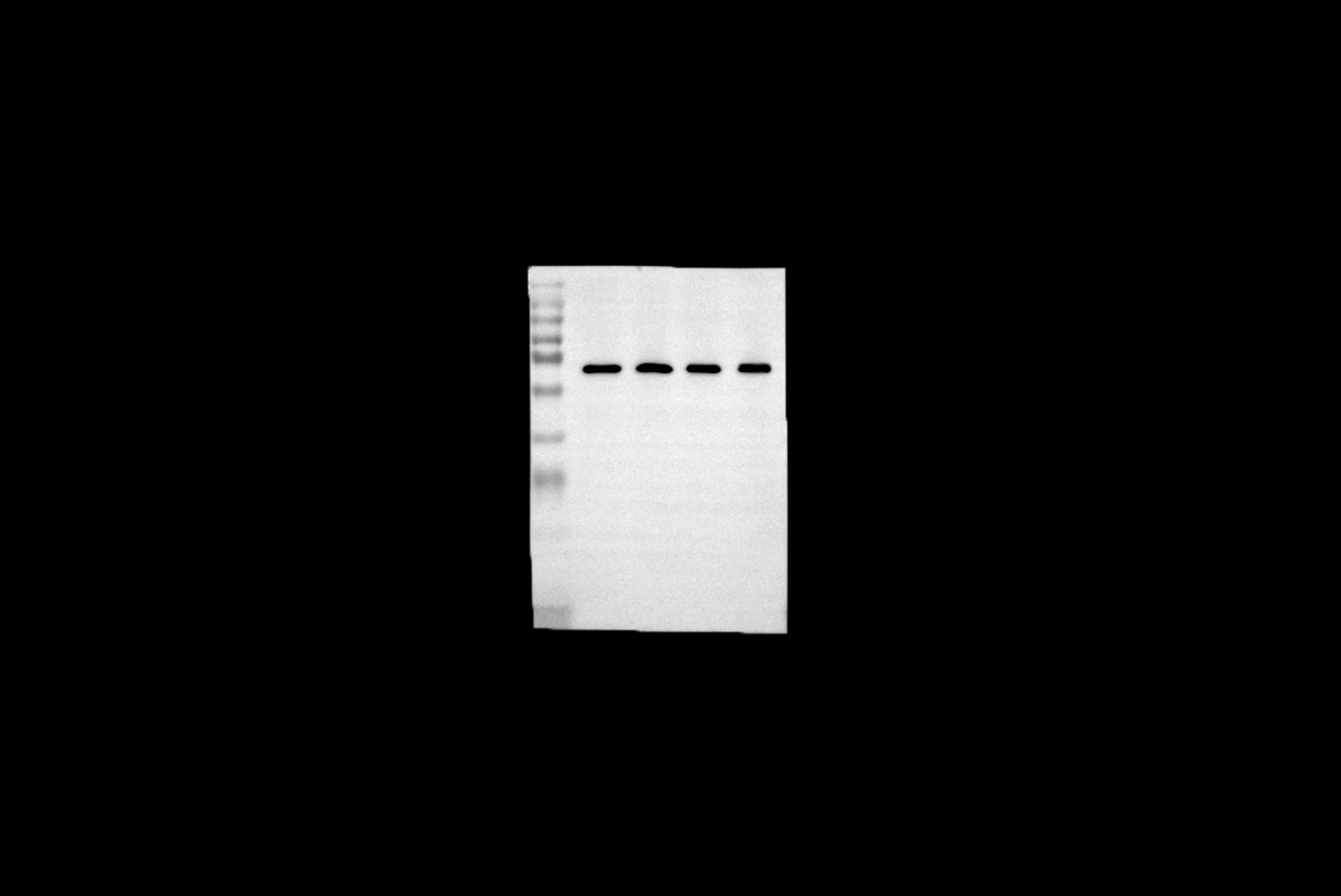

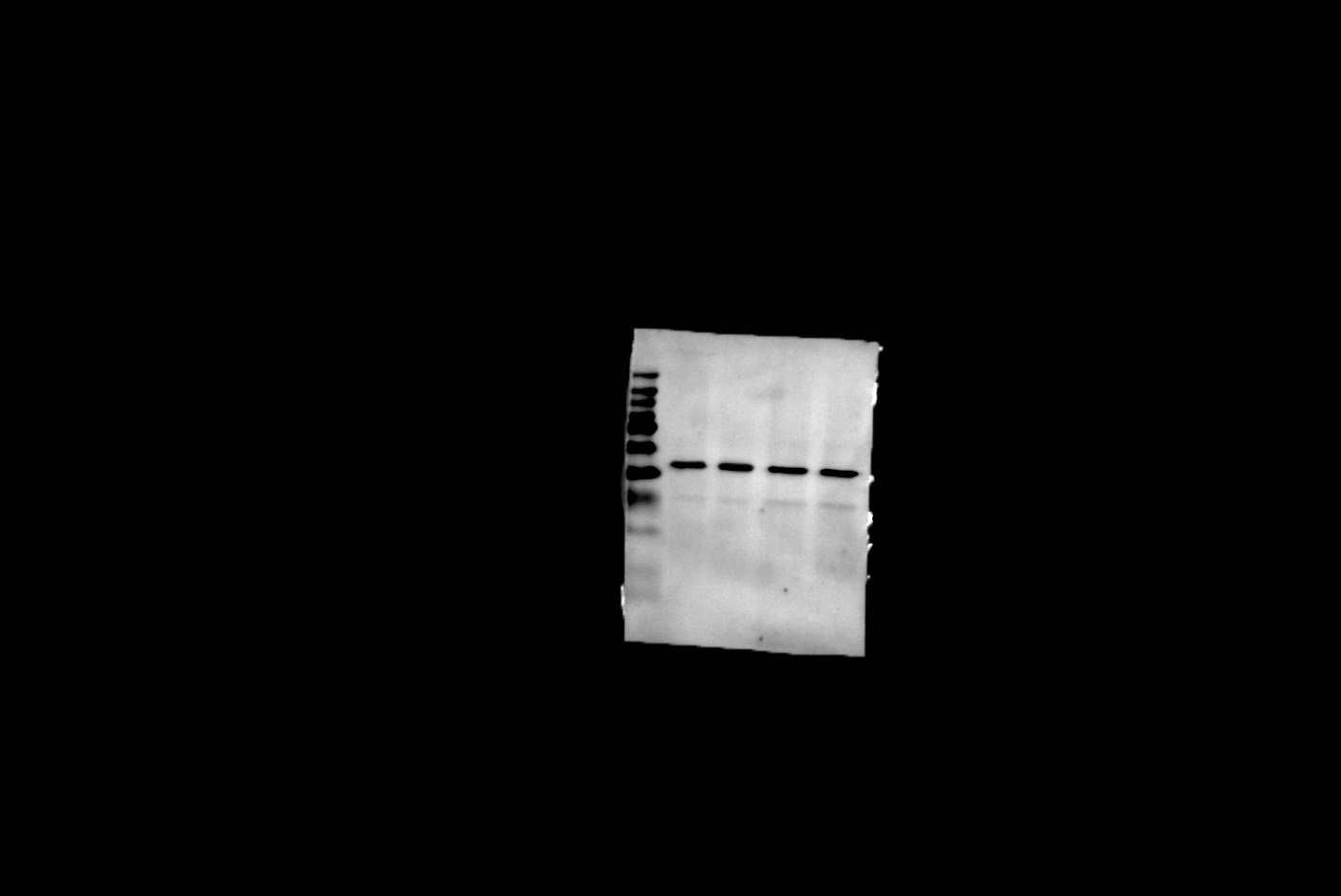


SCC-25 GAPDH SCC-9 GAPDH

**Fig.5C:**


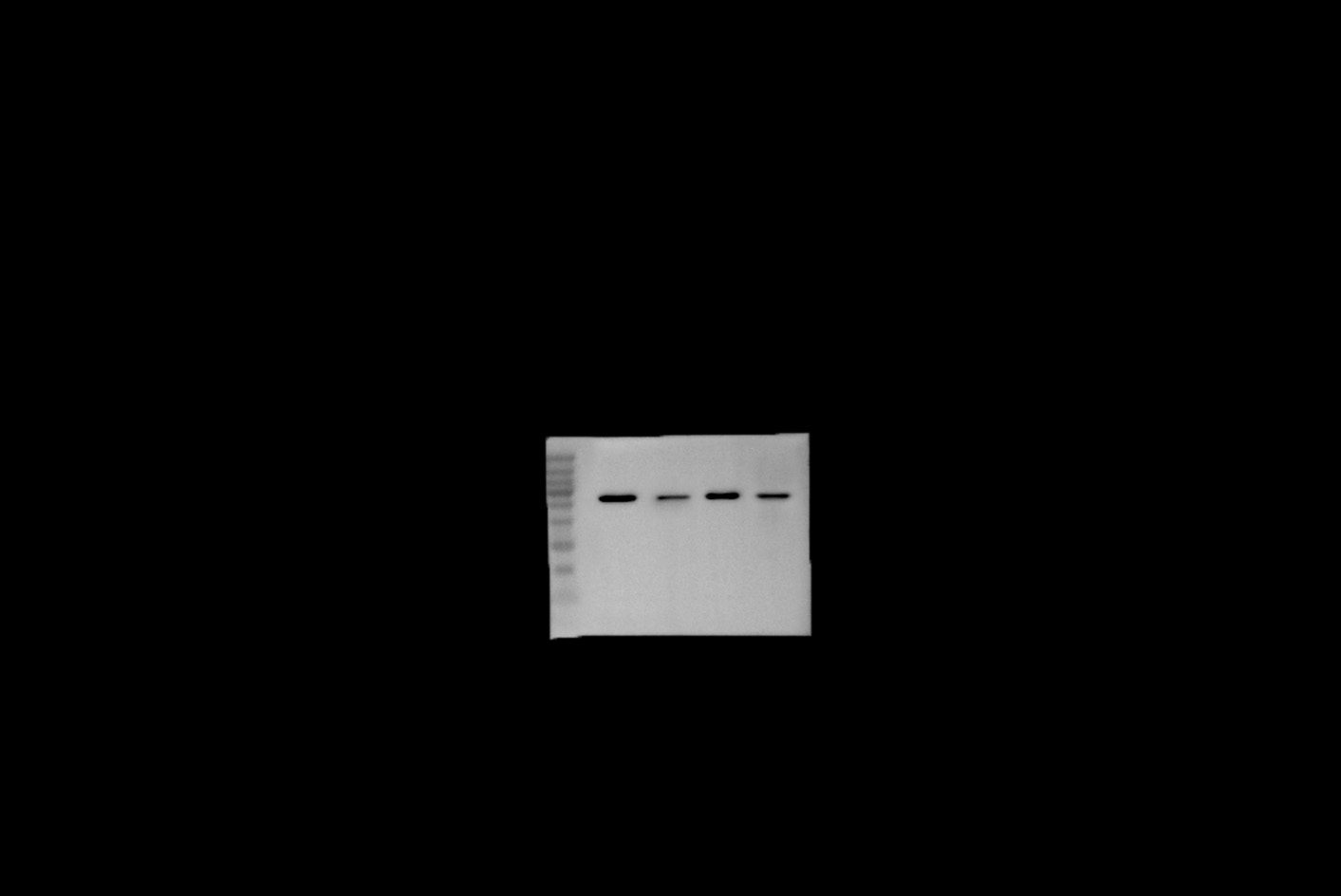

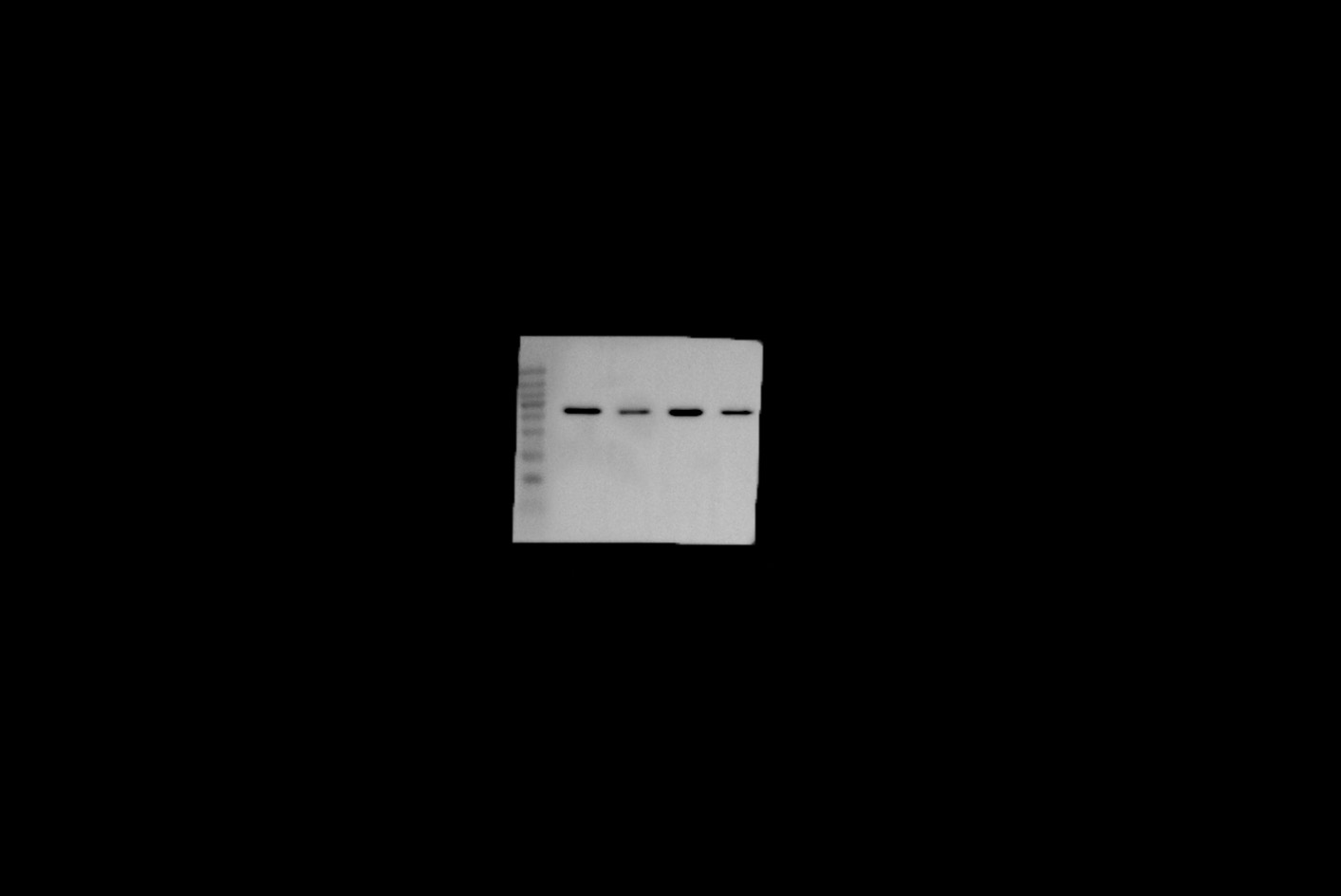


SCC-25 P4HA2 SCC-9 P4HA2


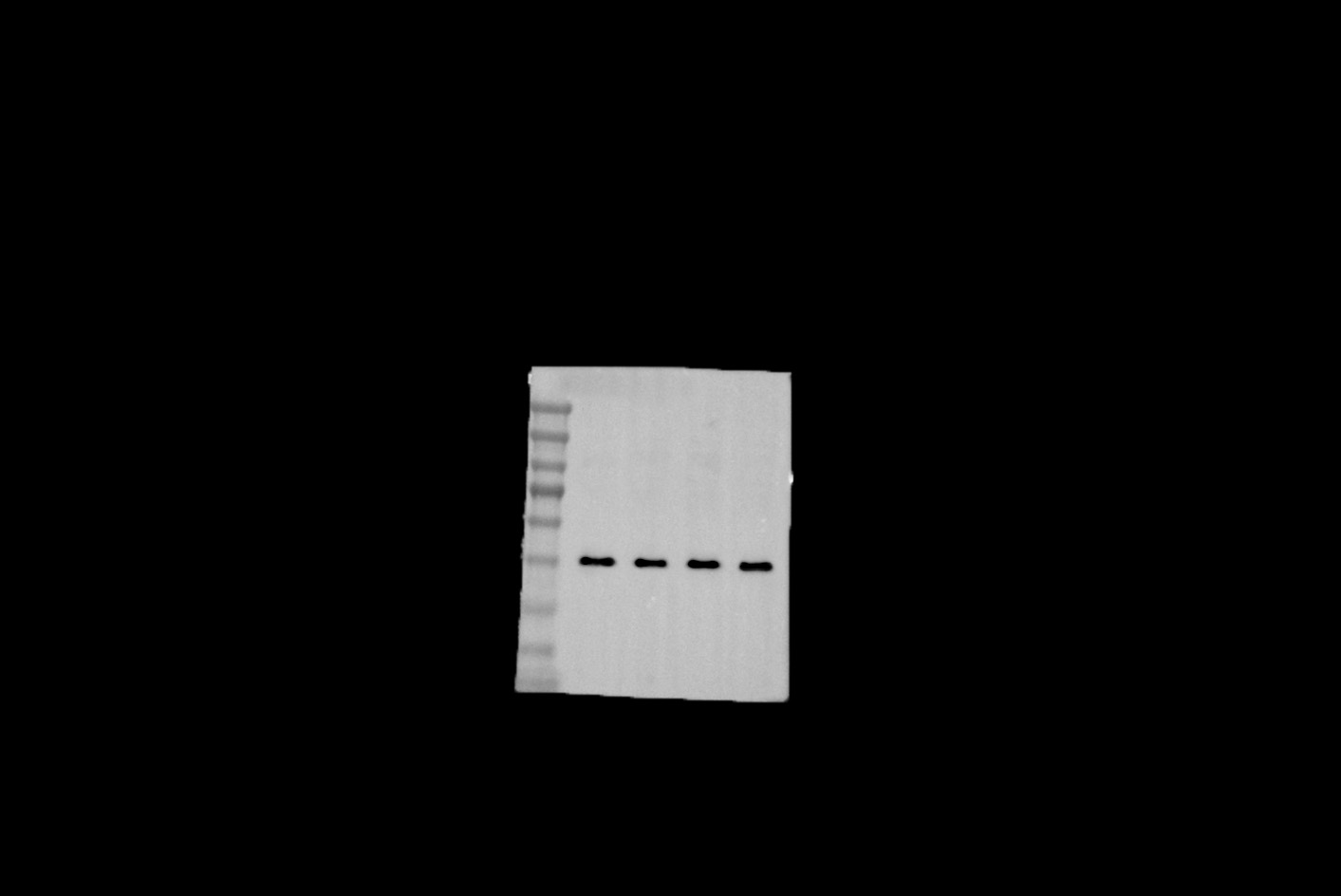

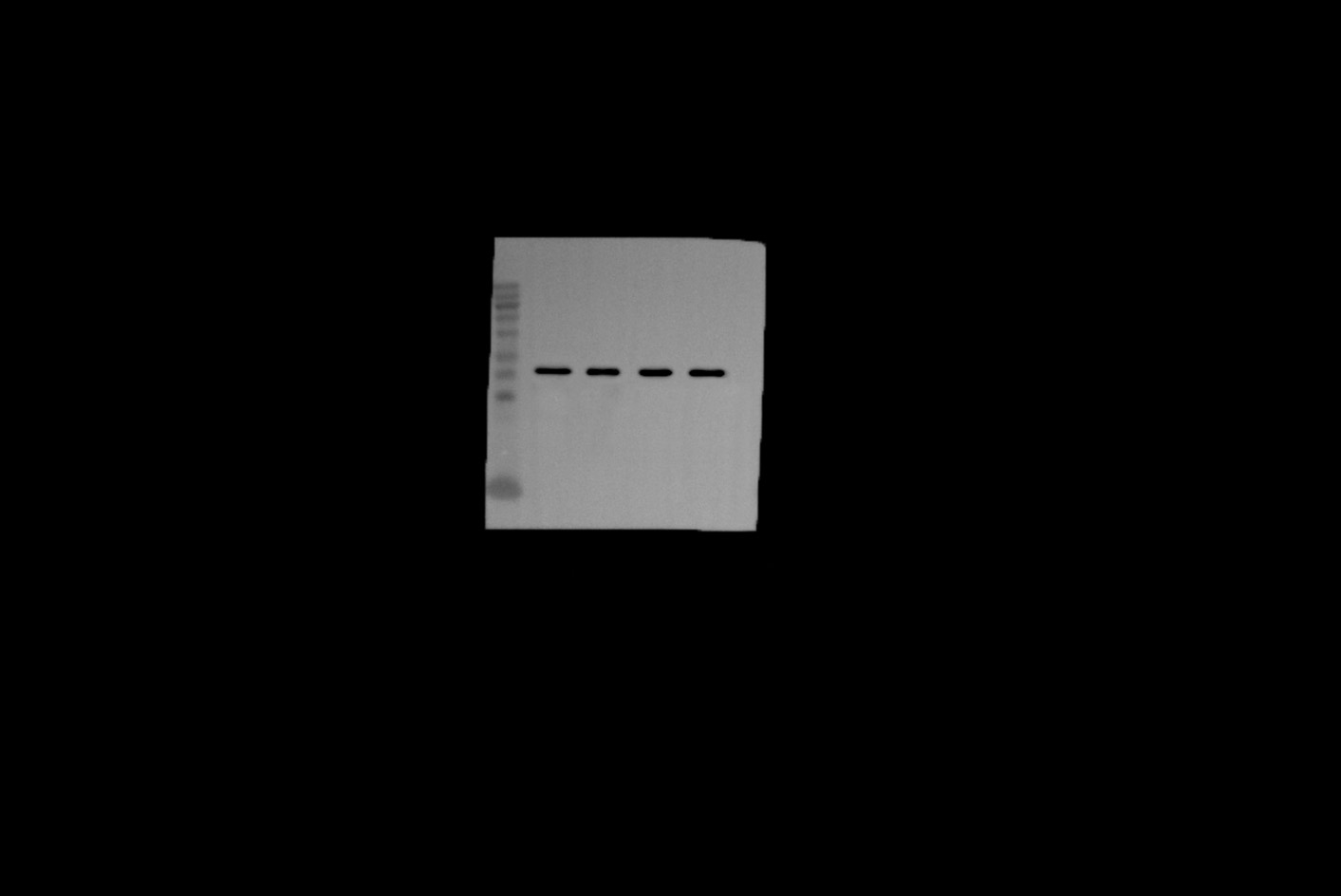


SCC-25 PI3K SCC-9 PI3K


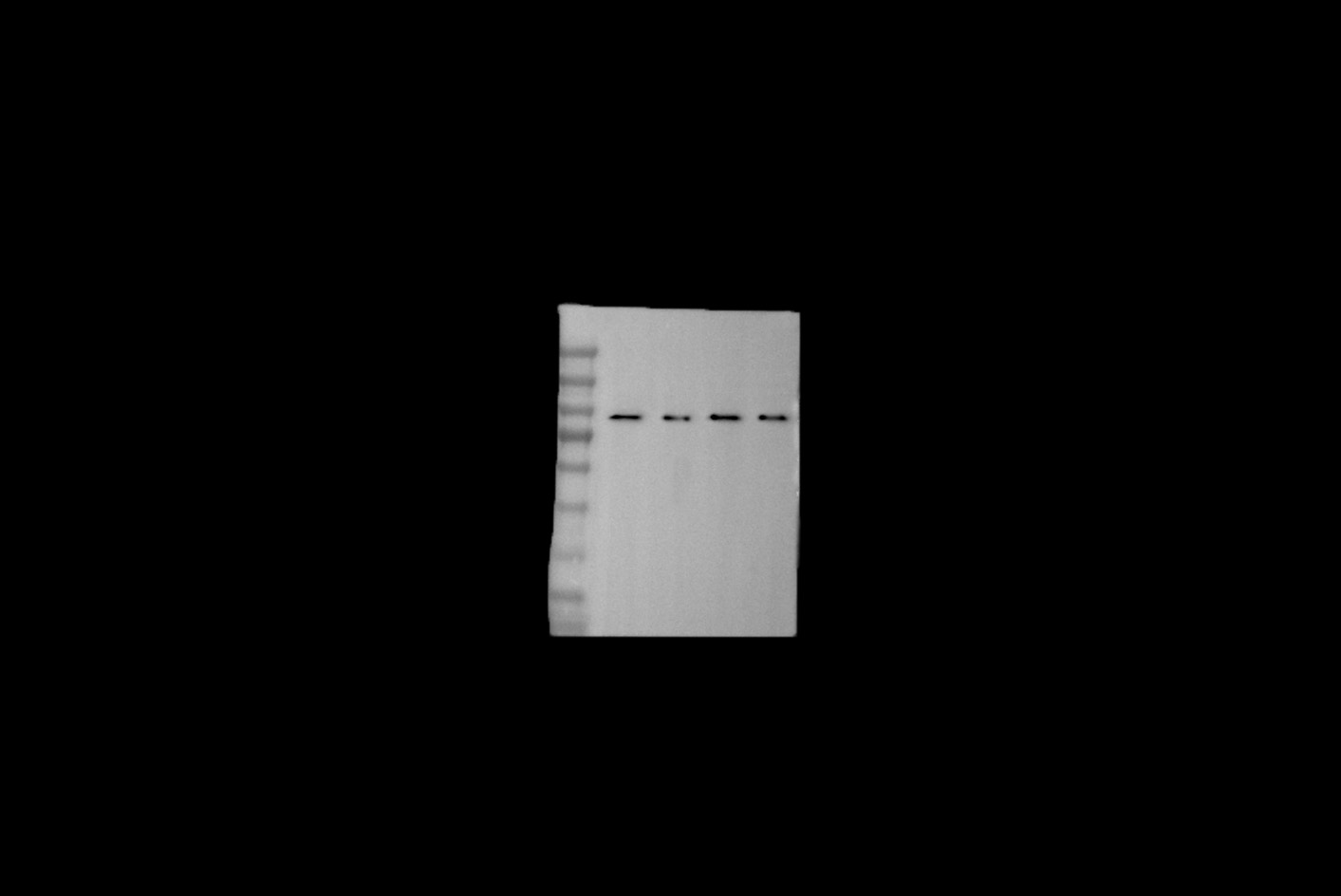

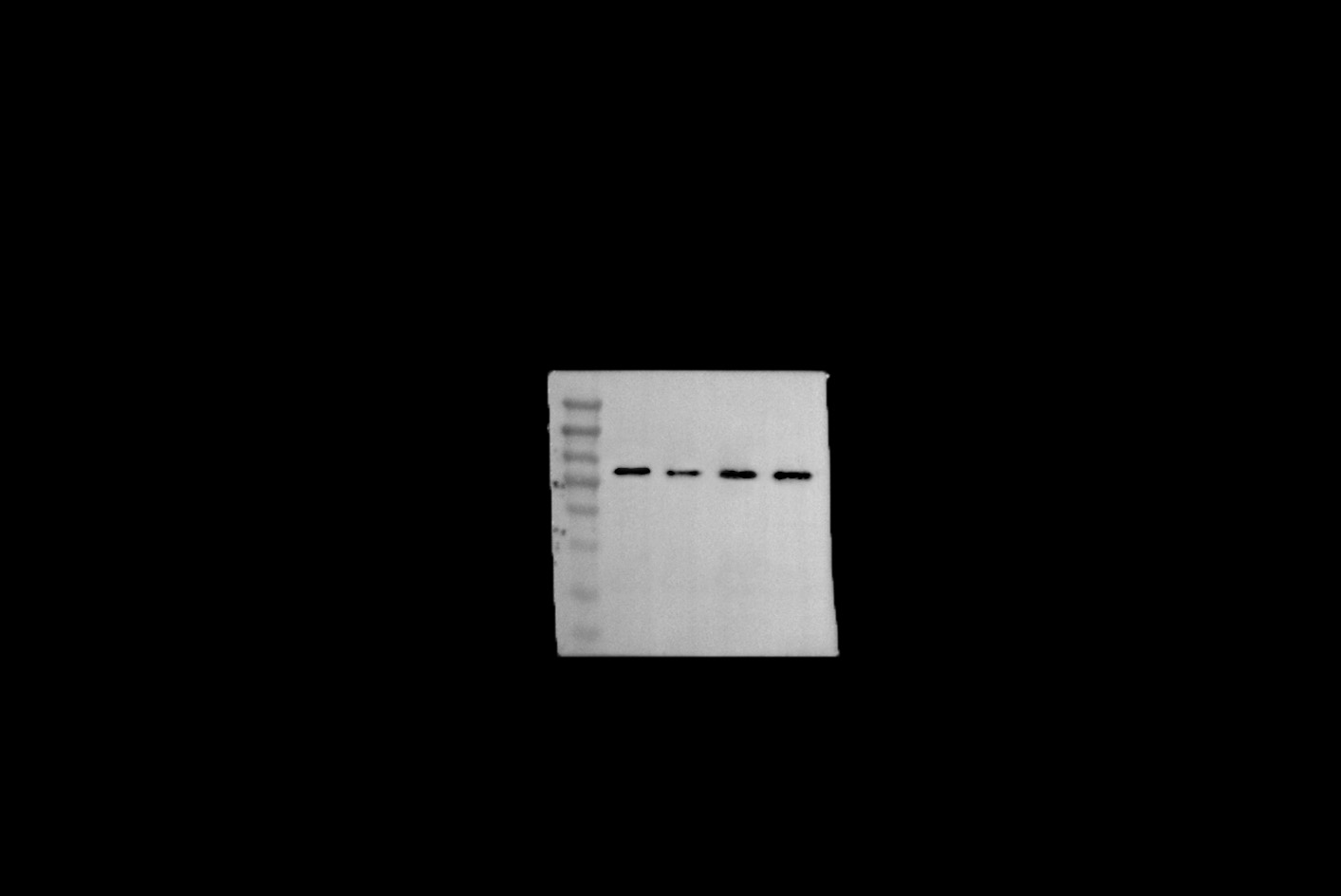


SCC-25 p-PI3K SCC-9 p-PI3K


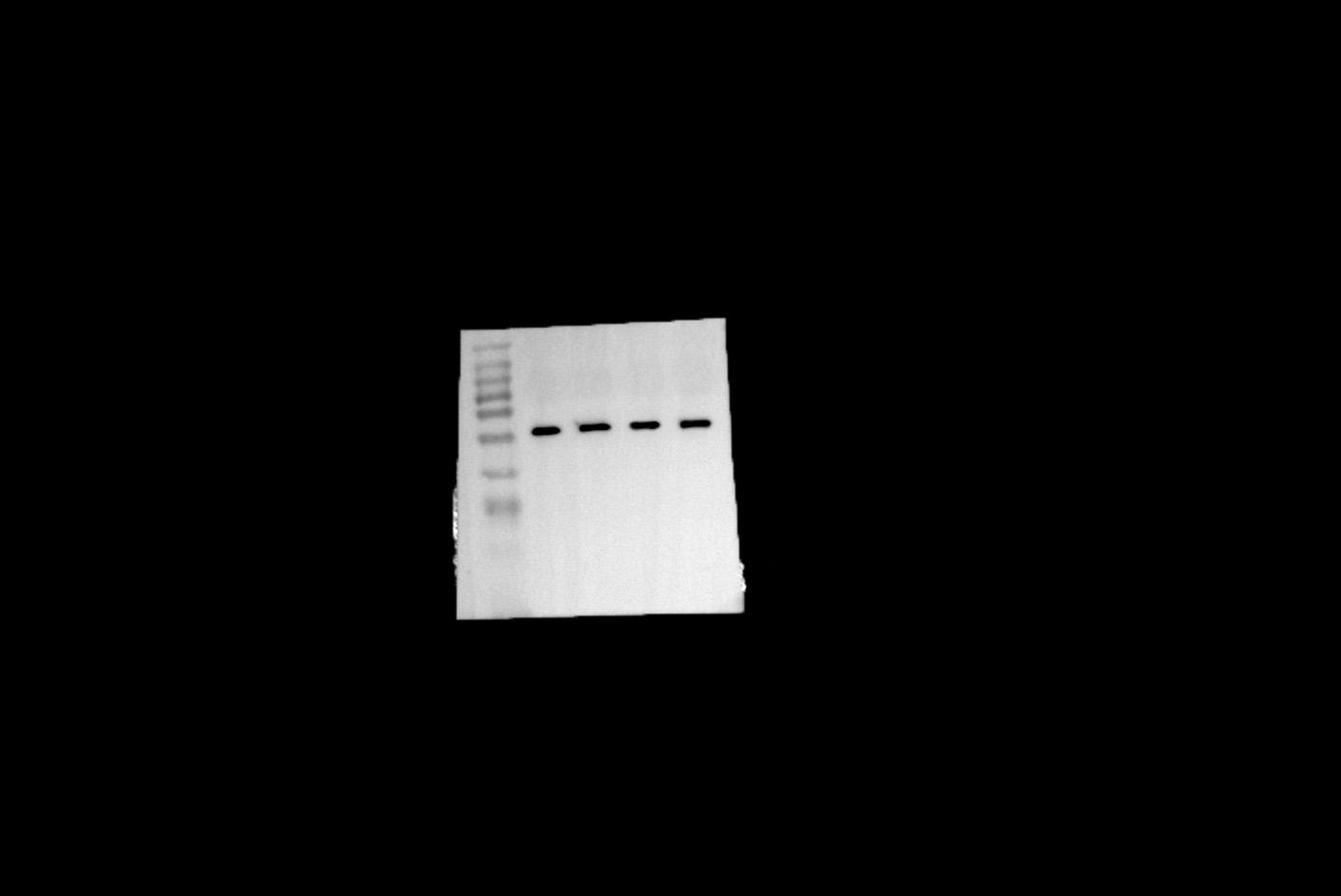

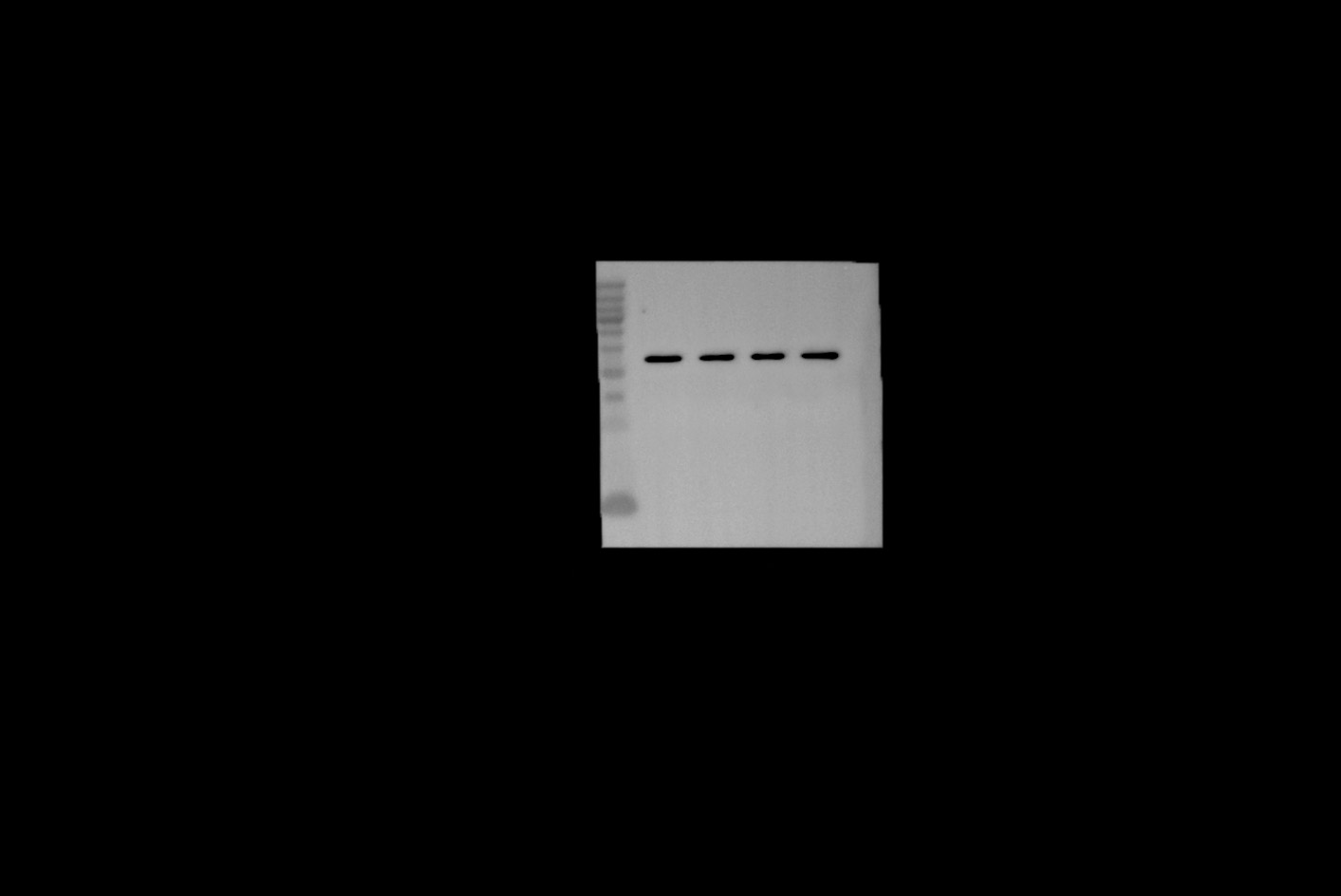


SCC-25 AKT SCC-9 AKT


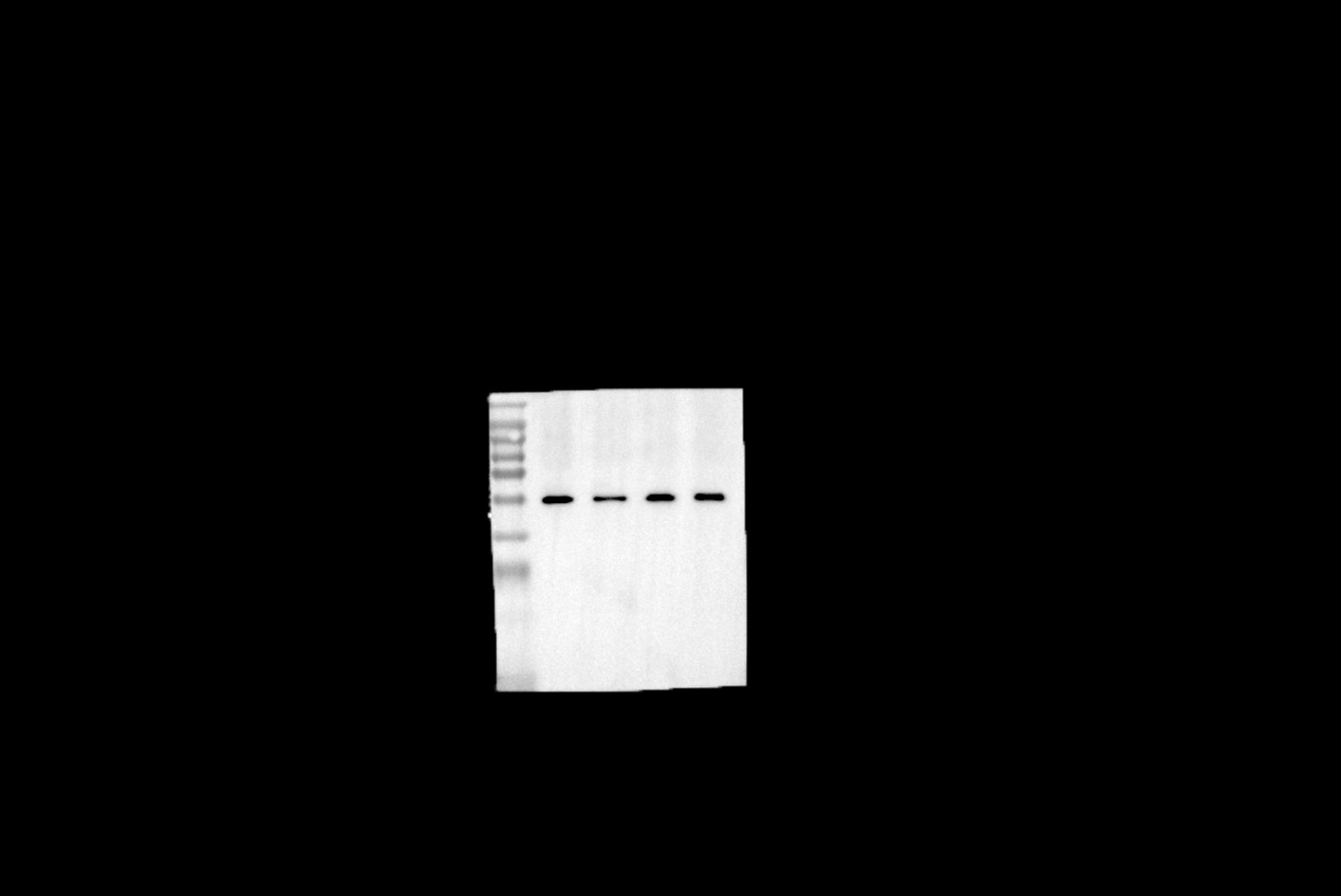

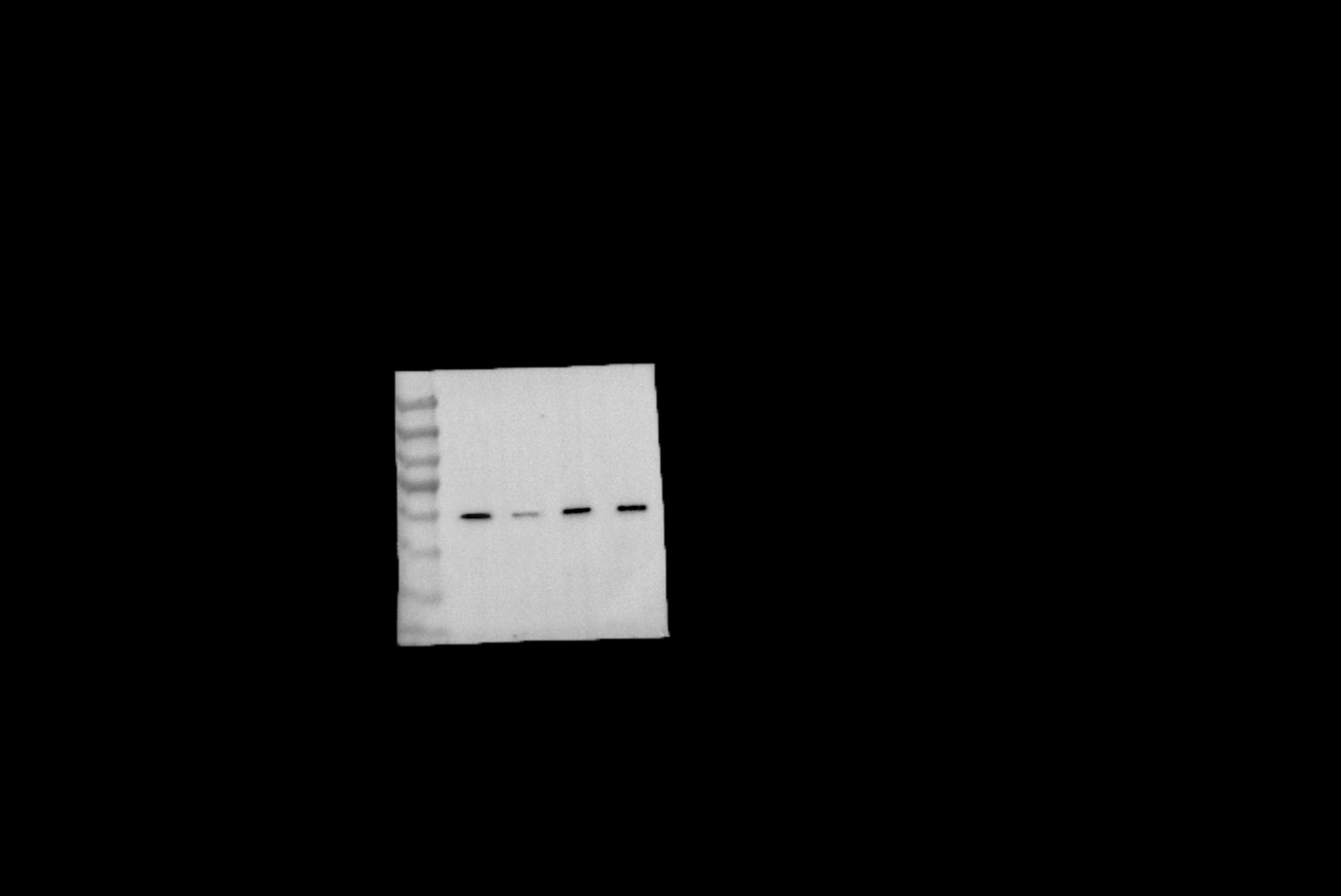


SCC-25 p-AKT SCC-9 p-AKT


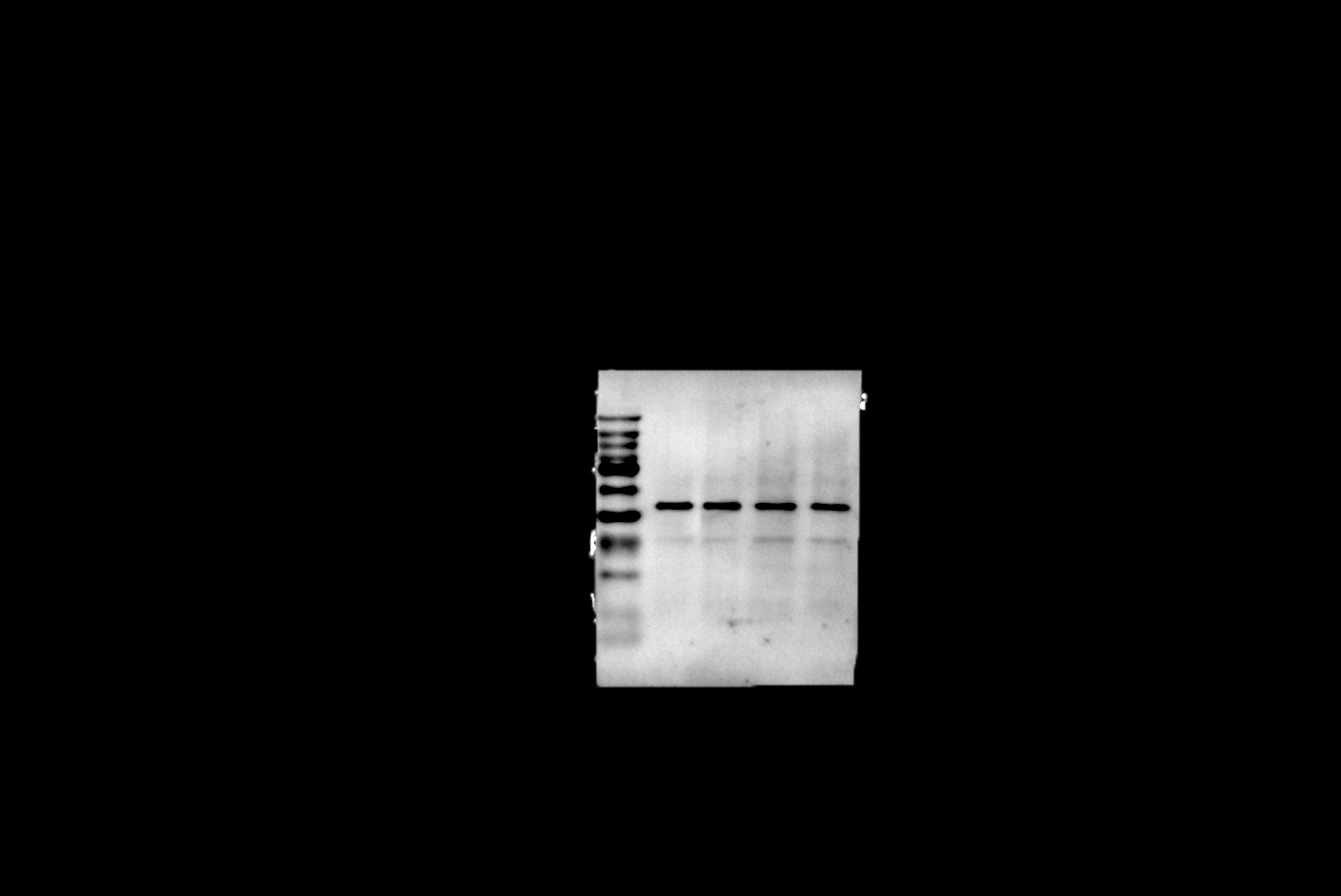

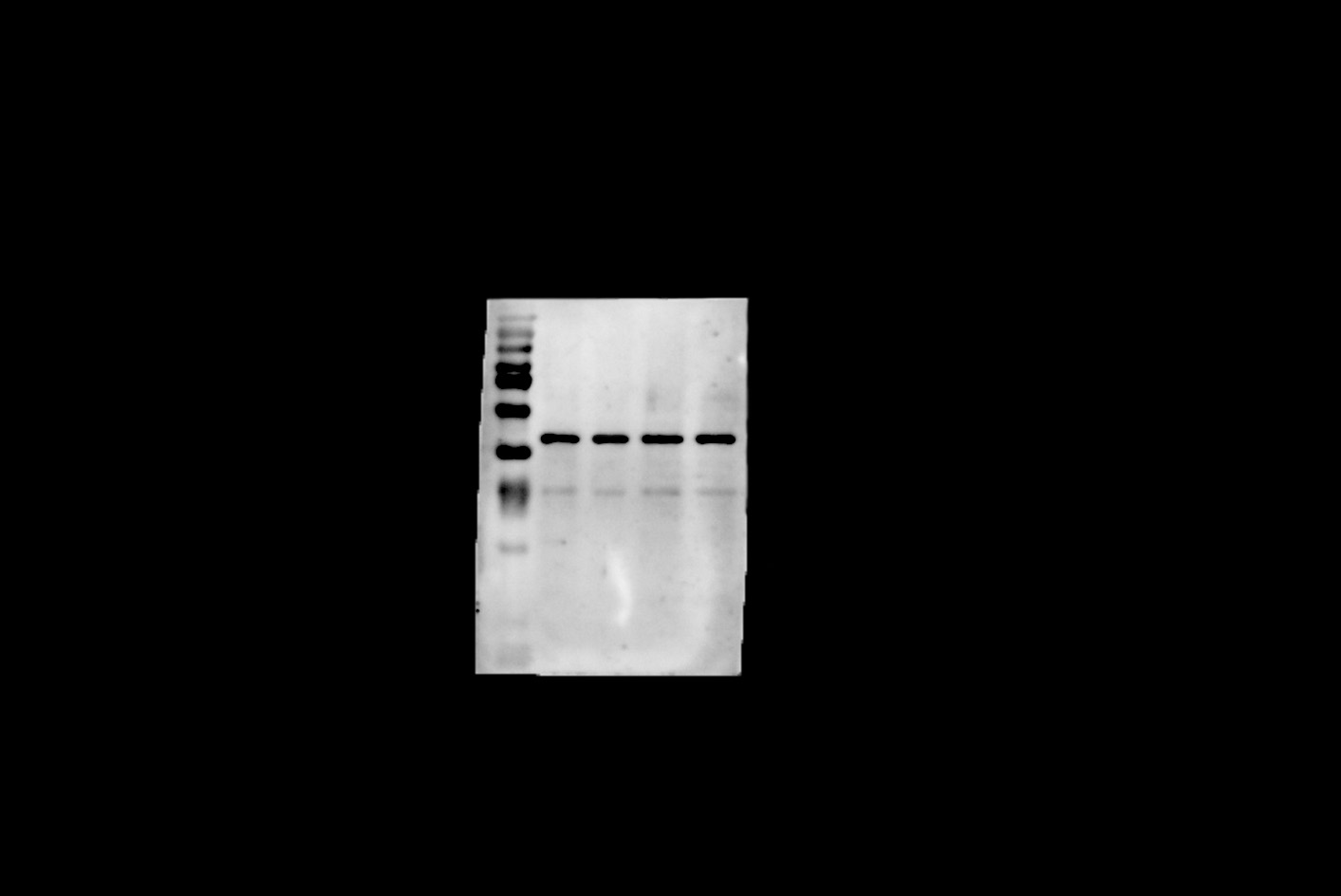


SCC-25 GAPDH SCC-9 GAPDH
