# Supplementary material for: P4HA2 promotes proliferation, invasion, and metastasis through regulation of the PI3K/AKT signaling pathway in oral squamous cell carcinoma
Source: Sci Rep. 2024 Jul 1;14:15023. doi: 10.1038/s41598-024-64264-5 (PMC11217378; doi:10.1038/s41598-024-64264-5)
Supplement: Supplementary file 2 — Supplementary Information 2. [file 41598_2024_64264_MOESM2_ESM.docx]

**Table 1 Interference sequence**

| Gene name | Top strand | Bottom strand |
| --- | --- | --- |
| sh-NC | GATCCGTTCTCCGAACGTGTCACGTAAT  TCAAGAGATTACGTGACACGTTCGGAGA  ATTTTTTC | AATTGAAAAAATTCTCCGAACGTGTCAC  GTAATCTCTTGAATTACGTGACACGTTC  GGAGAACG |
| sh-P4HA2 | GATCCGCAGCTGTGTTCTGGTACAACCT  CTTCTCGAGAAGAGGTTGTACCAGAACA  CAGCTGTTTTTTG | AATTCAAAAAACAGCTGTGTTCTGGTAC  AACCTCTTCTCGAGAAGAGGTTGTACCA  GAACACAGCTGCG |

**Table 2 The primer sequences**

| The name of the primer | sequences |
| --- | --- |
| P4HA2 forward primer | 5’-CAAACTGGTGAAGCGGCTAAA-3’ |
| P4HA2 reverse primer | 5'-GCACAGAGAGGTTGGCGATA-3' |
| GAPDH forward primer | 5′-CACCCACTCCTCCACCTTTG-3′ |
| GAPDH reverse primer | 5′-CCACCACCCTGTTGCTGTA G-3′ |

**Table 3** Relationship of P4HA2 expression and clinicopathological features exhibited by the OSCC tissues

| Clinicopathological characteristics | Total(n) | P4HA2-positive | P4HA2-negative | *χ*^2^ | *P*-vague |
| --- | --- | --- | --- | --- | --- |
| Gender |  |  |  |  |  |
| Male | 23 | 14 | 9 | 0.5447 | 0.4605 |
| Female | 21 | 15 | 6 |  |  |
| Age(years) |  |  |  |  |  |
| ＞60 | 28 | 19 | 9 | 0.13 | 0.7184 |
| ≤60 | 16 | 10 | 6 |  |  |
| Tumor stage |  |  |  |  |  |
| Ι＋Ⅱ | 23 | 10 | 13 | 10.79 | 0.001* |
| Ⅲ＋Ⅳ | 21 | 19 | 2 |  |  |
| Differentiation |  |  |  |  |  |
| Well | 27 | 14 | 13 | 6.879 0.0321* | |
| Moderate | 10 | 8 | 2 |  |  |
| Poor | 7 | 7 | 0 |  |  |
| Lymph node metastasis |  |  |  |  |  |
| Positive | 17 | 16 | 1 | 9.811 | 0.0017* |
| Negative | 27 | 13 | 14 |  |  |
| Depth of invasion |  |  |  |  |  |
| ＜0.5 | 15 | 8 | 7 | 4.300 | 0.1165 |
| 0.5-1.0 | 18 | 11 | 7 |  |  |
| ＞1.0 | 11 | 10 | 1 |  |  |

**Table 4** COX regression analysis of clinical features in connection with OS in the HNSC

| Variable | Univariate analysis | | Multivariate analysis | |
| --- | --- | --- | --- | --- |
|  | HR (95%CI) | *P*-value | HR(95%CI) | *P*-value |
| P4HA2  (high vs low) | 1.174(0.970-1.421) | 0.039* | 1.257(1.031-1.532) | 0.024* |
| Gender  (male vs female) | 0.773(0.564-1.059) | 0.109 | 0.819(0.589-1.140) | 0.237 |
| Age  (>65 vs <=65) | 1.024(1.010-1.038) | <0.001* | 1.028(1.013-1.043) | <0.001* |
| Grade  (Grade II,III,IV vs Grade I) | 1.153(0.917-1.450) | 0.022* | 1.123(0.885-1.423) | 0.340 |
| Stage  (StageII,III,IV vs Stage I) | 1.451(1.205-1.747) | <0.001* | 1.531(1.264-1.853) | <0.001* |
