# Supplementary material for: P4HA2 promotes proliferation, invasion, and metastasis through regulation of the PI3K/AKT signaling pathway in oral squamous cell carcinoma
Source: Sci Rep. 2024 Jul 1;14:15023. doi: 10.1038/s41598-024-64264-5 (PMC11217378; doi:10.1038/s41598-024-64264-5)
Supplement: Supplementary file 3 — Supplementary Figure 1. [file 41598_2024_64264_MOESM3_ESM.docx]

Supplementary Information 3


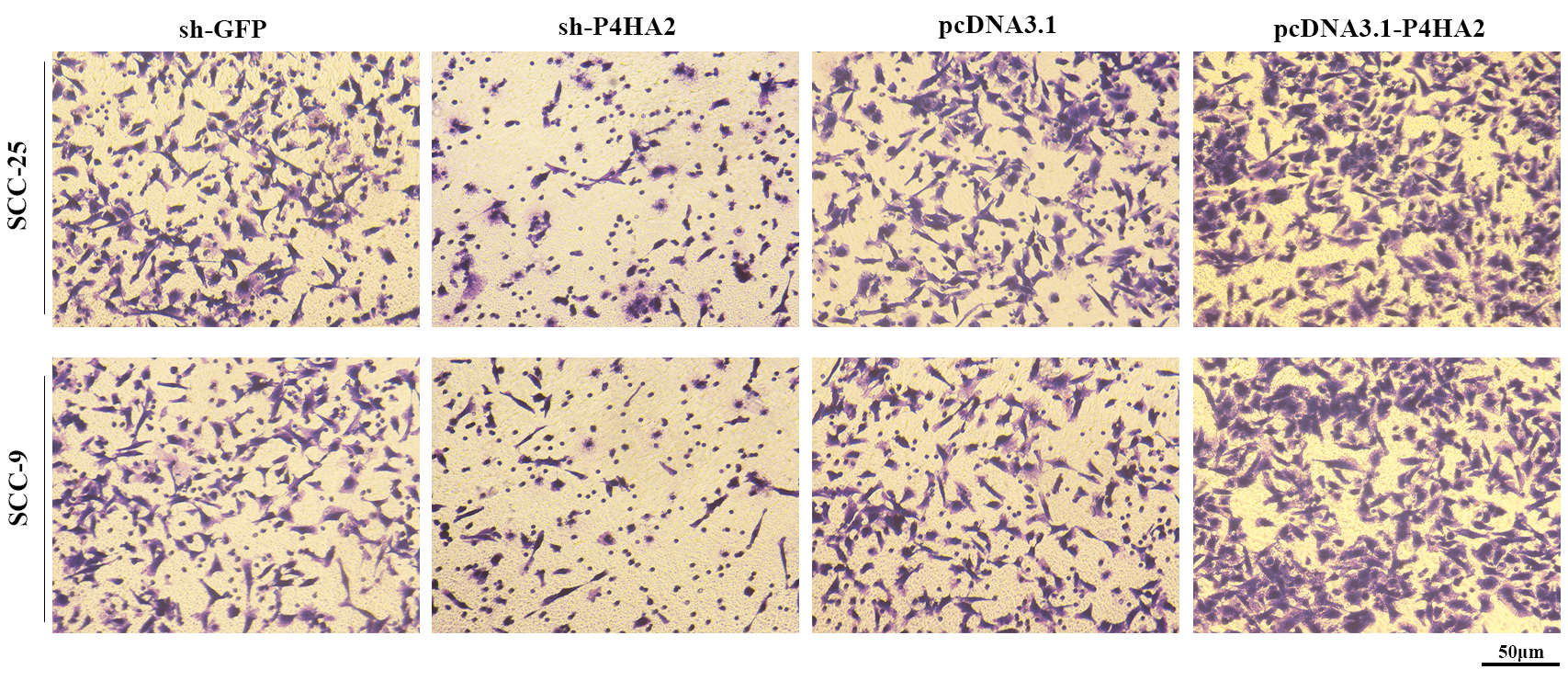


Spplementary Figure 1: Representative 200× images from the cell invasion test.
